# Supplementary material for: Tip carbon encapsulation customizes cationic enrichment and valence stabilization for low K+ acidic CO2 electroreduction
Source: Nat Commun. 2025 Feb 19;16:1754. doi: 10.1038/s41467-025-56977-6 (PMC11839987; doi:10.1038/s41467-025-56977-6)
Supplement: Supplementary file 1 — Supplementary Information [file 41467_2025_56977_MOESM1_ESM.pdf]

Supplementary Information

Tip Carbon Encapsulation Customizes Cationic Enrichment and Valence Stabilization for Low K^+ Acidic CO_2 Electroreduction

Zhitong Wang[1,2,#], Dongyu Liu[3,#], Chenfeng Xia[2,#,*], Xiaodong Shi[1,#], Yansong Zhou[4], Qiuwen Liu[5], Jiangtao Huang[5], Haiyan Wu[1], Deyu Zhu[2], Shuyu Zhang[4], Jing Li[1], Peilin Deng[1], Andrey S. Vasenko[3,6], Bao Yu Xia[2,*], and Xinlong Tian[1,*]

[1] School of Marine Science and Engineering, Hainan University, Haikou 570228, China

[2] School of Chemistry and Chemical Engineering, State Key Laboratory of Materials Processing and Die & Mould Technology, Key Laboratory of Material Chemistry for Energy Conversion and Storage (Ministry of Education), Hubei Key Laboratory of Material Chemistry and Service Failure, Wuhan National Laboratory for Optoelectronics, Huazhong University of Science and Technology (HUST), 1037 Luoyu Rd, Wuhan 430074, China

[3] HSE University, 101000 Moscow, Russia

[4] State Key Laboratory of Photovoltaic Science and Technology, Institute for Electric Light Sources, School of Information Science and Technology, Fudan University, Shanghai, 200433, China.

[5] Hunan Joint International Research Center for Carbon Dioxide Resource Utilization, School of Physics, School of Materials Science & Engineering, Hunan Provincial Key Laboratory of Electronic Packaging and Advanced Functional Materials of Hunan Province, Central South University, Changsha, 410083, Hunan P. R. China

[6] Donostia International Physics Center (DIPC), 20018 San Sebastián-Donostia, Euskadi, Spain

[#] Z. Wang, D. Liu, C. Xia. and X. Shi contributed equally to this work.

*Corresponding authors: cfxia@hust.edu.cn (C. Xia); byxia@hust.edu.cn (B. Y. Xia); tianxl@hainanu.edu.cn (X. Tian)

Table of Content

Figures...3

Tables……………………………………………………………………………………………46

References………………………………………………………………………………………48

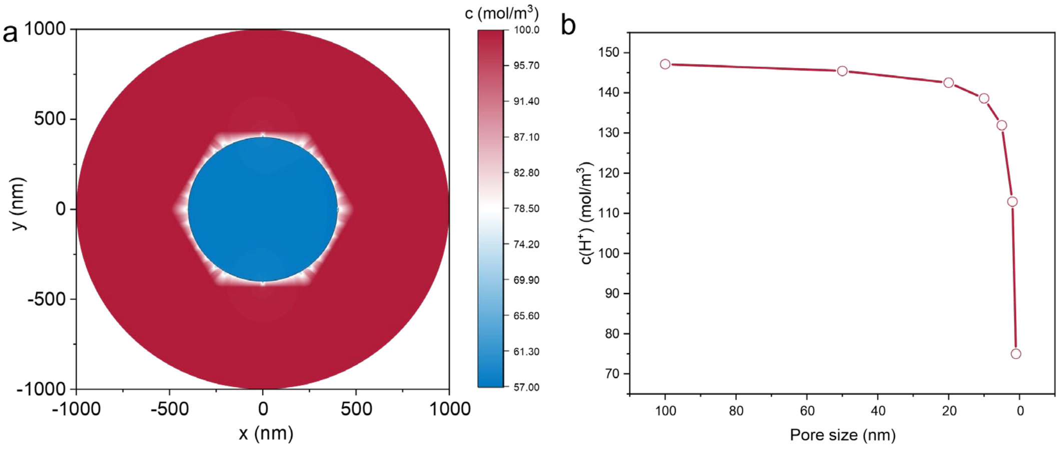

Supplementary Fig. 1 (a) Simulation model used to study the alkaline microenvironment within the cavity of the hollow In_2O_3 nanoparticles and (b) its dependence on the pore size. The left panel corresponds to the condition with the pore size of 1 nm. Source data for Supplementary Fig. 1 are provided as a Source Data file.

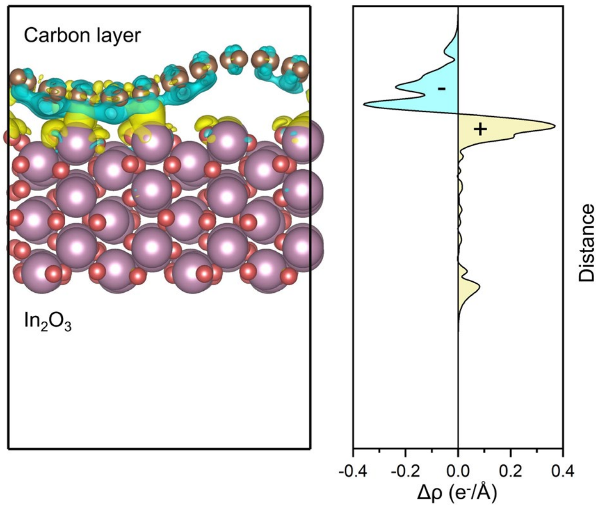

Supplementary Fig. 2 Side view of the charge density difference at carbon layer/In_2O_3 complex model and the plane-averaged results along the perpendicular direction. The graphene is curved due to lattice mismatch. Color code: purple for In, red for O, and brown for C. The blue and yellow regions indicate electron loss and gain, respectively. Source data for Supplementary Fig. 2 are provided as a Source Data file.

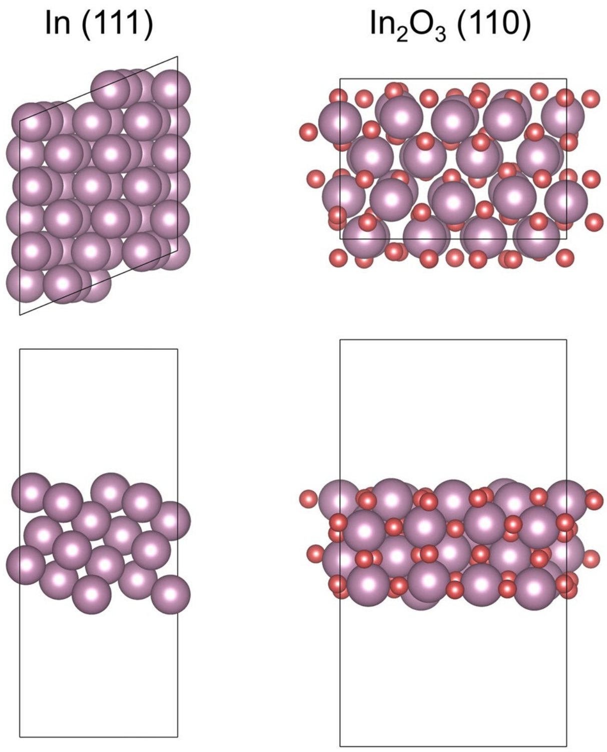

Supplementary Fig. 3 Optimized structures of In (111) and In_2O_3 (110). The color code is the same as Supplementary Fig. 2.

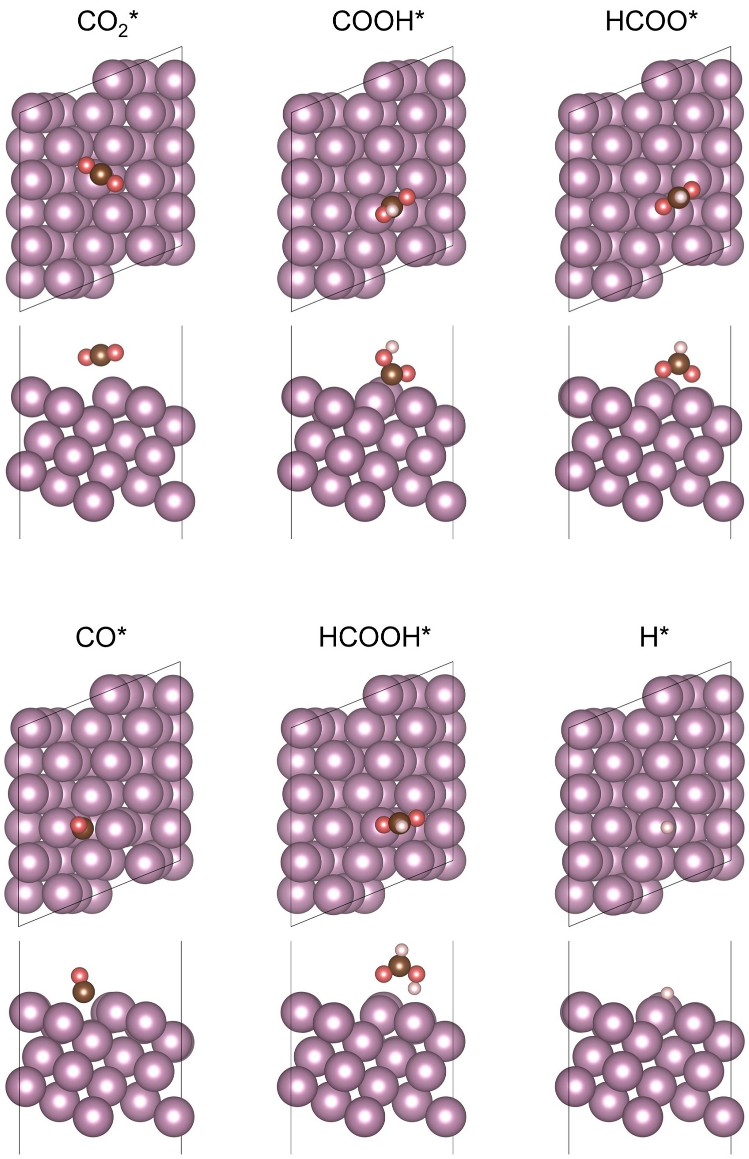

Supplementary Fig. 4 Adsorption configurations of reaction intermediates on In (111) surface. The color code is the same as Supplementary Fig. 2.

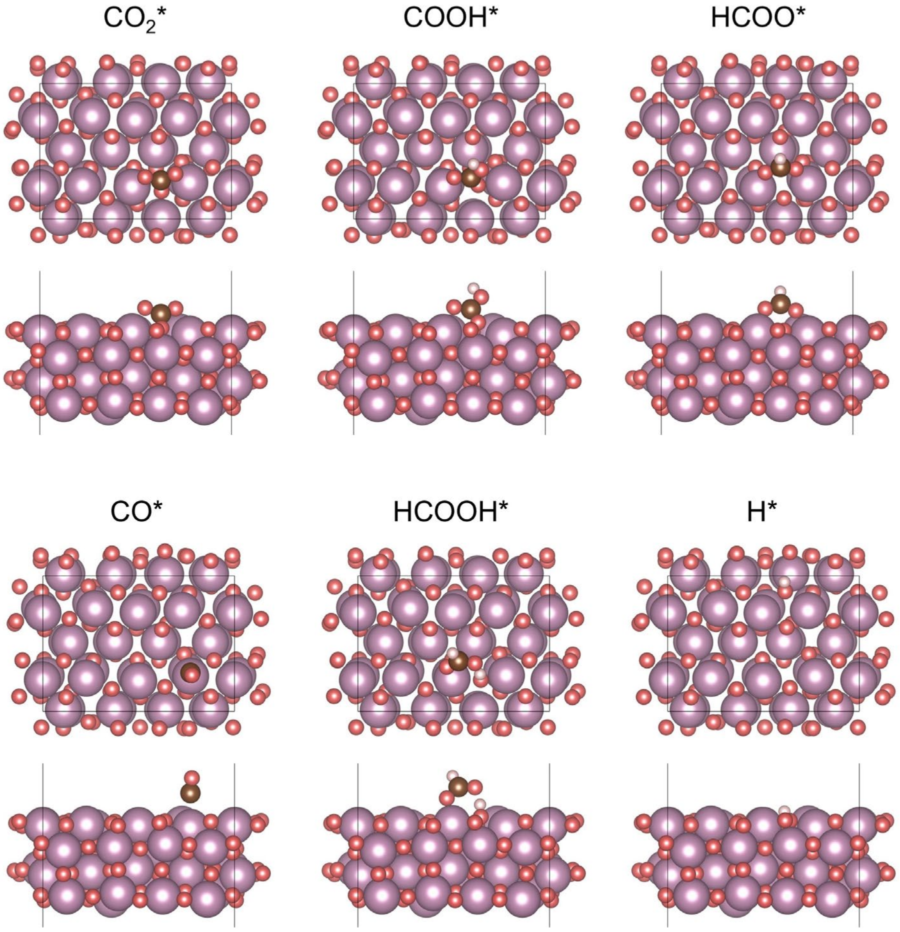

Supplementary Fig. 5 Adsorption configurations of reaction intermediates on In_2O_3 (110) surface. The color code is the same as Supplementary Fig. 2.

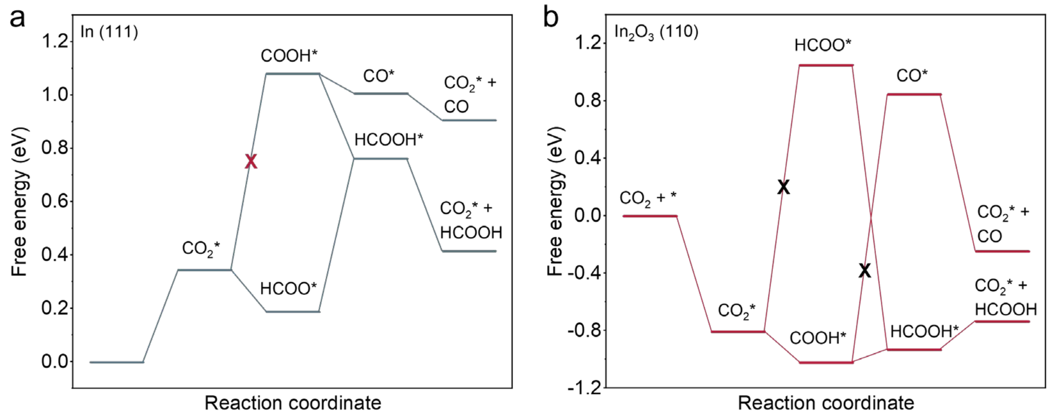

Supplementary Fig. 6 Free energy diagram of CO_2RR on (a) In (111) and (b) In_2O_3 (110). The CO path is thermodynamically unfavorable on both surfaces. Source data for Supplementary Fig. 6 are provided as a Source Data file.

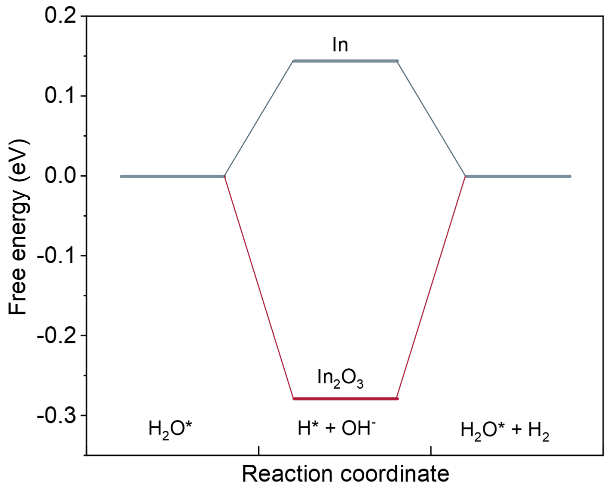

Supplementary Fig. 7 Free energy diagram of HER on In (111) and In_2O_3 (110). Source data for Supplementary Fig. 7 are provided as a Source Data file.

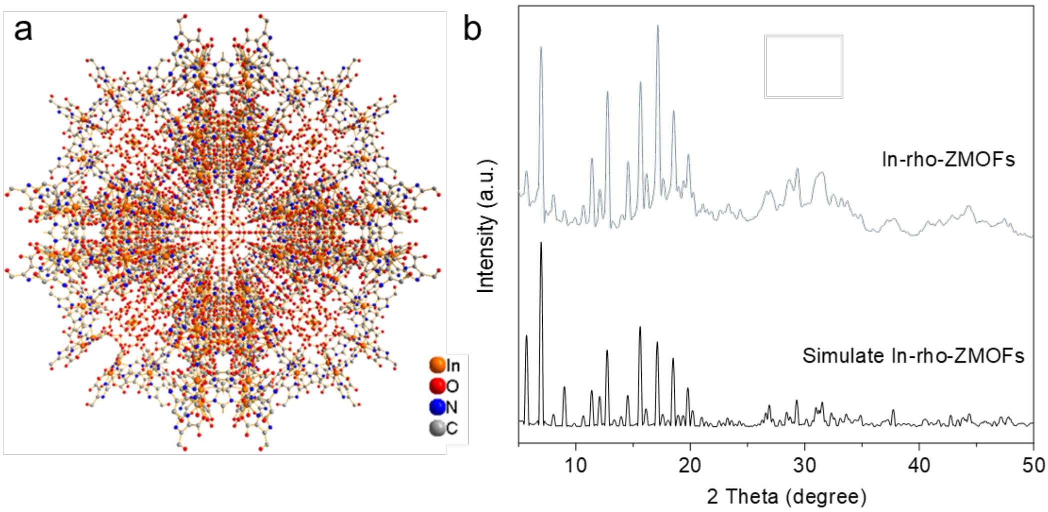

Supplementary Fig. 8 (a) Structural model and (b) XRD pattern of In-rho-ZMOF. Source data for Supplementary Fig. 8b are provided as a Source Data file.

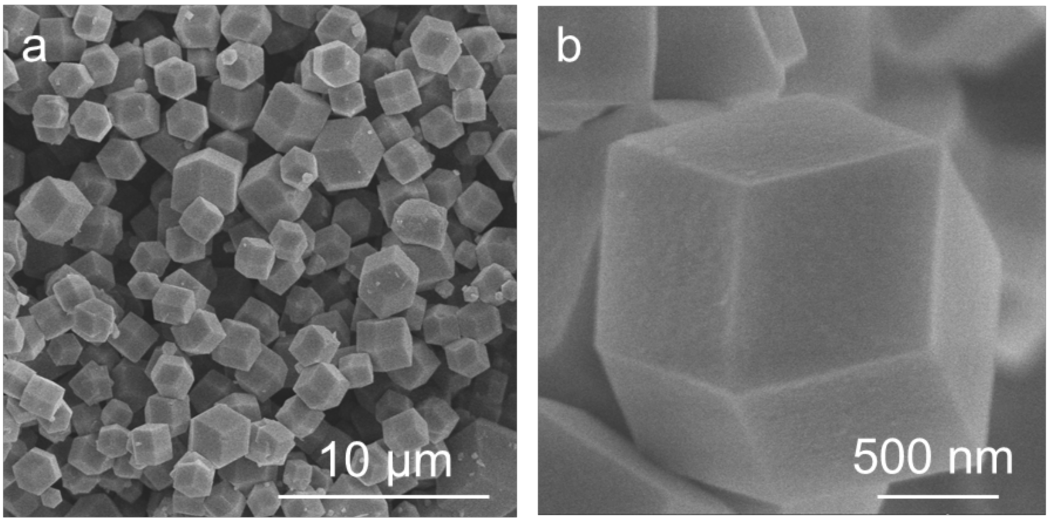

Supplementary Fig. 9 (a, b) SEM images of In-rho-ZMOF.

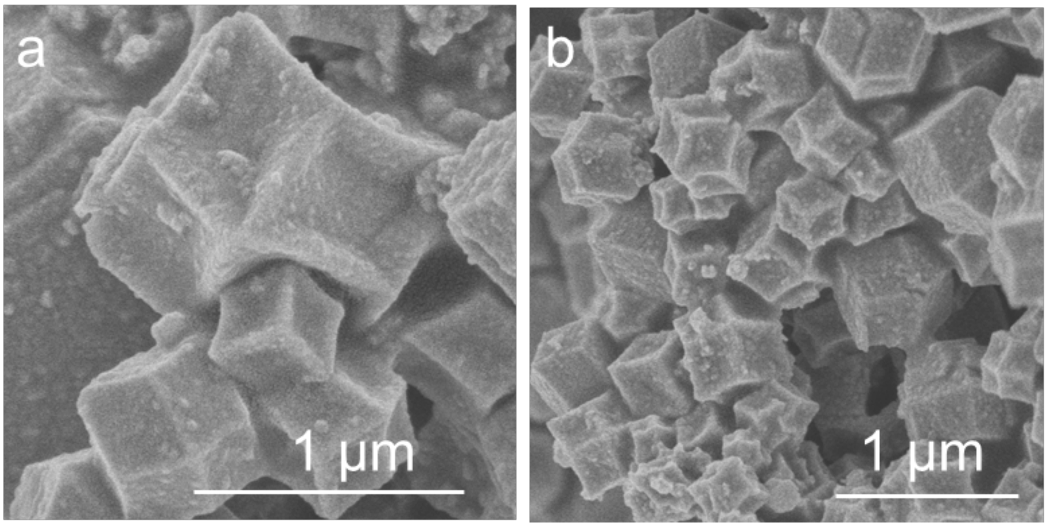

Supplementary Fig. 10 (a, b) SEM images of Vac.

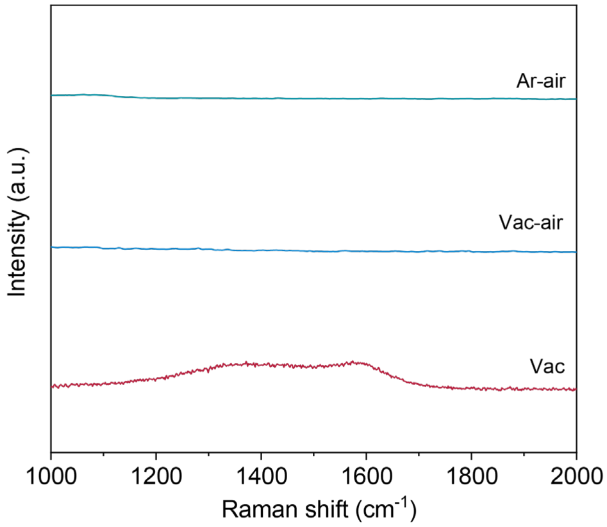

Supplementary Fig. 11 Raman spectra of Vac, Vac-air and Ar-air. Source data for Supplementary Fig. 11 are provided as a Source Data file.

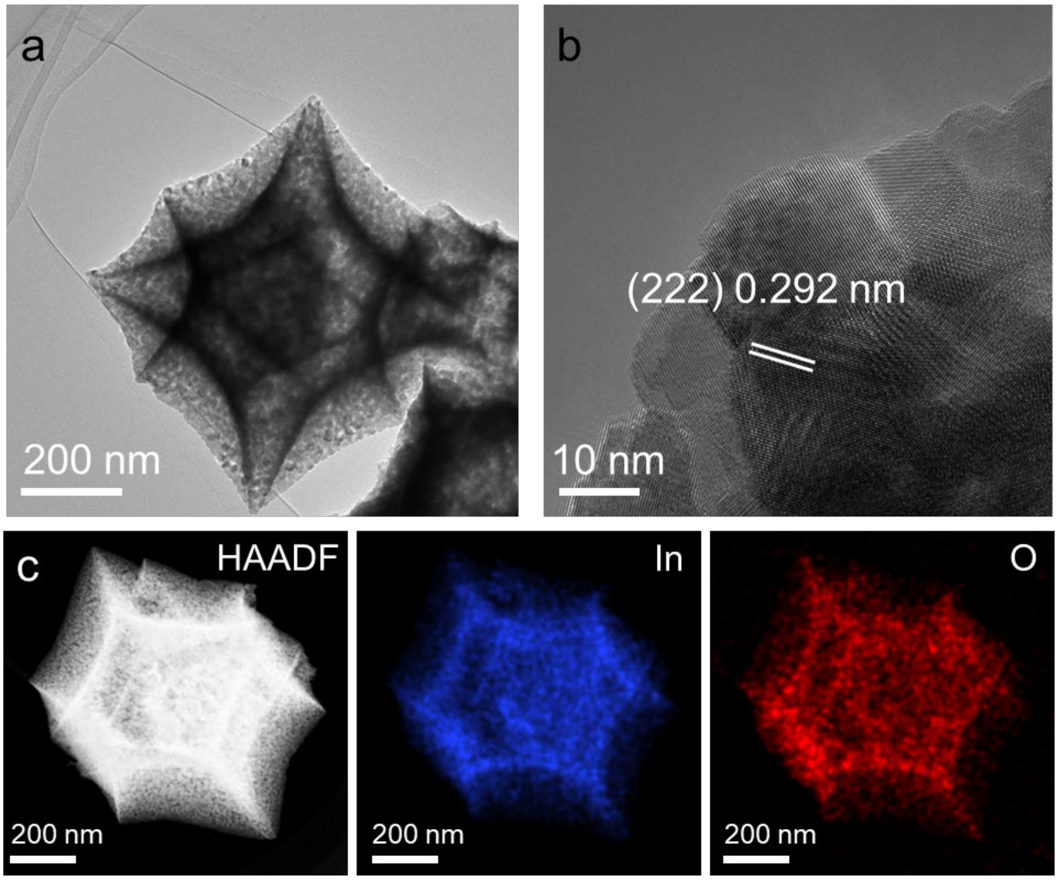

Supplementary Fig. 12 (a, b) TEM images and (c) EDS elemental mapping of Vac-air.

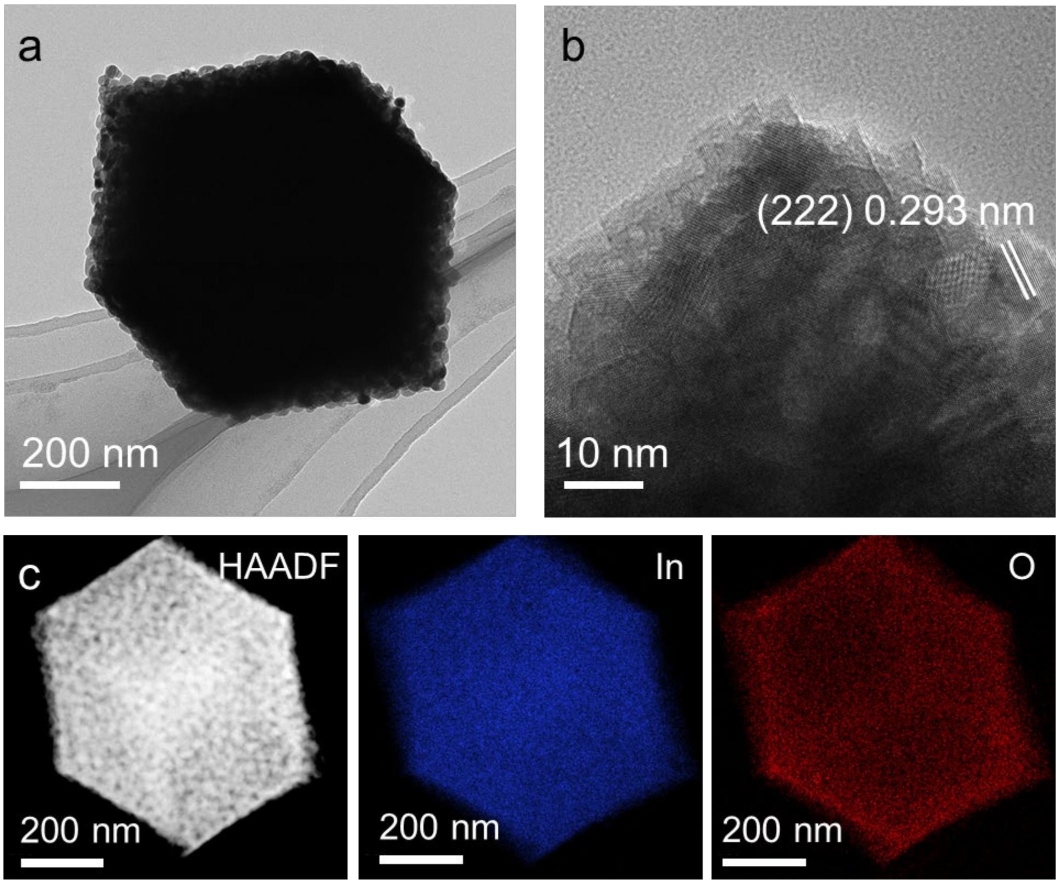

Supplementary Fig. 13 (a, b) TEM images and (c) EDS elemental mapping of Ar-air.

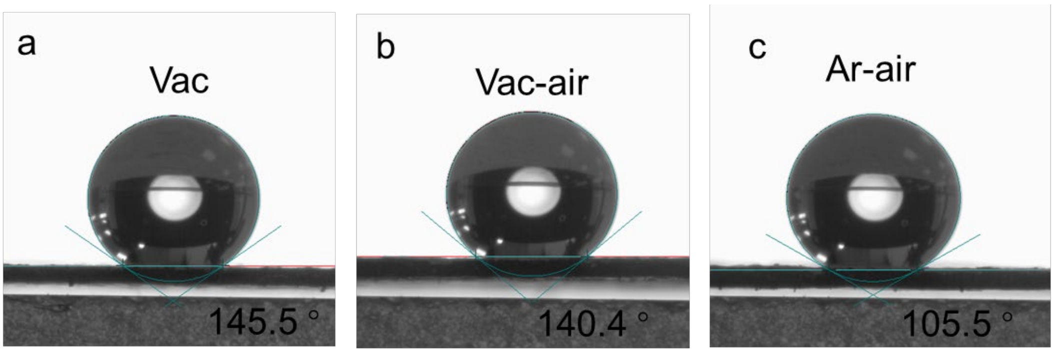

Supplementary Fig. 14 Contact angle measurements of (a) Vac, (b) Vac-air and (c) Ar-air.

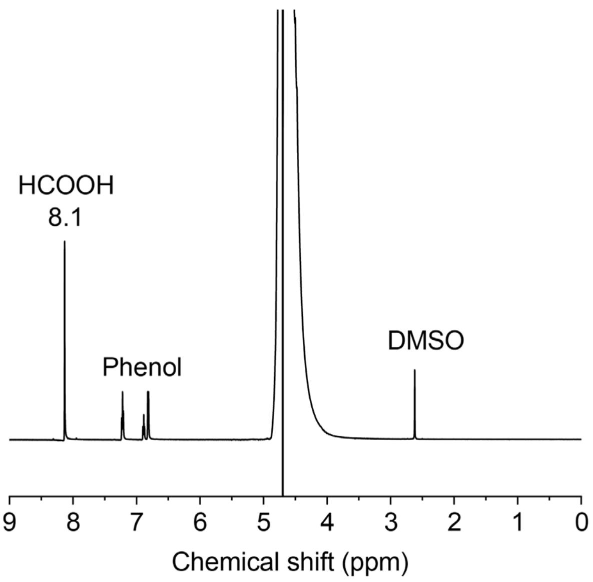

Supplementary Fig. 15 ^{1}H-NMR spectrum of liquid products and internal standard substance (DMSO). Source data for Supplementary Fig. 15 are provided as a Source Data file.

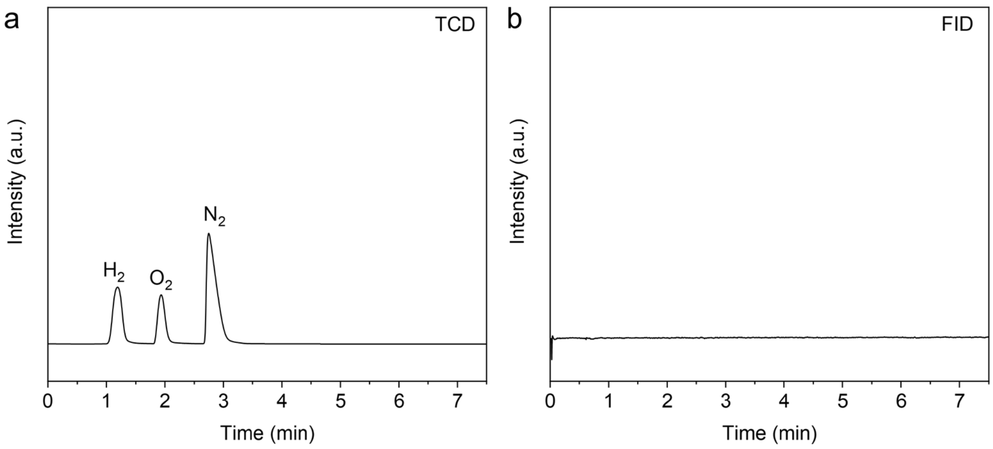

Supplementary Fig. 16 GC measurement of gas products via (a) TCD detector, (b) FID detector. Source data for Supplementary Fig. 16 are provided as a Source Data file.

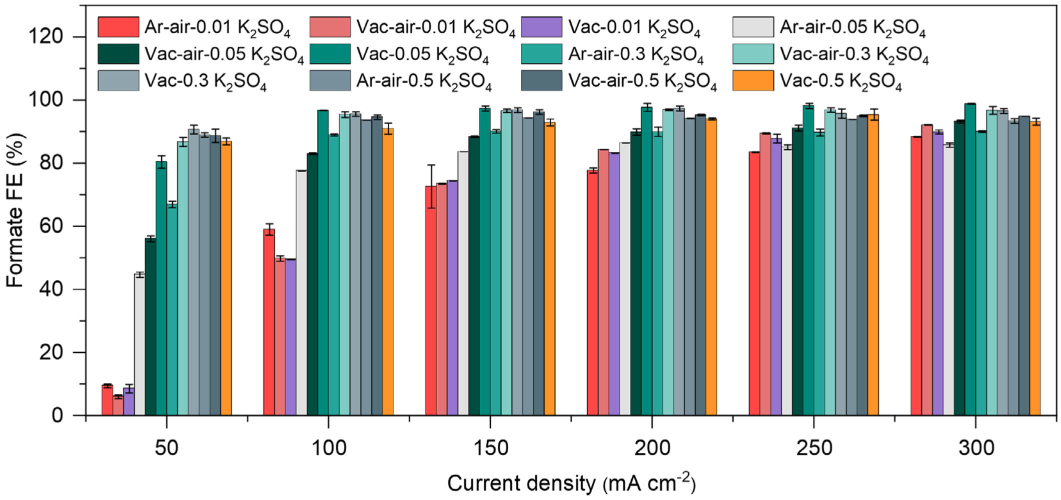

Supplementary Fig. 17 Current-dependent HCOOH FE of Vac, Vac-air and Ar-air in 0.05 M H_2SO_4 electrolyte with different K^+ concentrations. Error bars represent the standard deviation of three independent measurements. Source data for Supplementary Fig. 17 are provided as a Source Data file.

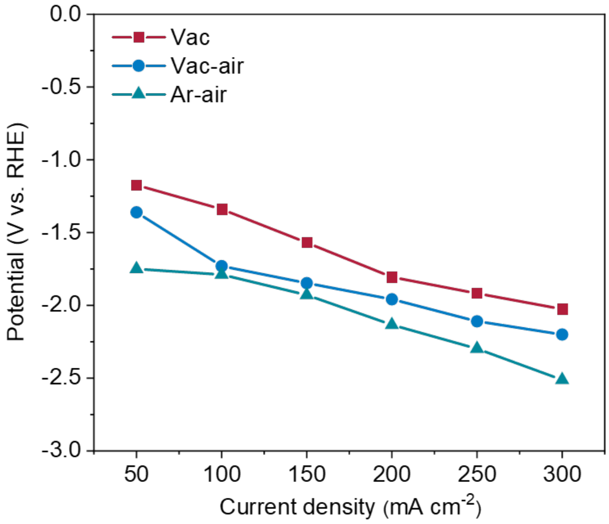

Supplementary Fig. 18 Current-dependent potential plots of Vac, Vac-air and Ar-air in 0.05 M H_2SO_4 electrolyte with 0.1 M K^+. The potentials were compensated with iR_Ω correction. Source data for Supplementary Fig. 18 are provided as a Source Data file.

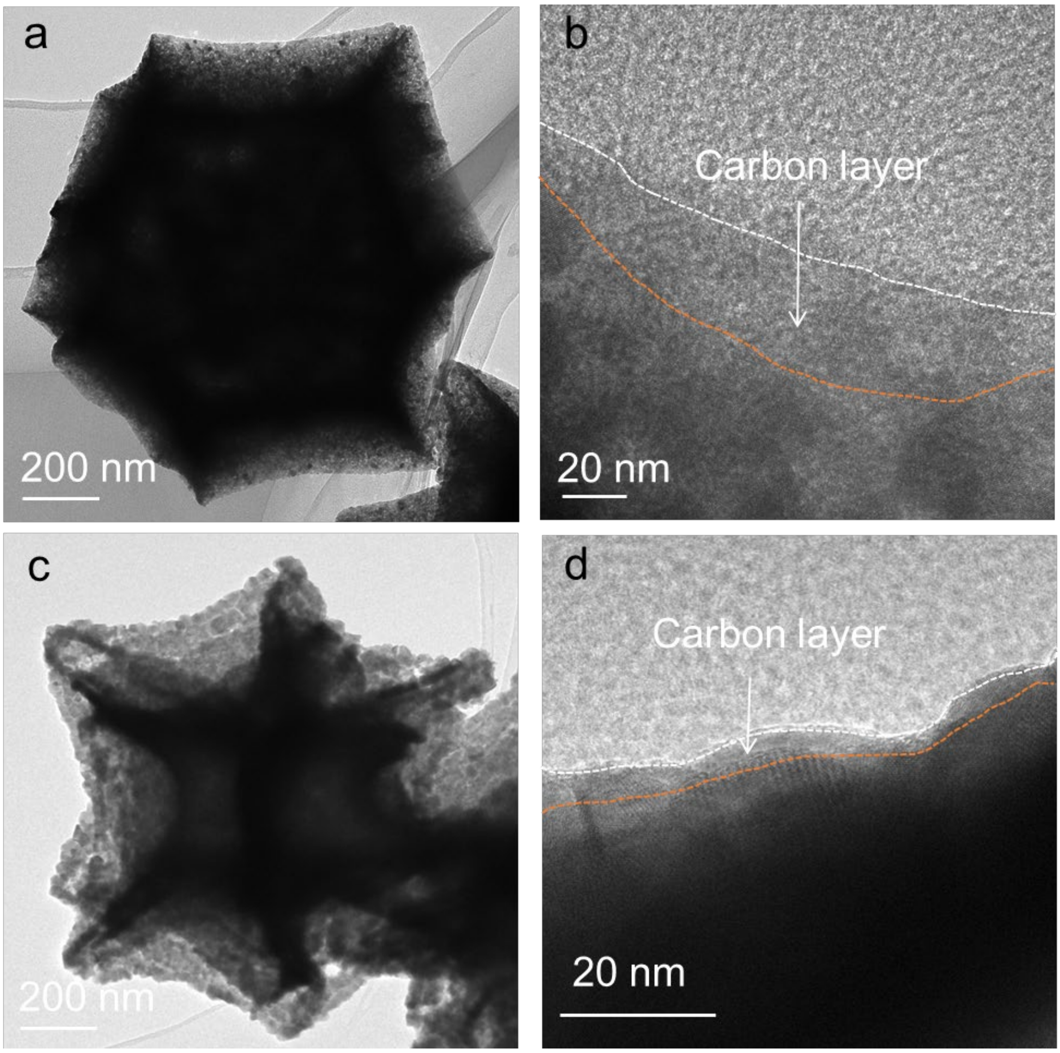

Supplementary Fig. 19 TEM images of (a) Vac-1 and (c) Vac-2. HRTEM images of (b) Vac-1 and (d) Vac-2.

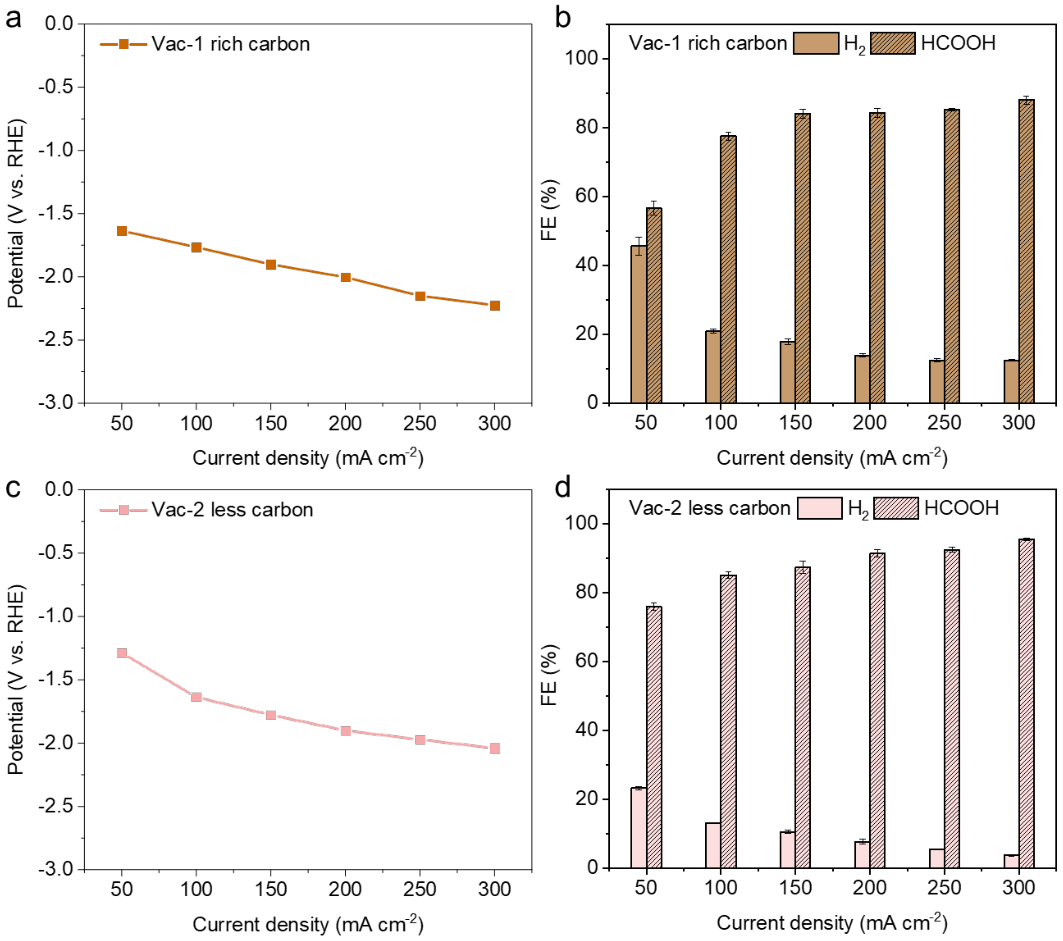

Supplementary Fig. 20 Current-dependent potential plots of (a) Vac-1 and (c) Vac-2. Current-dependent FE of (b) Vac-1 and (d) Vac-2. Error bars represent the standard deviation of three independent measurements. The potentials were compensated with iR_Ω correction. Source data for Supplementary Fig. 20 are provided as a Source Data file.

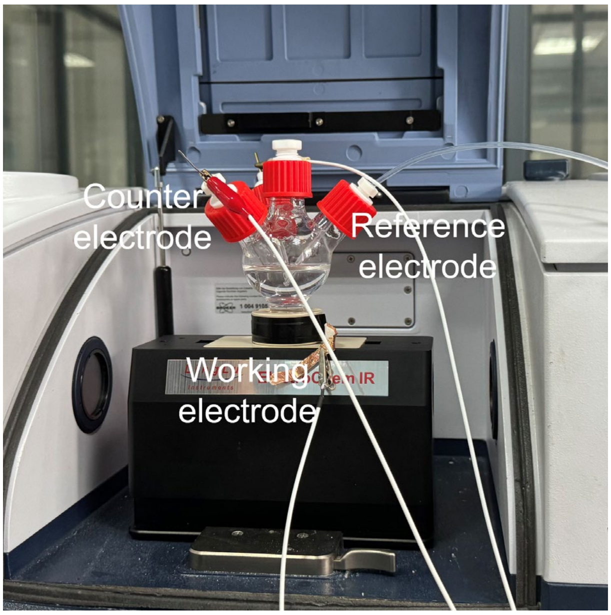

Supplementary Fig. 21 Photograph of in situ ATR-SEIRAS test device.

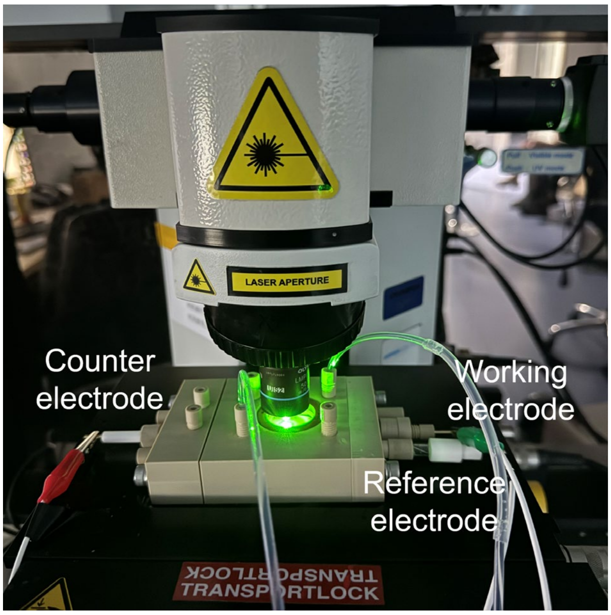

Supplementary Fig. 22 Photograph of in situ Raman test device.

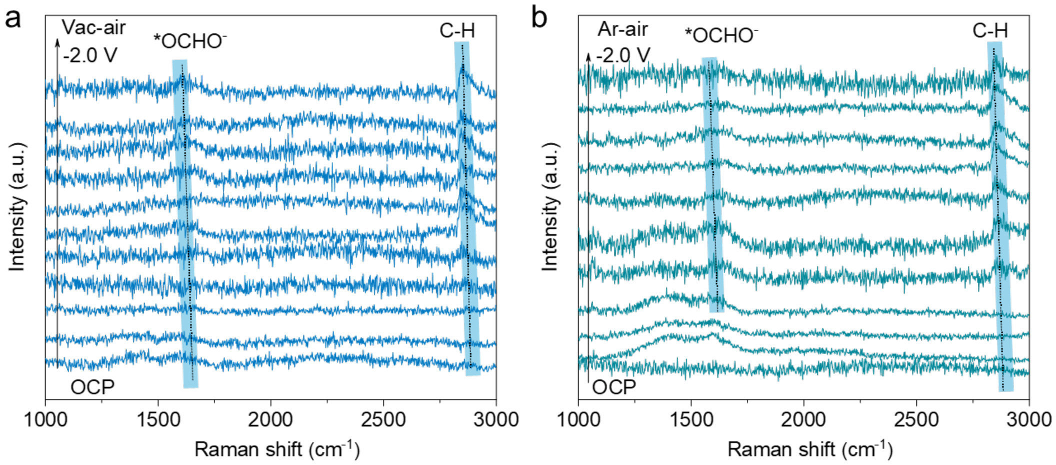

Supplementary Fig. 23 In situ Raman spectra of Vac-air and Ar-air from OCP to −2.0 V vs RHE. The potentials for in situ measurements were without iR_{Ω} correction. Source data for Supplementary Fig. 23 are provided as a Source Data file.

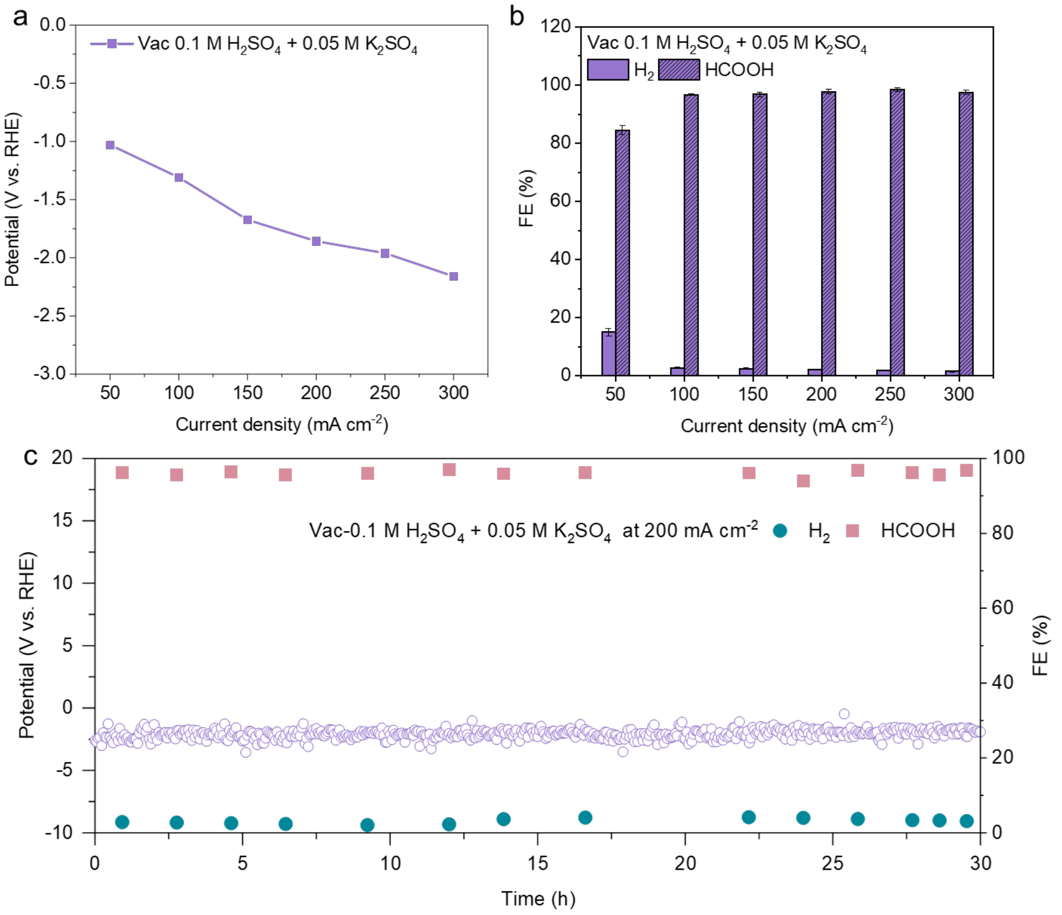

Supplementary Fig. 24 (a) Current-dependent potential plots, (b) current-dependent FE and long-term stability test of Vac in 0.1 M H_2SO_4 with 0.1 M K^+. Error bars represent the standard deviation of three independent measurements. The potentials were compensated with iR_Ω correction. Source data for Supplementary Fig. 24 are provided as a Source Data file.

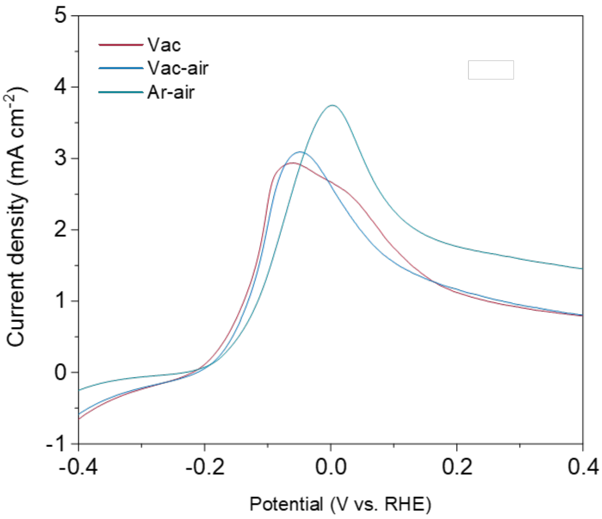

Supplementary Fig. 25 Electrochemical single oxidative curves in the Ar-saturated 1.0 M KOH of Vac, Vac-air and Ar-air. There is no iR_{Ω} correction for applied potentials. Source data for Supplementary Fig. 25 are provided as a Source Data file.

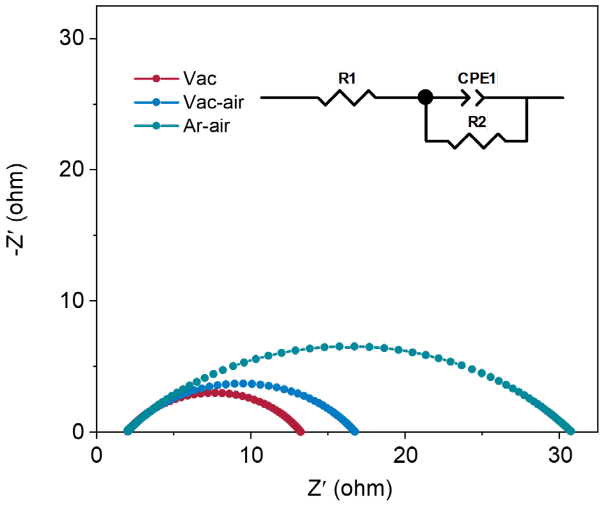

Supplementary Fig. 26 EIS curves and equivalent circuit (inset) of Vac, Vac-air and Ar-air. Source data for Supplementary Fig. 26 are provided as a Source Data file.

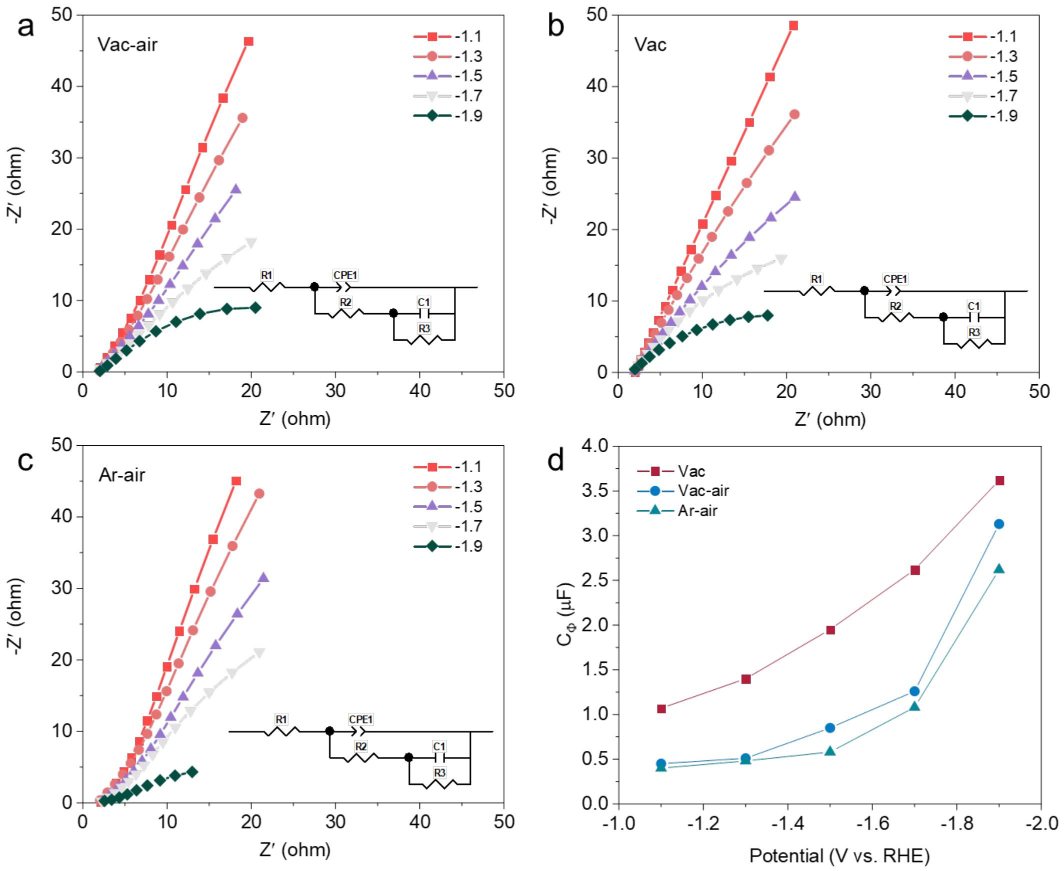

Supplementary Fig. 27 (a-c) EIS curves of Vac, Vac-air and Ar-air recorded at different applied potentials and (d) corresponding potential-dependent Cϕ plots using a double-parallel equivalent circuit fitting model. There is no iR_{Ω} correction for applied potentials. Source data for Supplementary Fig. 27 are provided as a Source Data file.

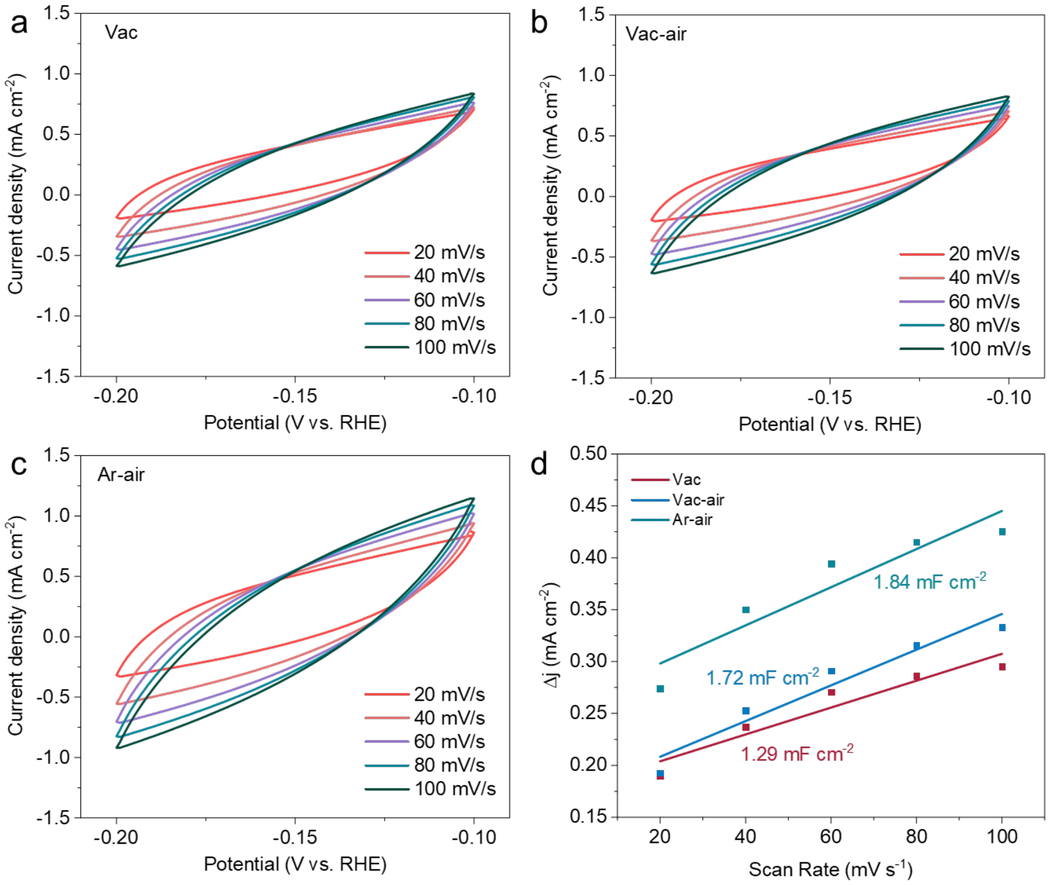

Supplementary Fig. 28 CV curves of (a) Vac, (b) Vac-air, (c) Ar-air in CO_2-saturated 0.1 M $KHCO_3$ at different scan rates of 20, 40, 60, 80, and 100 mV s^{-1}, and (e) capacitive currents at –0.15 V vs. RHE with different sweep rates. There is no iR_{Ω} correction for applied potentials. Source data for Supplementary Fig. 28 are provided as a Source Data file.

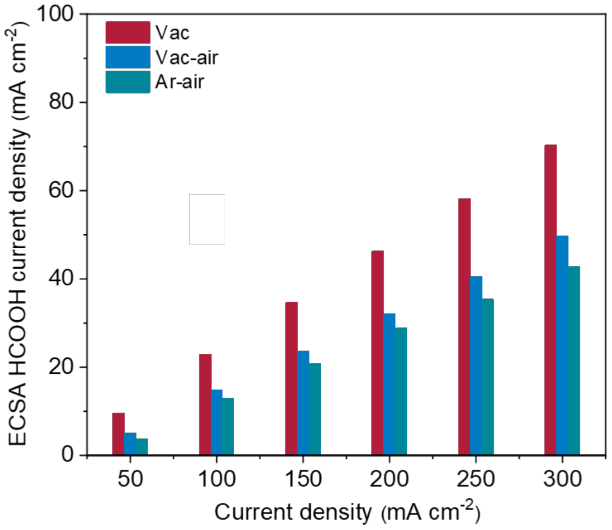

Supplementary Fig. 29 ECSA-normalized partial current density toward formate production for Vac, Vac-air and Ar-air. Source data for Supplementary Fig. 29 are provided as a Source Data file.

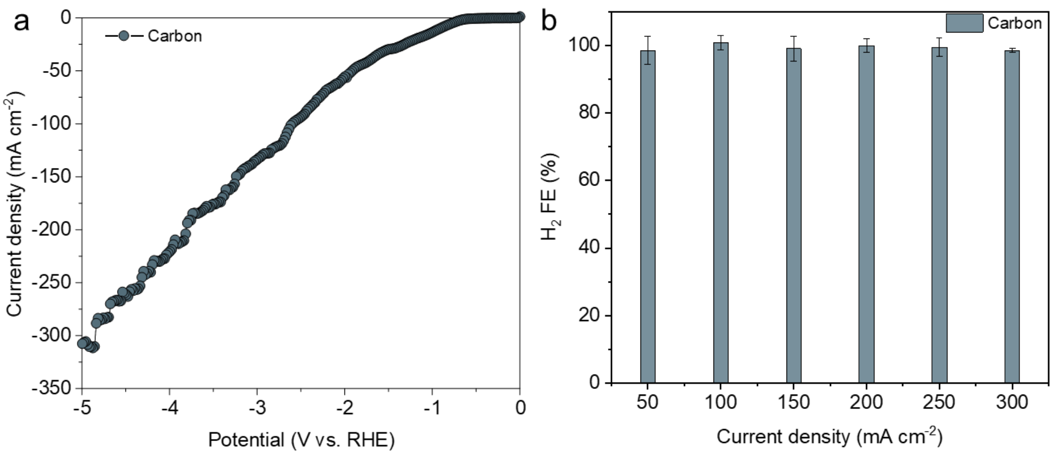

Supplementary Fig. 30 (a) LSV curves and (b) current-dependent H_2 FE of bare carbon in 0.05 M H_2SO_4 with 0.1 M K^+ under CO_2 atmosphere. Error bars represent the standard deviation of three independent measurements. There is no iR_Ω correction for applied potentials. Source data for Supplementary Fig. 30 are provided as a Source Data file.

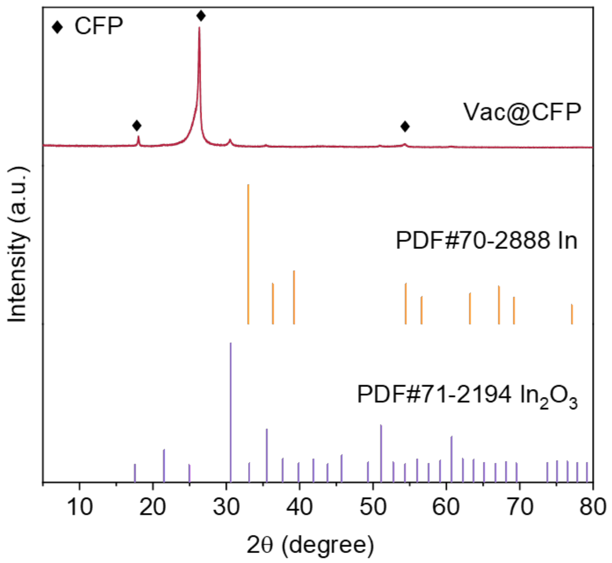

Supplementary Fig. 31 XRD patterns of Vac gas diffusion electrode before reaction. Source data for Supplementary Fig. 31 are provided as a Source Data file.

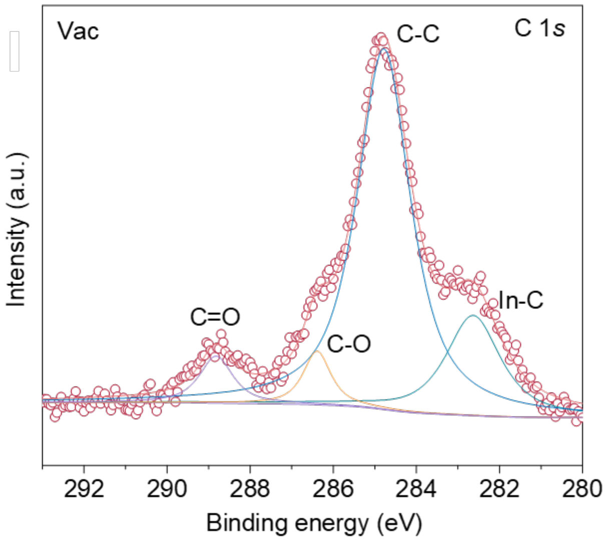

Supplementary Fig. 32 C 1*s* XPS spectra of Vac. Source data for Supplementary Fig. 32 are provided as a Source Data file.

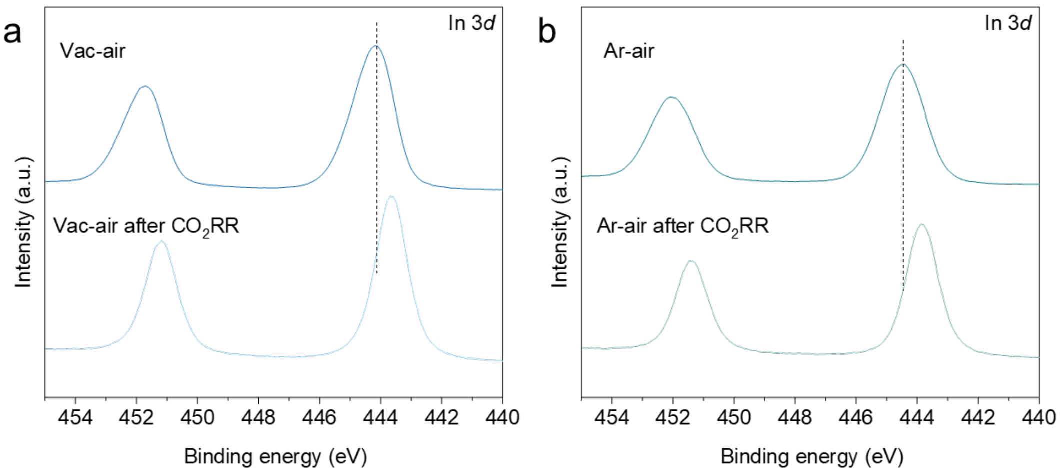

Supplementary Fig. 33 In 3*d* XPS spectra of (a) Vac-air and (b) Ar-air before and after CO_2RR. Source data for Supplementary Fig. 33 are provided as a Source Data file.

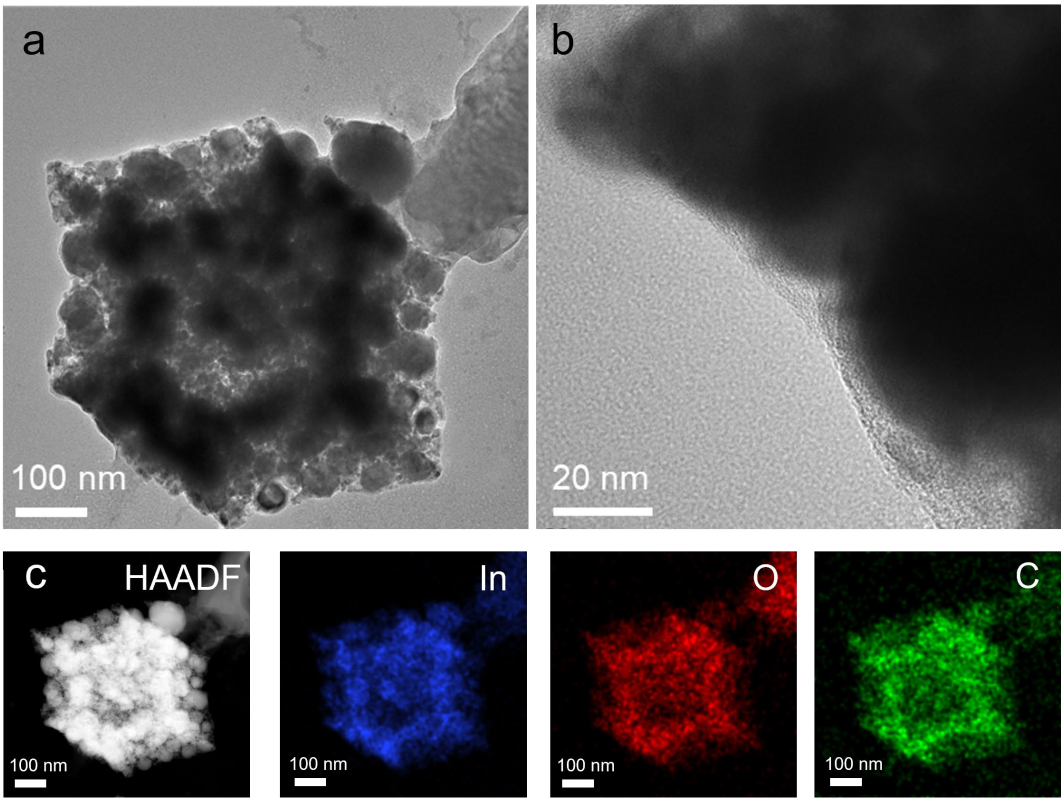

Supplementary Fig. 34 (a, b) TEM images and (c) EDS elemental mapping of Vac after CO_2RR.

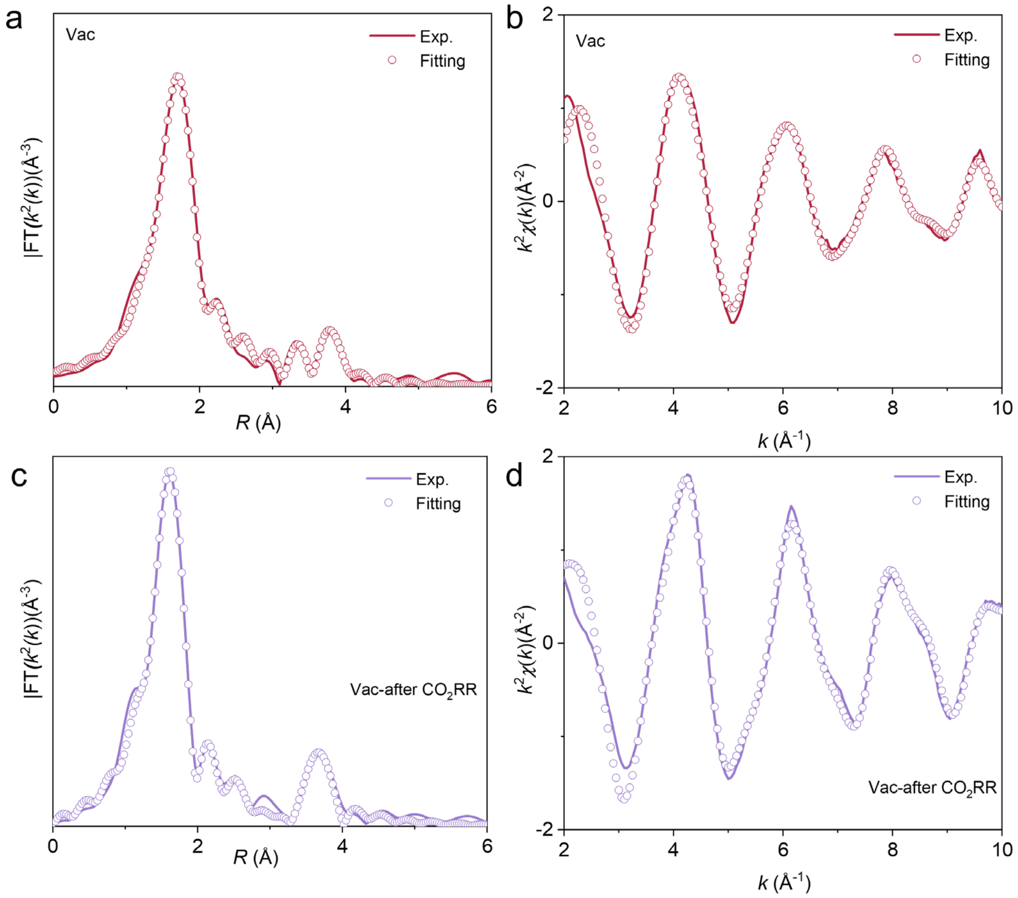

Supplementary Fig. 35 (a) The R-space of In EXAFS fitting spectra and (b) K^{2-}weighted curves of Vac at the In K-edge. (c) The R-space of In EXAFS fitting spectra and (d) K^{2-}weighted curves of Vac after CO_2RR at the In K-edge. Source data for Supplementary Fig. 35 are provided as a Source Data file.

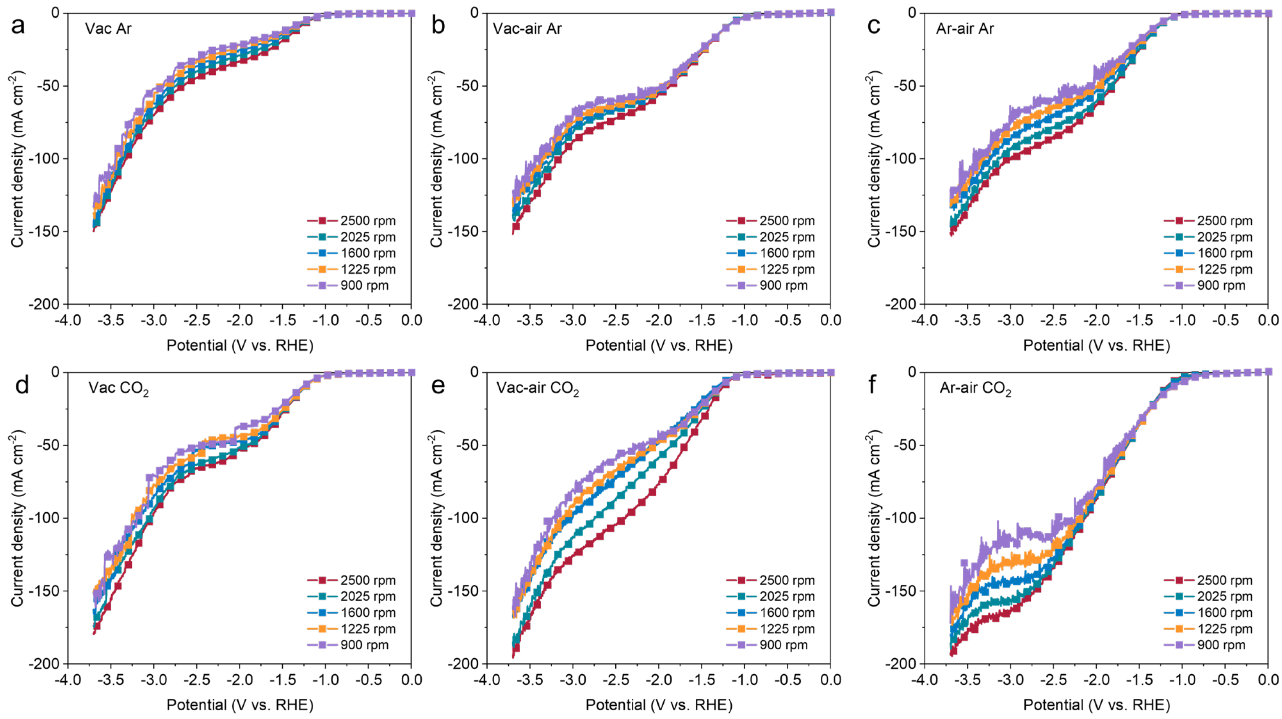

Supplementary Fig. 36 LSV curves of Vac, Vac-air and Ar-air in (a-c) Ar and (d-f) CO_2-saturated 0.05 M H_2SO_4 with 0.1 M K^+ under varied rotating speed. There is no iR_Ω correction for applied potentials. Source data for Supplementary Fig. 36 are provided as a Source Data file.

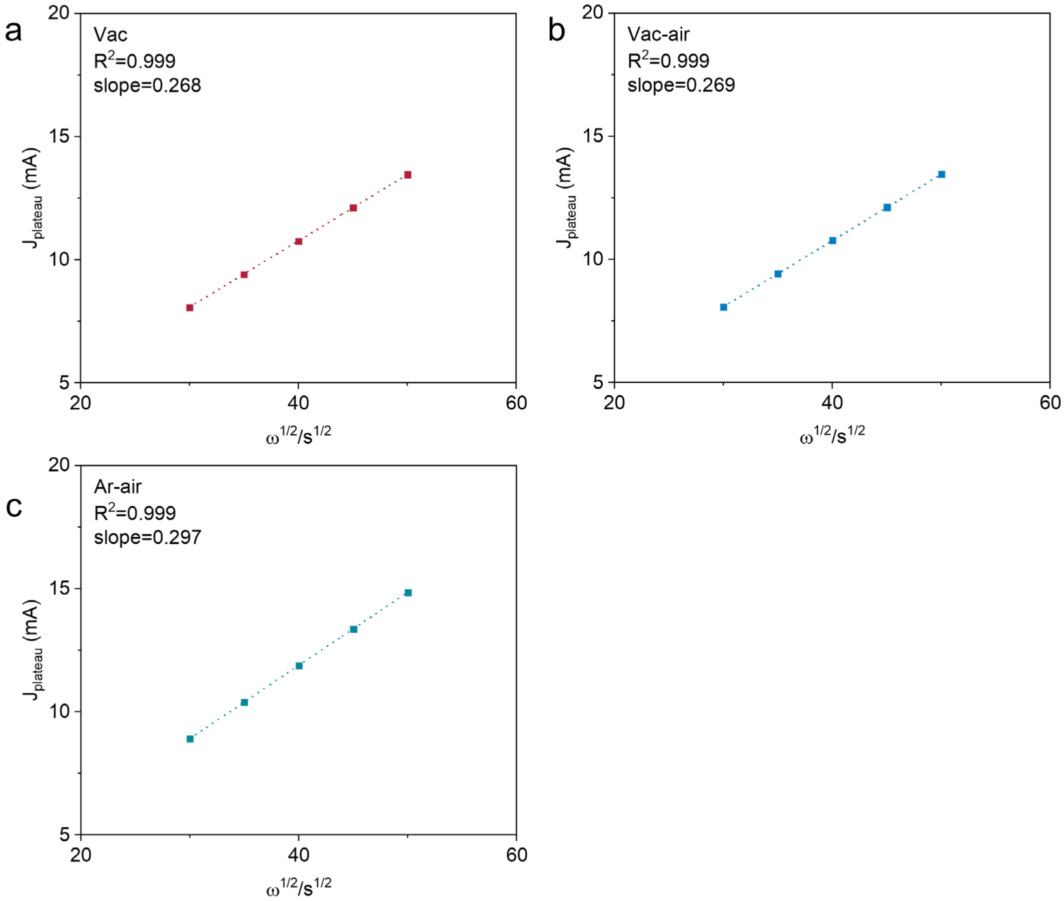

Supplementary Fig. 37 Linear fitting of j_{plateau} verse $\omega^{1/2}$ for (a) Vac, (b) Vac-air, (c) Ar-air based on Levich equation. Source data for Supplementary Fig. 37 are provided as a Source Data file.

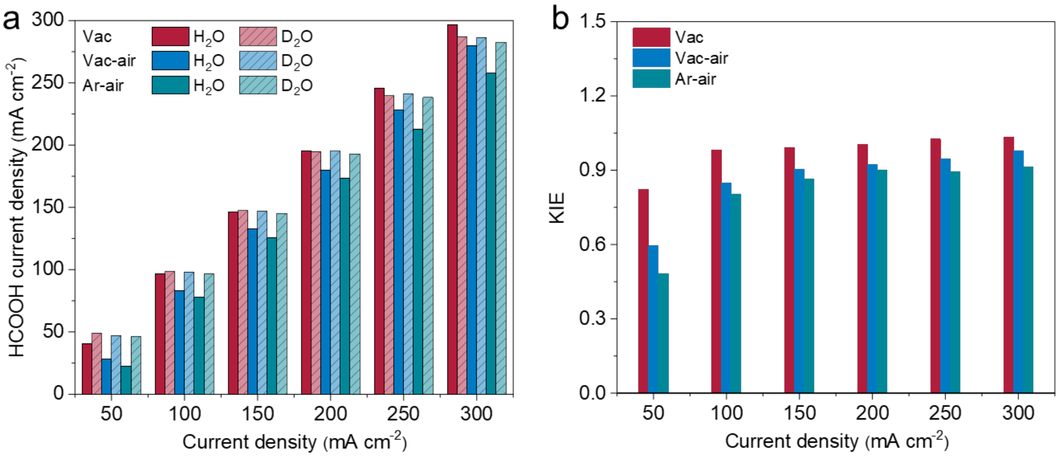

Supplementary Fig. 38 (a) The HCOOH partial current density of Vac, Vac-air and Ar-air in electrolyte with H_2O and D_2O, (b) corresponding KIE values under different applied current densities. Source data for Supplementary Fig. 38 are provided as a Source Data file.

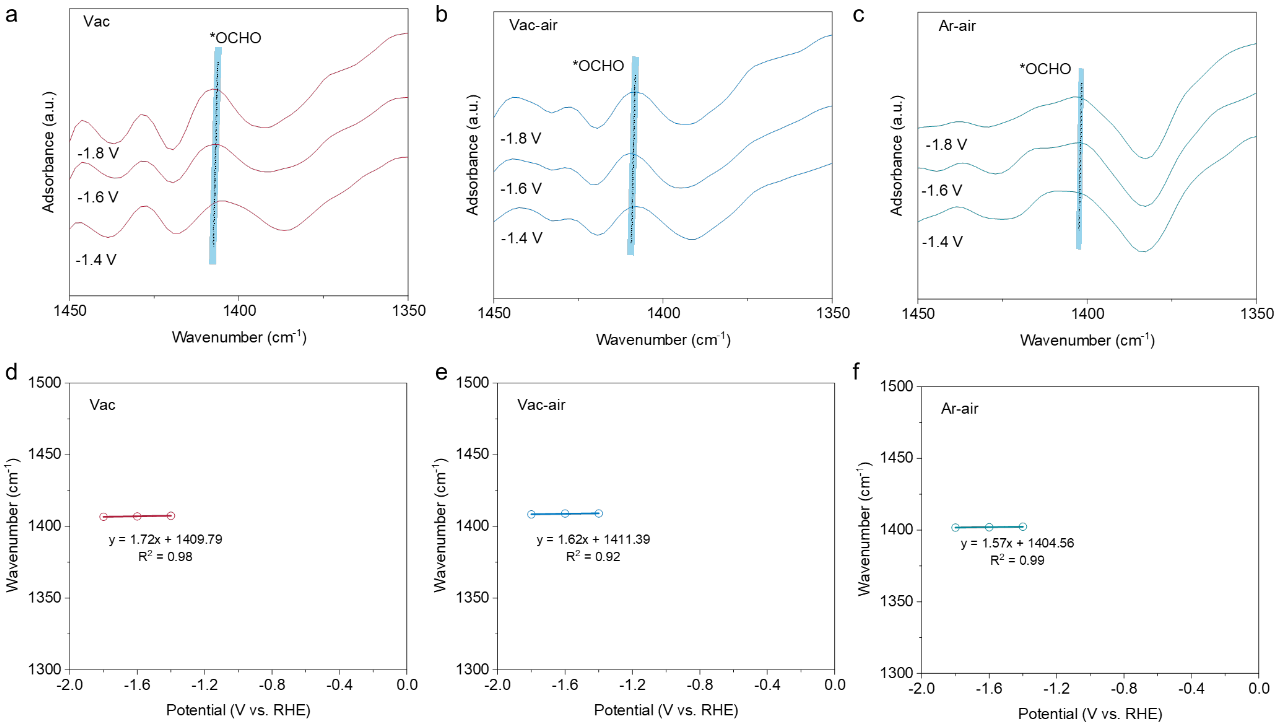

Supplementary Fig. 39 In situ ATR-SEIRAS spectra of (a) Vac, (b) Vac-air, (c) Ar-air. Stark tuning behavior of (d) Vac, (e) Vac-air, (f) Ar-air. There is no iR_{Ω} correction for applied potentials. Source data for Supplementary Fig. 39 are provided as a Source Data file.

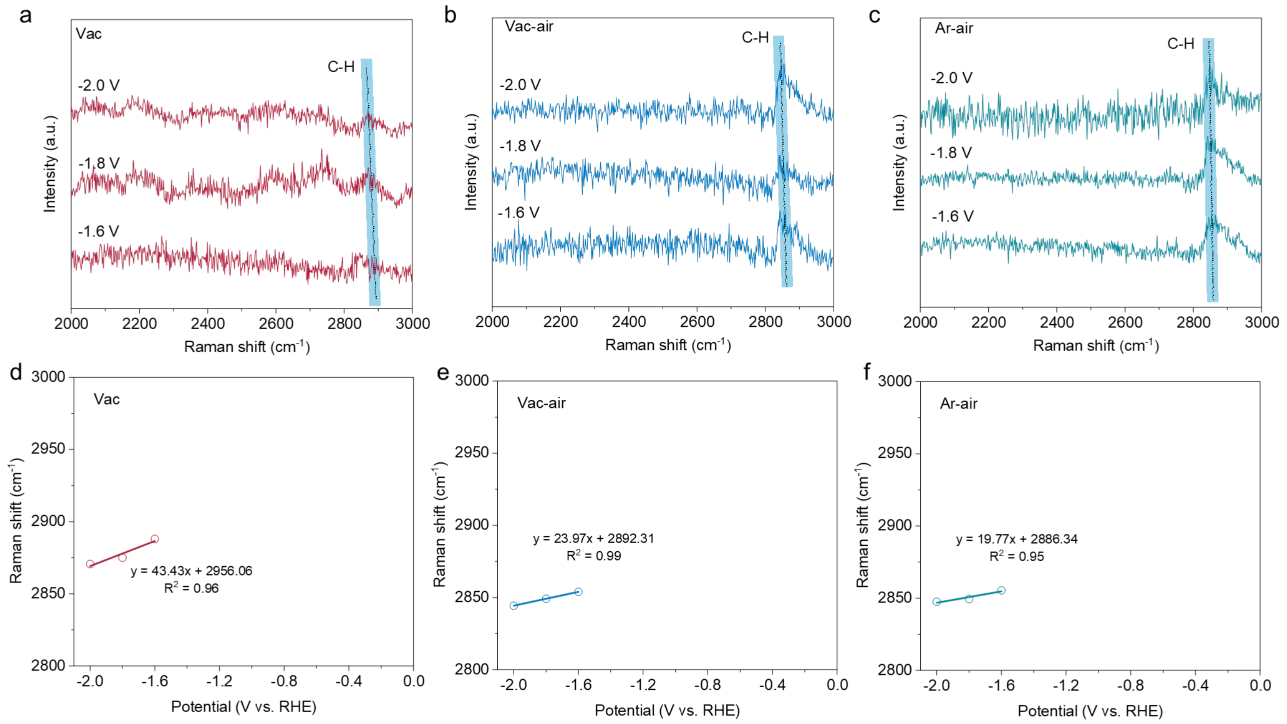

Supplementary Fig. 40 In situ Raman spectra of (a) Vac, (b) Vac-air, (c) Ar-air. Stark tuning behavior of (d) Vac, (e) Vac-air, (f) Ar-air. There is no iR_{Ω} correction for applied potentials. Source data for Supplementary Fig. 40 are provided as a Source Data file.

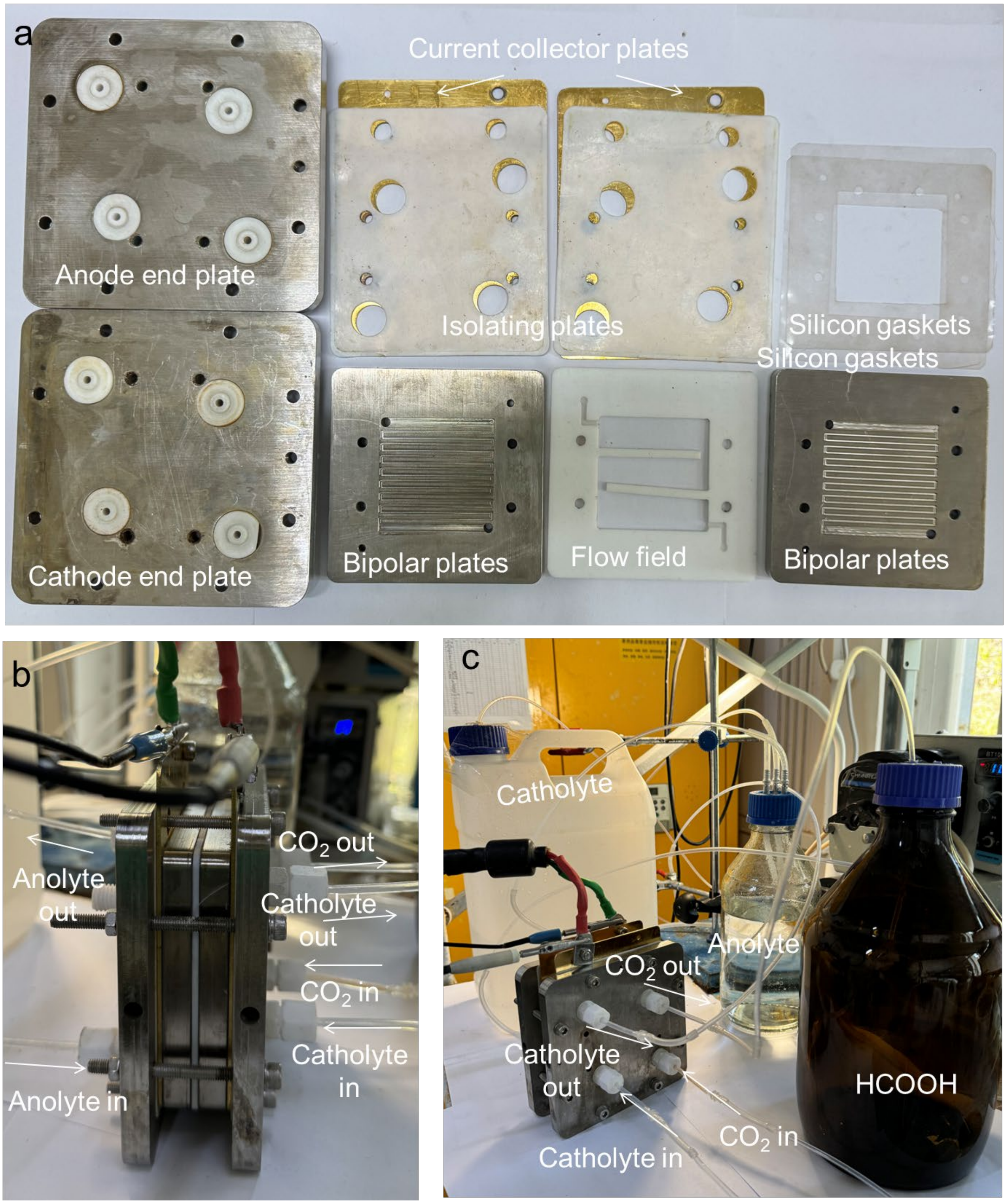

Supplementary Fig. 41 Photographs of the two-electrode scale-up electrolyzer configuration.

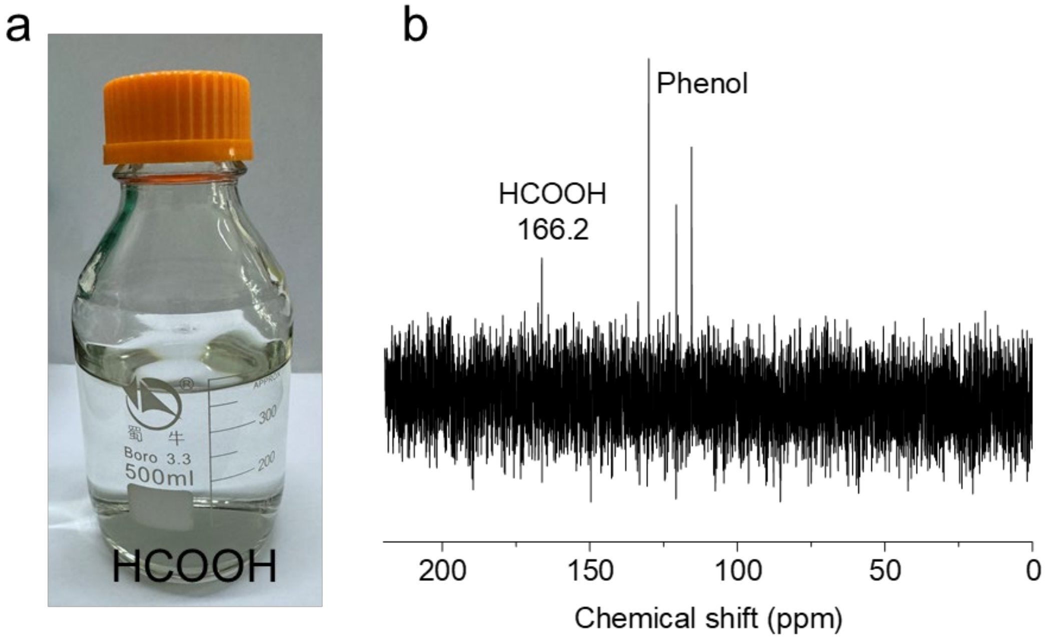

Supplementary Fig. 42 (a) Photograph of liquid HCOOH obtained by direct CO_2 electrolysis in acid electrolyte, (b) ^{13}C-NMR spectrum of liquid HCOOH. Source data for Supplementary Fig. 42b are provided as a Source Data file.

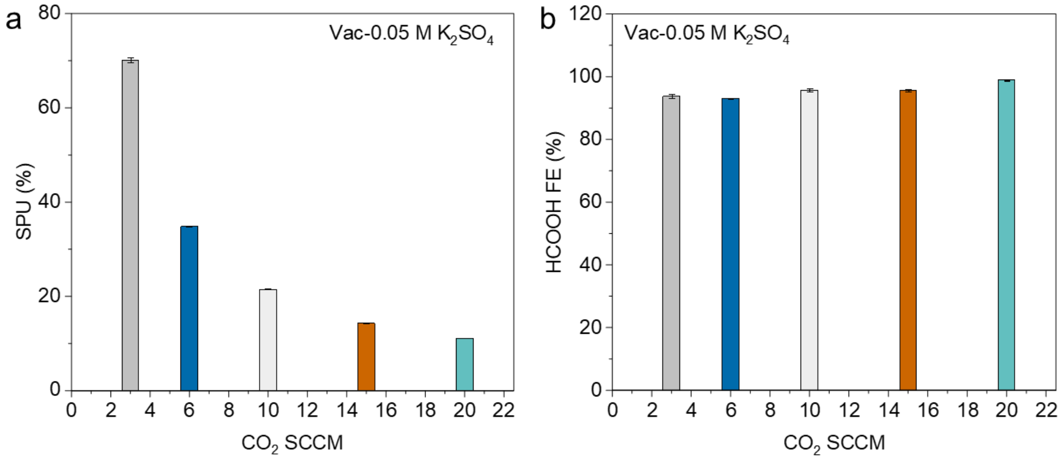

Supplementary Fig. 43 (a) SPU and (b) HCOOH FE of Vac under different CO_2 gas flow rate at 300mA cm^{-2}. Error bars represent the standard deviation of three independent measurements. Source data for Supplementary Fig. 43 are provided as a Source Data file.

Supplementary Table 1. In K-edge EXAFS Fitting Parameters

Sample	Path	CN	R(Å)	$\sigma^2(10^{-3}Å^2)$	ΔE_0(eV)	R factor
Vac	In-O	5.96	2.14	5.556	3.52	0.01
	In-O-In	1.17	3.97	2.63		
Vac after CO_2RR	In-O	6.48	2.07	2.77	6.87	0.01
	In-In	8.8	3.54	6.15		
	In-O-In	3.3	3.95	4.57		

Supplementary Table 2. HCOOH FE comparison of the recently reported electrocatalysts under different catalytic system

Sample	Electrolyte	Current density (mA cm^{-2})	HCOOH FE (%)	Stability (h)	Ref.
**Vac**	**0.05 M H_2SO_4 + 0.05 M K_2SO_4**	**300**	**98.9**	**> 100**	**This work**
Op-Ag_1In	0.5 M $NaHCO_3$	70	93.5	24	1
OD-PIn	0.5 M K HCO_3	200	92.1	< 24	2
PO_4^{3-}-In	1 M KOH	200	90.1	5	3
Sn SACs	H_2SO_4 (pH 3) + 0. 5 M K_2SO_4	100	90.8	18	4
Bi nanosheets	0.05 M H_2SO_4 + 3 M KCl	260	92.2	8	5
π-SnS	H_2SO_4 (pH 3) + 0. 5 M K_2SO_4	200	92.2	14	6
Vacancy Bi nanosheet	1 M KOH	200	95.0	30	7
Bi_4O_7/S	0.5 M K HCO_3	135	91.2	40	8
NU-1000-Sn	0.005 M H_2SO_4 + 3 M KCl	260	95	15	9
Bi nanosheets	0.05 M H_2SO_4 + 0.5 M K_2SO_4	200	> 80	12	10

Supplementary references

1. Fang, C. et al. Oxygen-pinned Ag_1In single-atom alloy for efficient electroreduction CO_2 to formate. *Adv. Energy Mater.* **14**, 2400813 (2024).
2. Liu, B. et al. Enhanced electrochemical CO_2 reduction to formate over phosphate-modified In: Water activation and active site tuning. *Angew. Chem. Int. Ed.* **63**, e202402070 (2024).
3. Zhang, B. et al. Enriching metal-oxygen species and phosphate modulating of active sites for robust electrocatalytical CO_2 reduction. *Adv. Mater.* **35**, 2304379 (2023).
4. Sun, B. et al. Unveiling ph-dependent adsorption strength of $*CO_2^-$ intermediate over high-density Sn single atom catalyst for acidic CO_2-to-HCOOH electroreduction. *Angew. Chem. Int. Ed.* **63**, e202318874 (2024).
5. Qiao, Y. et al. Engineering the local microenvironment over Bi nanosheets for highly selective electrocatalytic conversion of CO_2 to HCOOH in strong acid. *ACS Catal.* **12**, 2357-2364 (2022).
6. Shen, H. et al. Acidic CO_2-to-HCOOH electrolysis with industrial-level current on phase engineered tin sulfide. *Nat. Commun.* **14**, 2843 (2023).
7. Wang, X. et al. Steering geometric reconstruction of bismuth with accelerated dynamics for CO_2 electroreduction. *Angew. Chem. Int. Ed.* **63**, e202407665 (2024).
8. Liu, Y. et al. Promoting electrochemical CO_2 reduction to formate via sulfur-assisted electrolysis. *Adv. Funct. Mater.* **1**, 2403547 (2024).
9. Xue, H., Zhao, Z. H., Liao, P. Q., Chen, X. M. "Ship-in-a-bottle" integration of ditin(iv) sites into a metal-organic framework for boosting electroreduction of CO_2 in acidic electrolyte. *J. Am. Chem. Soc.* **145**, 16978-16982 (2023).
10. Chi, L. P. et al. Efficient and stable acidic CO_2 electrolysis to formic acid by a reservoir structure design. *Proc. Natl. Acad. Sci.* **120**, e2312876120 (2023).
