# Supplementary material for: Tip carbon encapsulation customizes cationic enrichment and valence stabilization for low K+ acidic CO2 electroreduction
Source: Nat Commun. 2025 Feb 19;16:1754. doi: 10.1038/s41467-025-56977-6 (PMC11839987; doi:10.1038/s41467-025-56977-6)
Supplement: Supplementary file 2 — Description of Additional Supplementary Information [file 41467_2025_56977_MOESM2_ESM.docx]

Supplementary data 1: Atomic coordinates of the optimized computational models for oxygen vacancy formation and CO_2_ reduction reaction calculations. The structures of the carbon layer/In_2_O_3_ complex and the In_2_O_3_ substrate with and without the oxygen vacancy, the CO_2_ reduction reaction intermediates on In (111) and In_2_O_3_ (110) surfaces, and the molecules involved in the reactions are given in the .vasp format.
