# Supplementary material for: Tip carbon encapsulation customizes cationic enrichment and valence stabilization for low K+ acidic CO2 electroreduction
Source: Nat Commun. 2025 Feb 19;16:1754. doi: 10.1038/s41467-025-56977-6 (PMC11839987; doi:10.1038/s41467-025-56977-6)
Supplement: Supplementary file 3 — Supplementary data 1 [file 41467_2025_56977_MOESM3_ESM.docx]

Atomic coordinates of the carbon layer/In_2_O_3_ complex model

1.00000000000000

20.4730999999999987 0.0000000000000000 0.0000000000000000

0.0000000000000000 14.4766999999999992 0.0000000000000000

0.0000000000000000 0.0000000000000000 30.0000000000000000

In O C

96 144 120

Direct

0.3330199999999977 0.2749999999999986 0.4833839255999948

0.2257131333009355 0.8988002034308593 0.3079549029659532

0.2254885014581802 0.3996684307231035 0.5448826847766810

0.3679482738503594 0.5250031646814222 0.3622337737321075

0.3668500066934826 0.0225695835748425 0.5997281121143345

0.4776031495288180 0.3985537938981736 0.3066701344241380

0.4755125958933419 0.8996254241209698 0.5449075677433584

0.2250000000000014 0.1499999999999986 0.4230677617333285

0.4750000000000014 0.6499999999999986 0.4230677617333285

0.1160436171017025 0.5254850508219704 0.3605634555777613

0.1202937813148210 0.0253716560179029 0.6002960901448162

0.2250000000000014 0.6499999999999986 0.4230677617333285

0.0830199999999977 0.2749999999999986 0.4833839255999948

0.4750000000000014 0.1499999999999986 0.4230677617333285

0.2236089831729586 0.3995909477992328 0.3074676005996153

0.2262724573231623 0.8981773770997350 0.5459855318453180

0.4754969016466859 0.8998365981574281 0.3060135400159112

0.4762754918892895 0.3981860337912906 0.5460049814512828

0.2290134224724341 0.1269754410526502 0.3032044008540495

0.2256996548390688 0.6325446334359199 0.5360690832603154

0.4749294048749633 0.1712936721623990 0.3124192373569556

0.4767059983233750 0.6657672788091702 0.5547431185589389

0.4750000000000014 0.4169800000000023 0.4312603666666632

0.2250000000000014 0.3830200000000019 0.4148751568000009

0.0982860907471093 0.7897741667919291 0.3513370325044107

0.1037236940711921 0.2967097166492820 0.5942910817616536

0.1000000000000014 0.0080200000000019 0.4915821303999977

0.0970456486505569 0.2583446939097129 0.3701264554682524

0.1107825124442456 0.7508011449183627 0.6030454336965292

0.1000000000000014 0.5419800000000023 0.4751913206666671

0.0830199999999977 0.7749999999999986 0.4833839255999948

0.1165214167079824 0.0244653718258476 0.3626325429314363

0.1168430932917559 0.5225783118220306 0.5997820474401507

0.3675200894608324 0.0248929863013032 0.3609911237750154

0.3702325898351688 0.5253747316043231 0.6002926274913278

0.3330199999999977 0.7749999999999986 0.4833839255999948

0.4800256567433365 0.6279809749940028 0.3014056566543672

0.4757904559884452 0.1325062293142381 0.5360834695596779

0.2233311594705967 0.6722588489567798 0.3138858756219278

0.2267712936209918 0.1657946830254104 0.5546992446966996

0.2250000000000014 0.9169800000000023 0.4312603666666632

0.4750000000000014 0.8830200000000019 0.4148751568000009

0.3494807598567107 0.2896759389712642 0.3513431532105784

0.3536712468816319 0.7967045155511059 0.5942786445715456

0.3500000000000014 0.5080200000000019 0.4915821303999977

0.3483151224404111 0.7587758348537434 0.3706027894256323

0.3607307640672559 0.2507704594742250 0.6030301803906610

0.3500000000000014 0.0419800000000023 0.4751913206666671

0.8330199999999977 0.2749999999999986 0.4833839255999948

0.7263917281510359 0.8985520226452124 0.3052384491506430

0.7255140788436591 0.3995721171685958 0.5449179625138640

0.8676617985988955 0.5247512098517930 0.3615902436384317

0.8668526389677851 0.0225662320472466 0.5997747560310458

0.9762752240190551 0.3986241510155750 0.3052036531744378

0.9754821644313978 0.8995729812741402 0.5448944881670741

0.7250000000000014 0.1499999999999986 0.4230677617333285

0.9750000000000014 0.6499999999999986 0.4230677617333285

0.6168642991313220 0.5247824611066108 0.3607972658808194

0.6202618412454655 0.0253486288872082 0.6003024716534142

0.7250000000000014 0.6499999999999986 0.4230677617333285

0.5830199999999977 0.2749999999999986 0.4833839255999948

0.9750000000000014 0.1499999999999986 0.4230677617333285

0.7246268245640621 0.3998887535748210 0.3052108832392619

0.7263162865098352 0.8981767228038109 0.5460232891814982

0.9736483610845355 0.8997278026050637 0.3051467713501168

0.9763673019609342 0.3982355245745595 0.5460052405500022

0.7281532638614863 0.1283731638438326 0.3000804839737867

0.7259626367227341 0.6323997216126216 0.5361826845444071

0.9741562983332486 0.1709686800477215 0.3109697166341476

0.9767906233388075 0.6657988500668850 0.5547079153173939

0.9750000000000014 0.4169800000000023 0.4312603666666632

0.7250000000000014 0.3830200000000019 0.4148751568000009

0.5999141346797572 0.7895878578410134 0.3508669114413578

0.6036824245792758 0.2966439395443530 0.5942751455645023

0.6000000000000014 0.0080200000000019 0.4915821303999977

0.5983501060569978 0.2584177374361687 0.3702321478825326

0.6107260251236326 0.7507546539036412 0.6030254281148331

0.6000000000000014 0.5419800000000023 0.4751913206666671

0.5830199999999977 0.7749999999999986 0.4833839255999948

0.6182540362854141 0.0248769179732520 0.3616469491472643

0.6168737108833966 0.5225339733772429 0.5997413870886490

0.8660751457796465 0.0248775464295292 0.3605098342115483

0.8703283274153719 0.5253777678814870 0.6003222699791806

0.8330199999999977 0.7749999999999986 0.4833839255999948

0.9776347045094553 0.6282035944360302 0.2999622265983177

0.9759511892082102 0.1324070823837076 0.5361836082352411

0.7243820471556859 0.6710340678598214 0.3110921102969328

0.7267112833824569 0.1657272751548149 0.5547334408307663

0.7250000000000014 0.9169800000000023 0.4312603666666632

0.9750000000000014 0.8830200000000019 0.4148751568000009

0.8497448399750240 0.2895835678052805 0.3507729304617513

0.8537035041583536 0.7967008444340351 0.5942884611206551

0.8500000000000014 0.5080200000000019 0.4915821303999977

0.8478811762671228 0.7582639007821612 0.3698841619597175

0.8607425805169697 0.2507916372163592 0.6030305797144635

0.8500000000000014 0.0419800000000023 0.4751913206666671

0.0222738149281128 0.2626188402092353 0.5460473628705032

0.1909287590103972 0.5347041025281669 0.3016287099010242

0.1806228091873834 0.0341482539295228 0.5415806254991011

0.1598801817586519 0.7838063441010945 0.2926340170139525

0.1611920386834882 0.2782203096659188 0.5328337287111964

0.2726400000000027 0.7861499999999992 0.4211302078666677

0.4273600000000002 0.0138499999999979 0.4250053155999964

0.1742256698381581 0.8942284348414233 0.3706101914136042

0.1785493935602176 0.4012640965424623 0.6099451612251912

0.0226400000000027 0.9040099999999995 0.4780080536000000

0.4032546479602175 0.1657376879684094 0.3674238711912210

0.3920503877126365 0.6639579369524498 0.6104960857334447

0.1750258811789465 0.1567878794960436 0.3600063665601851

0.1812067997382627 0.6467838467923173 0.6012541809934575

0.2950799999999987 0.6315699999999964 0.4779352553333283

0.2950799999999987 0.2637000000000000 0.4141751734666670

0.4049199999999971 0.5362999999999971 0.4319603499999971

0.4036641881512821 0.8825631273646337 0.3568022821850860

0.3995948441134374 0.3855504566586205 0.6046828309017300

0.2950799999999987 0.9184300000000007 0.4888381957333294

0.2932483640014354 0.7844340778304186 0.3110530890817813

0.2962235835326297 0.2877804308958005 0.5514595461760834

0.4114870907972161 0.5177945450808963 0.2941623132118067

0.4077861633888418 0.0133174439945236 0.5322423857252900

0.0226400000000027 0.6459899999999976 0.4887653974666648

0.2644121445076593 0.2648167849701082 0.3021267825097809

0.2722686841472211 0.7626255154074286 0.5460327379957732

0.4430346356494610 0.0342997715659763 0.2986064187954582

0.4306398163787293 0.5341306103506511 0.5416090812820258

0.2916888449097073 0.6266230095750700 0.3672558500745495

0.2963186328933531 0.1332819380895174 0.6069467968047206

0.0226400000000027 0.2861499999999992 0.4211302078666677

0.4089299999999980 0.3775499999999994 0.4869006418666615

0.1773600000000002 0.5138499999999979 0.4250053155999964

0.4260774373249703 0.3947989021466185 0.3696682240626288

0.4285380073232545 0.9012335048889000 0.6099228120224680

0.2726400000000027 0.4040099999999995 0.4780080536000000

0.1518832169093756 0.6666060826666751 0.3685640739476810

0.1420353303600521 0.1639152287595493 0.6105062408854067

0.0410699999999977 0.5322800000000001 0.4122376195999991

0.0450799999999987 0.1315700000000035 0.4779352553333283

0.0450799999999987 0.7637000000000000 0.4141751734666670

0.1549199999999971 0.0362999999999971 0.4319603499999971

0.2930294704847611 0.4195392672212250 0.3587864942996291

0.2941636935650678 0.9174562006913831 0.5990343387533130

0.1517878825409866 0.3822366793419789 0.3575924852771095

0.1496123769703885 0.8855723952525025 0.6046884065281404

0.0450799999999987 0.4184300000000007 0.4888381957333294

0.0445460123118409 0.2827864899373793 0.3086732085733672

0.0462433830818227 0.7878017929569410 0.5514576081103826

0.1601133928339067 0.0174478758907028 0.2949418521333911

0.1576206651040479 0.5134661977223161 0.5323710382981588

0.1589299999999980 0.2677199999999971 0.4338979038666650

0.1589299999999980 0.8775499999999994 0.4869006418666615

0.0415348448165318 0.9183779747398049 0.3579033798890805

0.0441272033841074 0.4174177918170017 0.5991383095435268

0.1589299999999980 0.6724499999999978 0.4798728091999962

0.0402903648106943 0.1260438925373109 0.3665470013809582

0.0462858168185960 0.6333339696855802 0.6069356650103401

0.2910699999999977 0.0322800000000001 0.4122376195999991

0.0434295545740113 0.5177465651632076 0.3091770467568793

0.0418548914557704 0.0173569252583476 0.5557746921270095

0.4089299999999980 0.7677199999999971 0.4338979038666650

0.2943678047692160 0.0167124632541018 0.3110281692882424

0.2918221535102035 0.5176210461029811 0.5557755809032585

0.4121148771931260 0.2838708511569408 0.2920276293425417

0.4111757697990086 0.7782062990622123 0.5327890409212159

0.4267078902591166 0.6572205768478128 0.3591882604673273

0.4311848864428072 0.1467250245231426 0.6012339865010858

0.4089299999999980 0.1724499999999978 0.4798728091999962

0.2726400000000027 0.1459899999999976 0.4887653974666648

0.0123498684287213 0.7655787737082420 0.2960702832025603

0.5226400000000027 0.6459899999999976 0.4887653974666648

0.7629770599165298 0.2655394068178865 0.2963862898838698

0.7722776292228986 0.7626297818079024 0.5460519745773240

0.9430538130256636 0.0341366742114175 0.2960154057198494

0.9306628087046036 0.5341430175705213 0.5416208386501040

0.7911037808886761 0.6260481117096219 0.3666391398436417

0.7963151111113049 0.1332811525796842 0.6069514768290603

0.5226400000000027 0.2861499999999992 0.4211302078666677

0.9089299999999980 0.3775499999999994 0.4869006418666615

0.6773600000000002 0.5138499999999979 0.4250053155999964

0.9262231558339664 0.3952736376803543 0.3690846350217782

0.9285131529637951 0.9011350846713597 0.6099220000534160

0.7726400000000027 0.4040099999999995 0.4780080536000000

0.6530349971679570 0.6649218276435676 0.3666443696801522

0.6420562149749571 0.1639083971938646 0.6104972489973690

0.5410699999999977 0.5322800000000001 0.4122376195999991

0.5450799999999987 0.1315700000000035 0.4779352553333283

0.5450799999999987 0.7637000000000000 0.4141751734666670

0.6549199999999971 0.0362999999999971 0.4319603499999971

0.7927771895768956 0.4181614237313198 0.3579359977927226

0.7941215946896918 0.9174646502733026 0.5991260775437226

0.6534658378001055 0.3829781992034782 0.3567578187416629

0.6495971229261883 0.8855110094468159 0.6046661018315334

0.5450799999999987 0.4184300000000007 0.4888381957333294

0.5453723728989246 0.2832321096931452 0.3092691908661109

0.5462280821013223 0.7877896592074265 0.5514481834877785

0.6600630737778204 0.0186106348436681 0.2919486624175534

0.6577917027189670 0.5133428660555239 0.5322632930816411

0.6589299999999980 0.2677199999999971 0.4338979038666650

0.6589299999999980 0.8775499999999994 0.4869006418666615

0.5432599055167415 0.9189409098265244 0.3584113198723458

0.5441751770532122 0.4174100405357990 0.5990140579592094

0.6589299999999980 0.6724499999999978 0.4798728091999962

0.5417597115501778 0.1261256165987632 0.3670268666457019

0.5463225284860158 0.6332596132929638 0.6069511723625141

0.7910699999999977 0.0322800000000001 0.4122376195999991

0.5453964653100414 0.5172647852896972 0.3093592831542011

0.5418700424258276 0.0174998911606323 0.5557632081653949

0.9089299999999980 0.7677199999999971 0.4338979038666650

0.7936189217241889 0.0175983875494268 0.3095104390077381

0.7918903199557192 0.5173198616274632 0.5557970536464580

0.9109771729641736 0.2838742912210606 0.2908502337775616

0.9111462521688196 0.7781769181294607 0.5327716165654977

0.9266578490988877 0.6567099468751820 0.3589459420370176

0.9312160949503649 0.1467117314350190 0.6012764561806847

0.9089299999999980 0.1724499999999978 0.4798728091999962

0.7726400000000027 0.1459899999999976 0.4887653974666648

0.5146993733880763 0.7653696261738716 0.2983974733872117

0.5222540055492146 0.2626222960482494 0.5460306730361850

0.6934044467495255 0.5340473683117182 0.2962000679709718

0.6806155911924634 0.0341135875595171 0.5416241275660667

0.6611937235144012 0.7838726938126612 0.2907940414634851

0.6611694152194758 0.2782163499119576 0.5327776176821928

0.7726400000000027 0.7861499999999992 0.4211302078666677

0.9273600000000002 0.0138499999999979 0.4250053155999964

0.6764059798917401 0.8952338756608000 0.3690502454784337

0.6785038096177516 0.4011242166051971 0.6099300685580218

0.5226400000000027 0.9040099999999995 0.4780080536000000

0.9027246089899919 0.1648817355281196 0.3665049551837782

0.8920463794893223 0.6639406939033492 0.6105016776638337

0.6768234008085088 0.1566046692730154 0.3589314535353623

0.6812277381092784 0.6467557748225019 0.6012424919026387

0.7950799999999987 0.6315699999999964 0.4779352553333283

0.7950799999999987 0.2637000000000000 0.4141751734666670

0.9049199999999971 0.5362999999999971 0.4319603499999971

0.9026932190222752 0.8829533123104980 0.3565840140087957

0.8996104904675732 0.3855707635453314 0.6046853556393188

0.7950799999999987 0.9184300000000007 0.4888381957333294

0.7946118225378385 0.7829144940327240 0.3089262541358195

0.7962263687622837 0.2877821597363663 0.5514641930550823

0.9096732809170547 0.5183141668362694 0.2920851072617197

0.9077044930181302 0.0134049975808423 0.5323422148250359

0.9357866152950081 0.0174005944770457 0.1354592476617071

0.8341074920500802 0.1007645643436916 0.1200298183633208

0.8687411412068471 0.0174083010509065 0.1228697387043098

0.7644867073275563 0.1006230616526906 0.1186020866842483

0.9338906869087822 0.1841415073966246 0.1400099682545815

0.8329761929098680 0.2673399320025851 0.1221347910450206

0.8675384140718094 0.1841553951671528 0.1257442862082669

0.7636894652813737 0.2672978372614949 0.1183958792232218

0.9320281911985546 0.3506048556356589 0.1441089171535026

0.8327531408596656 0.4338717211249303 0.1230555403206637

0.8664801290048274 0.3507501861181126 0.1283128287168296

0.7634856138021249 0.4340197514833832 0.1185344110380129

0.9322743876549779 0.5169563584568948 0.1437641363388167

0.8334193153718985 0.6004709068169731 0.1218817303779929

0.8667471399980116 0.5170289629394276 0.1281196699085143

0.7639947786852375 0.6007564137959065 0.1189668270550541

0.9345490554657658 0.6833832228962748 0.1390183052094898

0.8343593823990361 0.7669716711032066 0.1196777648561493

0.8680098933871889 0.6834076638312681 0.1250727531574114

0.7648359640158731 0.7672244075592758 0.1192541680288173

0.9361959351666087 0.8503065552009090 0.1347118563068221

0.8346802450368713 0.9338345111414910 0.1187841341739357

0.8689044359682399 0.8503773299938828 0.1224093040213603

0.7650084486788415 0.9338146543420791 0.1192323699966238

0.7304480244173419 0.0170129349491717 0.1223763551637802

0.6302668796364657 0.1003672979072689 0.1418332632423827

0.6635598161882328 0.0169201497289677 0.1353885298045512

0.5664321571953366 0.1005783721593474 0.1606750898632683

0.7295426196778934 0.1835636289556462 0.1205995415754160

0.6282173139324456 0.2670124420689390 0.1373747739937059

0.6617164042519974 0.1834326588531638 0.1314203021993805

0.5639873396972703 0.2671713008244652 0.1553680895299365

0.7288807194974609 0.3507100178419549 0.1198398858129295

0.6278304553142107 0.4341589828814207 0.1366888584984096

0.6605817633063893 0.3506975081878636 0.1290536019592565

0.5633716931401125 0.4341223655464006 0.1545317842914429

0.7291149360056579 0.5175647195180986 0.1204722884590410

0.6292390526404219 0.6010256864966763 0.1401511871959187

0.6611197270418501 0.5177120456306668 0.1303811906613035

0.5652532541893009 0.6008590999154393 0.1588179937778591

0.7299626678621678 0.6842149305732121 0.1219804196500647

0.6313122063545862 0.7674257395115234 0.1446838820188816

0.6626322093329122 0.6843107889988858 0.1340533277308538

0.5679948233688776 0.7672267188163460 0.1642521747611454

0.7307107989402297 0.8506901770748740 0.1230597676247811

0.6319993490229692 0.9338476452839117 0.1456945476843075

0.6640855979899811 0.8507395882197386 0.1367955614015131

0.5685391773325676 0.9338697379024268 0.1652580741866885

0.5360557958476448 0.0175444753420209 0.1731060588419666

0.4372217562907039 0.1010706898522216 0.1946974459988908

0.4713069491280777 0.0177051215296018 0.1902485037968802

0.3690538927801583 0.1010919308727267 0.2041075402502983

0.5335816939988618 0.1840178733607587 0.1673430732457817

0.4352505313681974 0.2675053946124137 0.1901814843994103

0.4690053876058968 0.1841885422842964 0.1848863433644610

0.3678805509386152 0.2675209480039555 0.2010926230430670

0.5318335322321914 0.3507689862320973 0.1638615114247912

0.4348464902881304 0.4338628602780688 0.1900800154520539

0.4675370206715286 0.3508352216606525 0.1818593918221367

0.3673617403082332 0.4337033268662864 0.2013807579802105

0.5324427099043234 0.5172671123444701 0.1659191641965236

0.4363143041368858 0.6003945323387683 0.1938565786248532

0.4682031896932841 0.5171410514508068 0.1839065184286852

0.3683535707539747 0.6003389502026195 0.2044127036857333

0.5352558330322239 0.6836559346053690 0.1715493395137315

0.4380894330568097 0.7670865427580651 0.1981989232944201

0.4704846041111921 0.6836278982959385 0.1891445713748610

0.3696793214868350 0.7670108610953150 0.2072040524326297

0.5371126614591972 0.8505789789341875 0.1753858674987896

0.4386808566862328 0.9342204090227568 0.1990030772203681

0.4720520155937180 0.8506213181916280 0.1926254547941753

0.3698278650066840 0.9341276410132764 0.2071335308984388

0.3350284983785556 0.0174787990943983 0.2074559647768695

0.2307047076958639 0.1004197925402082 0.2031224263089300

0.2657231052293838 0.0171943745901970 0.2056887866946866

0.1622112797777362 0.1002661396045852 0.1945675991306572

0.3342058345085235 0.1840963244988174 0.2048875441088924

0.2295499222010449 0.2670729894385602 0.2043332885054099

0.2647265229512215 0.1841059525433550 0.2053165180203038

0.1607455460279041 0.2669831683827866 0.1984584966321705

0.3332893809051996 0.3505732419252823 0.2040429846686536

0.2291426145301116 0.4339292701640574 0.2069762761031910

0.2639480858017151 0.3505800504434262 0.2065366121939647

0.1597875985715689 0.4338910561946889 0.2009613943823396

0.3334200140347932 0.5170951317199624 0.2059051449898135

0.2296374761962454 0.6008590137208287 0.2069264766144983

0.2641811440320581 0.5173631883718457 0.2081973189608419

0.1607853889701545 0.6009764064725474 0.1993804225504192

0.3345890034754220 0.6837327652412668 0.2085670722878561

0.2307114178571155 0.7674500375883113 0.2048040278466772

0.2647701020708837 0.6838640926439155 0.2084824715407763

0.1626384381942927 0.7674436302652410 0.1952728238075778

0.3353115639694479 0.8507525400492995 0.2091917311633082

0.2312710531015298 0.9338072728200757 0.2032230059684110

0.2655968383715006 0.8506299362377305 0.2070848063441630

0.1632609730344470 0.9337322389192195 0.1929558445988064

0.1297433082015331 0.0168367592935255 0.1867168018264849

0.0312728221592025 0.1005249403807795 0.1642901930258418

0.0644559725417295 0.0169741987521093 0.1708382029710762

0.9671393145622886 0.1007016595559236 0.1459996966465411

0.1278905030011543 0.1833415745680966 0.1909877206886819

0.0284847757448361 0.2669768781178519 0.1703013002536196

0.0618550403162885 0.1834194848065778 0.1761570123732881

0.9646924326830657 0.2671164698534980 0.1516062546240065

0.1262688999929011 0.3504892901111916 0.1951772844839468

0.0274001375970840 0.4337682371168938 0.1731339572038664

0.0597121438831394 0.3502698748348720 0.1811916876003892

0.9635825534222278 0.4336785480414420 0.1541185028159546

0.1263968738514336 0.5175853223795102 0.1952632754486103

0.0291036565141809 0.6007439011132121 0.1696517626532881

0.0601830437904383 0.5174182493267736 0.1808206329424635

0.9652030749766354 0.6004700098895626 0.1508643697609671

0.1283530175282763 0.6842202879804660 0.1911754073250549

0.0320513898837476 0.7669234670178966 0.1632301237369163

0.0626287208699736 0.6840865319882852 0.1754914722386545

0.9679322274970374 0.7669185104685354 0.1449173588207486

0.1300804163044589 0.8505775148693786 0.1867802327631907

0.0330344121913058 0.9336008623720105 0.1607580372211217

0.0648576295358723 0.8504610976257680 0.1703713195715935

0.9687162795730302 0.9337905494859413 0.1426203648218092

Atomic coordinates of the carbon layer/In_2_O_3_ complex model with an oxygen vacancy

1.00000000000000

20.4730999999999987 0.0000000000000000 0.0000000000000000

0.0000000000000000 14.4766999999999992 0.0000000000000000

0.0000000000000000 0.0000000000000000 30.0000000000000000

In O C

96 143 120

Direct

0.3330199999999977 0.2749999999999986 0.4833839255999948

0.2251016098787169 0.8990359676084595 0.3072487579160711

0.2249036201721775 0.4009163243673906 0.5446666558365454

0.3676677741246733 0.5249043659561263 0.3618406737935749

0.3566675223795867 0.0358998782246022 0.5980180768233366

0.4774681099642589 0.3984189533928971 0.3064806375465231

0.4745882951053628 0.8990969974962766 0.5395281665677643

0.2250000000000014 0.1499999999999986 0.4230677617333285

0.4750000000000014 0.6499999999999986 0.4230677617333285

0.1161611036566794 0.5257225338019667 0.3602196972311564

0.1201625931308671 0.0260115814421582 0.6005771904798789

0.2250000000000014 0.6499999999999986 0.4230677617333285

0.0830199999999977 0.2749999999999986 0.4833839255999948

0.4750000000000014 0.1499999999999986 0.4230677617333285

0.2236865109984928 0.3994601911894237 0.3072382341612740

0.2257614494982507 0.8996554843003468 0.5454001652889581

0.4748941617624706 0.8993764383535854 0.3055064494673485

0.4768674274905338 0.3990413306092497 0.5455825404994734

0.2284570023986126 0.1273645180891712 0.3025599090080558

0.2235691447442585 0.6303642487607072 0.5349556551908492

0.4749190317745672 0.1716059271261168 0.3123963007747173

0.4787125682734504 0.6640073290004652 0.5550826551898510

0.4750000000000014 0.4169800000000023 0.4312603666666632

0.2250000000000014 0.3830200000000019 0.4148751568000009

0.0984725758280236 0.7897791911042071 0.3513075894346366

0.1026251276480919 0.2982036074997123 0.5949312022111791

0.1000000000000014 0.0080200000000019 0.4915821303999977

0.0971858217029601 0.2583045369879307 0.3698003013460500

0.1124476652562384 0.7514882515053767 0.6026445263461705

0.1000000000000014 0.5419800000000023 0.4751913206666671

0.0830199999999977 0.7749999999999986 0.4833839255999948

0.1163811844008699 0.0245640162757223 0.3621340246084659

0.1171765694732147 0.5230537370887927 0.6009077504269229

0.3674717052852269 0.0252878062025681 0.3600604366037735

0.3691277042477450 0.5217836457278520 0.6013312928843331

0.3330199999999977 0.7749999999999986 0.4833839255999948

0.4793004346542543 0.6275836591576485 0.3008661153052493

0.4757252366736549 0.1337864037339363 0.5364574082968376

0.2232133963116366 0.6721811257055653 0.3133373775122641

0.2234510782192607 0.1700736905779077 0.5536673335869038

0.2250000000000014 0.9169800000000023 0.4312603666666632

0.4750000000000014 0.8830200000000019 0.4148751568000009

0.3494418216134267 0.2898195419915581 0.3512853441430654

0.3411334306737879 0.7739439072003214 0.5949663560341371

0.3500000000000014 0.5080200000000019 0.4915821303999977

0.3480281400624786 0.7585034719292669 0.3698031238201716

0.3571577176960600 0.2556458677328682 0.6045856718995463

0.3500000000000014 0.0419800000000023 0.4751913206666671

0.8330199999999977 0.2749999999999986 0.4833839255999948

0.7264483115133678 0.8985403082345655 0.3051956086939222

0.7257248821708283 0.3995324522898201 0.5447232168398156

0.8677364100433347 0.5246103312739732 0.3614216277666051

0.8670461217752070 0.0226059221741122 0.5999473003706075

0.9762218423260492 0.3985431409113533 0.3050048278749499

0.9752720547249449 0.8996650375370691 0.5447329915251715

0.7250000000000014 0.1499999999999986 0.4230677617333285

0.9750000000000014 0.6499999999999986 0.4230677617333285

0.6173314998235840 0.5248525199707562 0.3601259784601967

0.6200202502919010 0.0251453851535247 0.6006805689633379

0.7250000000000014 0.6499999999999986 0.4230677617333285

0.5830199999999977 0.2749999999999986 0.4833839255999948

0.9750000000000014 0.1499999999999986 0.4230677617333285

0.7248136520697885 0.3994117620649549 0.3052125240337652

0.7268891306599832 0.8986077060486320 0.5458719521678357

0.9738982946694140 0.8992084405736156 0.3050506640918509

0.9762812749757879 0.3983577299258014 0.5458945645181934

0.7281091405215748 0.1282765961778119 0.2999978400482548

0.7263894128281058 0.6321366958997369 0.5359367074702408

0.9741816091707007 0.1709091924891123 0.3109196967934488

0.9770258037977789 0.6658629136674982 0.5548722564251249

0.9750000000000014 0.4169800000000023 0.4312603666666632

0.7250000000000014 0.3830200000000019 0.4148751568000009

0.5997647356089877 0.7894905186567391 0.3502306006110363

0.6041545098151974 0.2970215698082667 0.5946135149361433

0.6000000000000014 0.0080200000000019 0.4915821303999977

0.5985092420860330 0.2583624401746208 0.3699989862504500

0.6119433157717654 0.7507374372117120 0.6032217349696926

0.6000000000000014 0.5419800000000023 0.4751913206666671

0.5830199999999977 0.7749999999999986 0.4833839255999948

0.6180409146047378 0.0247378314751650 0.3611929879611070

0.6179856378087033 0.5220377391464472 0.6000417343889123

0.8665921982108269 0.0248719009039272 0.3600690661251463

0.8704146026980562 0.5252309094695330 0.6004315954919524

0.8330199999999977 0.7749999999999986 0.4833839255999948

0.9776683514146421 0.6281457780563882 0.2998391693785082

0.9756675562688244 0.1327985209977594 0.5360582074286480

0.7244796690233108 0.6711007444007039 0.3110688913464598

0.7268691224840733 0.1657265110227446 0.5545649672075383

0.7250000000000014 0.9169800000000023 0.4312603666666632

0.9750000000000014 0.8830200000000019 0.4148751568000009

0.8498338980780051 0.2894895649814564 0.3507006212062013

0.8539093681615734 0.7965985742502352 0.5943655465726585

0.8500000000000014 0.5080200000000019 0.4915821303999977

0.8479952451112088 0.7581440927283698 0.3697925513340116

0.8607664749607387 0.2508472314958226 0.6031058500212180

0.8500000000000014 0.0419800000000023 0.4751913206666671

0.0222548815908464 0.2628599561506309 0.5461695058941941

0.1908715809607612 0.5346621050096262 0.3023286499681594

0.1823251947301899 0.0348827794838971 0.5422763585077688

0.1596803809241363 0.7836891950833689 0.2928753713925921

0.1599961591032043 0.2811832385052218 0.5329829575035308

0.2726400000000027 0.7861499999999992 0.4211302078666677

0.4273600000000002 0.0138499999999979 0.4250053155999964

0.1744975418535782 0.8946464699977266 0.3706027235798180

0.1790131564253775 0.4016464045043602 0.6100675092085882

0.0226400000000027 0.9040099999999995 0.4780080536000000

0.4032476373833117 0.1659686959382366 0.3680814157690623

0.3914942784457073 0.6593554708241709 0.6158944622755050

0.1750672369214894 0.1563996239429506 0.3600712993207350

0.1837448199464335 0.6480227681270651 0.6013269842749857

0.2950799999999987 0.6315699999999964 0.4779352553333283

0.2950799999999987 0.2637000000000000 0.4141751734666670

0.4049199999999971 0.5362999999999971 0.4319603499999971

0.4036528994300185 0.8824830514177642 0.3571404683710050

0.4011514553319589 0.3847806185984687 0.6057117247467285

0.2950799999999987 0.9184300000000007 0.4888381957333294

0.2929178039275854 0.7845802319590127 0.3109526268945746

0.2957499322632307 0.2892622721006010 0.5513074338551678

0.4110475861801980 0.5173627670468040 0.2943344023244954

0.4054760301766916 0.0095390995141109 0.5354339711652615

0.0226400000000027 0.6459899999999976 0.4887653974666648

0.2643542086660576 0.2645558692081128 0.3032650123237178

0.2687282068033835 0.7634984631602840 0.5448527304369198

0.4428854074767749 0.0345385319312120 0.2996024150716072

0.4294544615170010 0.5359348460448840 0.5426144934174445

0.2916938834609376 0.6269244942819138 0.3674556905913186

0.2889585114692247 0.1405028001223240 0.6089614541073347

0.0226400000000027 0.2861499999999992 0.4211302078666677

0.4089299999999980 0.3775499999999994 0.4869006418666615

0.1773600000000002 0.5138499999999979 0.4250053155999964

0.4260943400383204 0.3949936127724724 0.3698651607896576

0.2726400000000027 0.4040099999999995 0.4780080536000000

0.1523208925557850 0.6668539891056291 0.3688527620599774

0.1412030345671650 0.1650434442609147 0.6100744667021395

0.0410699999999977 0.5322800000000001 0.4122376195999991

0.0450799999999987 0.1315700000000035 0.4779352553333283

0.0450799999999987 0.7637000000000000 0.4141751734666670

0.1549199999999971 0.0362999999999971 0.4319603499999971

0.2928374570077731 0.4197098884763168 0.3591034725305595

0.3091031245558036 0.9098666336752812 0.6022318386030457

0.1519242594332617 0.3822700813702942 0.3576594485242138

0.1550904752936171 0.8863802781630462 0.6043022702443672

0.0450799999999987 0.4184300000000007 0.4888381957333294

0.0445121727429716 0.2826334941802209 0.3087045301867130

0.0471538722374237 0.7890868577180508 0.5518688236745499

0.1593705273246577 0.0178398670196709 0.2947723761010121

0.1563303225480439 0.5134379635280775 0.5329313540414654

0.1589299999999980 0.2677199999999971 0.4338979038666650

0.1589299999999980 0.8775499999999994 0.4869006418666615

0.0416266067741047 0.9182311949884182 0.3581247992357894

0.0437373513550862 0.4187375143758372 0.5996527870336688

0.1589299999999980 0.6724499999999978 0.4798728091999962

0.0403731383001958 0.1261976634405766 0.3668684685106018

0.0479247628862528 0.6349439593119430 0.6070883450051204

0.2910699999999977 0.0322800000000001 0.4122376195999991

0.0432474385741823 0.5176115419504127 0.3092481553845364

0.0424857633979746 0.0170752296335916 0.5558908597433347

0.4089299999999980 0.7677199999999971 0.4338979038666650

0.2934942268409193 0.0167983782954363 0.3112182968634429

0.2918337007696153 0.5186336146930560 0.5557424941862329

0.4121068413932405 0.2837475655040148 0.2924229126816518

0.4090859573775474 0.7814969502584527 0.5387700948464511

0.4266664644624712 0.6573165100979029 0.3592852959176009

0.4259880709979387 0.1465879675948638 0.6011273508514510

0.4089299999999980 0.1724499999999978 0.4798728091999962

0.2726400000000027 0.1459899999999976 0.4887653974666648

0.0128272345663334 0.7654229299960756 0.2963976700355334

0.5226400000000027 0.6459899999999976 0.4887653974666648

0.7633795976340407 0.2651207301844423 0.2969164359205737

0.7721302999792006 0.7627390685790455 0.5461308353128749

0.9428652199123133 0.0336882785560269 0.2966904366587446

0.9308118231728193 0.5342815167431088 0.5417331413768240

0.7910711152805399 0.6259806942160312 0.3668128200359081

0.7961578309083022 0.1332344076737079 0.6067750826059410

0.5226400000000027 0.2861499999999992 0.4211302078666677

0.9089299999999980 0.3775499999999994 0.4869006418666615

0.6773600000000002 0.5138499999999979 0.4250053155999964

0.9262530299671233 0.3952557847871749 0.3691073290939613

0.9285469182970794 0.9011435704018441 0.6098757665455774

0.7726400000000027 0.4040099999999995 0.4780080536000000

0.6528817316567591 0.6652406805261232 0.3667702511589856

0.6410653723431825 0.1633498264939687 0.6106058481113913

0.5410699999999977 0.5322800000000001 0.4122376195999991

0.5450799999999987 0.1315700000000035 0.4779352553333283

0.5450799999999987 0.7637000000000000 0.4141751734666670

0.6549199999999971 0.0362999999999971 0.4319603499999971

0.7928235728418814 0.4180599068698693 0.3580953157192766

0.7939865711124550 0.9175807790101993 0.5992293068800042

0.6534922498035796 0.3828213990873619 0.3568525558695415

0.6489650782451033 0.8854455219507855 0.6051271684917724

0.5450799999999987 0.4184300000000007 0.4888381957333294

0.5454368030946982 0.2830004357849008 0.3093721272145144

0.5436013953205535 0.7904350258099063 0.5526556984913784

0.6599156169211277 0.0185774787752520 0.2920823603593281

0.6576351522397534 0.5135141289358101 0.5324024472656608

0.6589299999999980 0.2677199999999971 0.4338979038666650

0.6589299999999980 0.8775499999999994 0.4869006418666615

0.5428537501162963 0.9185722972858968 0.3584779630580553

0.5444699510811083 0.4182607824043431 0.5987530441197280

0.6589299999999980 0.6724499999999978 0.4798728091999962

0.5416899646838444 0.1260661529930416 0.3673106037793833

0.5470482034299841 0.6343520136701615 0.6072056543882196

0.7910699999999977 0.0322800000000001 0.4122376195999991

0.5447389290073213 0.5171378859926880 0.3095896011913431

0.5373721513233198 0.0147675623817859 0.5580262723676385

0.9089299999999980 0.7677199999999971 0.4338979038666650

0.7932725348644597 0.0175158981229260 0.3098535156500857

0.7919584315230643 0.5173093515204092 0.5558860518475287

0.9110128581925611 0.2836898371067115 0.2909810478108322

0.9113262080220815 0.7781060105833756 0.5328984409748307

0.9266954563297909 0.6566744033619586 0.3589950526136830

0.9312173408144828 0.1467115578750209 0.6012793680997307

0.9089299999999980 0.1724499999999978 0.4798728091999962

0.7726400000000027 0.1459899999999976 0.4887653974666648

0.5145089335079739 0.7649834763500153 0.2991032042362026

0.5217295560844510 0.2624963166305447 0.5465793019324661

0.6932018122434029 0.5336755507637988 0.2971507496412694

0.6785717516923006 0.0340304909030288 0.5415474190458486

0.6614160950703573 0.7838008586859031 0.2907390418272142

0.6606785902063381 0.2779093448175915 0.5330016172214538

0.7726400000000027 0.7861499999999992 0.4211302078666677

0.9273600000000002 0.0138499999999979 0.4250053155999964

0.6760651634833895 0.8953062132952495 0.3691244084707836

0.6791262922435191 0.4007566672173429 0.6100099434889794

0.5226400000000027 0.9040099999999995 0.4780080536000000

0.9028438015776161 0.1649800321326818 0.3667437773054135

0.8925305105254762 0.6638981380260327 0.6104434066842248

0.6768080264060481 0.1565397011749071 0.3590192680313620

0.6815383867982429 0.6460962145866256 0.6012806567157417

0.7950799999999987 0.6315699999999964 0.4779352553333283

0.7950799999999987 0.2637000000000000 0.4141751734666670

0.9049199999999971 0.5362999999999971 0.4319603499999971

0.9030037241205334 0.8826594387136979 0.3567122483825381

0.8996779748696961 0.3854357210030715 0.6046527462880498

0.7950799999999987 0.9184300000000007 0.4888381957333294

0.7946072546833058 0.7829517065296859 0.3090463353953151

0.7961302157758723 0.2877616237784441 0.5515068385139462

0.9095539246558727 0.5182985303387021 0.2922589016668240

0.9077639767060828 0.0136136864919059 0.5324124319206703

0.9353483526759502 0.0173928753256575 0.1352910699517839

0.8335852753032756 0.1006941952298564 0.1198183792488922

0.8681710243671900 0.0173871819645274 0.1226725780624452

0.7639245896864610 0.1005652367347626 0.1184973271115744

0.9334292527079230 0.1842020222906615 0.1397532109955918

0.8325308104063665 0.2673304272365576 0.1219278924134778

0.8670252509040948 0.1841576123047027 0.1254951475435401

0.7631322223646120 0.2672882900764865 0.1181788081857752

0.9316469341452422 0.3505863922338918 0.1439040024791886

0.8322860367321253 0.4338338457958956 0.1227587565154770

0.8659969793890328 0.3506373016999473 0.1281348752664249

0.7629275460113376 0.4340067712000047 0.1182506800293848

0.9319736615942350 0.5169420060713622 0.1434620366099368

0.8330206112447748 0.6003134143029853 0.1216003745835538

0.8663352117670529 0.5169405858909820 0.1277758924409726

0.7635004581912588 0.6006553360423673 0.1187823705099951

0.9341913921812371 0.6833204065492935 0.1387817930843244

0.8339482016615470 0.7669066503448045 0.1196162539780501

0.8675983702875136 0.6833277697871872 0.1248760803228789

0.7643254626914706 0.7672149524288606 0.1192958372199849

0.9357560508531657 0.8501827752773904 0.1347055779869974

0.8342016904880326 0.9337880962971506 0.1186985012628003

0.8684453577974197 0.8502896123872714 0.1223417637910923

0.7645120432001145 0.9338158354823504 0.1191068193179630

0.7299633307575434 0.0169998474103374 0.1222559029790653

0.6297973024191948 0.1003490451449020 0.1418464646816667

0.6630176540922258 0.0169693479176033 0.1353507865669950

0.5660473486365597 0.1005769464750457 0.1608626186445671

0.7290344884052563 0.1836305870029719 0.1205125802948691

0.6277623639869564 0.2670090459031988 0.1373540651186605

0.6612118468826512 0.1834841672693686 0.1313795168604275

0.5634901950986595 0.2671642704707978 0.1554973719439745

0.7283514372121851 0.3506128094671615 0.1195701457032886

0.6273624694479200 0.4341314265210842 0.1366977395956700

0.6600410477555213 0.3506085205896659 0.1289302755124642

0.5629383052286511 0.4341170565535721 0.1546832942866466

0.7285971551033060 0.5175621830665668 0.1202338312102641

0.6288030915747039 0.6010012685511646 0.1402739585956416

0.6605437533668661 0.5177340964238138 0.1303415148901886

0.5648949417349037 0.6008427598876919 0.1590887238386487

0.7295327354366167 0.6842145408130287 0.1220030943729654

0.6309682410185989 0.7674135608954243 0.1448387187451100

0.6622296287751129 0.6843194553069267 0.1341876129983492

0.5676988604909841 0.7672103748851187 0.1646134295131823

0.7302314247507734 0.8506173115314616 0.1230083938836402

0.6314895237277977 0.9338956763258467 0.1456572506796189

0.6636063434570294 0.8506529677408800 0.1367931838531078

0.5681097302845065 0.9339360800334081 0.1653829835183012

0.5357689927010078 0.0176185842524432 0.1733571529856861

0.4369190590991163 0.1010144260941490 0.1951478878147120

0.4709889044724326 0.0177417586102397 0.1906933307616704

0.3686673073923271 0.1009648766478939 0.2044845054266063

0.5331810840426734 0.1840766948355704 0.1675957515327203

0.4348238077815528 0.2674456549997697 0.1904822999569258

0.4686312793991324 0.1842287154257448 0.1852354447852283

0.3673780318069937 0.2674819951528509 0.2015180779447779

0.5313363488500354 0.3506581310643783 0.1639788900364632

0.4344497597005993 0.4338160799916958 0.1903422503012711

0.4670577068579579 0.3506656195161598 0.1820549838001722

0.3669191096204687 0.4337311596013415 0.2016715923315436

0.5321358642744689 0.5172750057790594 0.1661387136893890

0.4359416889192688 0.6002031535957156 0.1942433337515088

0.4678366614146447 0.5171033797556915 0.1842116716968300

0.3678651891495189 0.6002163871258273 0.2046863470531476

0.5349971879878300 0.6836778802185222 0.1720089589801793

0.4378503544295401 0.7669992854256320 0.1986896190620515

0.4701889371120472 0.6835596453240964 0.1896592769985447

0.3693138366207464 0.7669740399776896 0.2075782220422419

0.5368641028302420 0.8505460547812689 0.1756782301131732

0.4384548919960897 0.9342400526660946 0.1994211143289409

0.4717838473433544 0.8505003192761966 0.1929974984256244

0.3695197036128530 0.9340630564308167 0.2074046140737552

0.3347262336193741 0.0173736173560158 0.2077665246451541

0.2302531840856784 0.1001599280773472 0.2033492700862523

0.2653006530422045 0.0170386183224056 0.2059736008961363

0.1618333849127051 0.1001235858284873 0.1943707541953761

0.3337861192856041 0.1840515334818601 0.2053351333625190

0.2291203688812803 0.2669078328310021 0.2047107310901792

0.2642778831519026 0.1839885409620452 0.2056864474734520

0.1602084868000959 0.2667623954274696 0.1986337817655864

0.3328658628993057 0.3504978111841046 0.2045127306569214

0.2285825126190657 0.4338110045591036 0.2076823518073573

0.2634386426409667 0.3503303698771229 0.2071330082491525

0.1591896190623480 0.4337897733282155 0.2015587728865378

0.3329368566197788 0.5170605759498582 0.2062045723014121

0.2291935435915276 0.6008290583892334 0.2073302902341767

0.2635975881477486 0.5172816996654011 0.2087584730798951

0.1603229726932821 0.6009544927645388 0.1995987303773025

0.3342455574389063 0.6837474583661983 0.2088706228724118

0.2304313767132285 0.7674074614410419 0.2047521348128625

0.2643884292417154 0.6839187104977102 0.2087114746744051

0.1623317644799268 0.7673819918493821 0.1949903543494862

0.3350072604268590 0.8506259719178851 0.2093696655876022

0.2308805671145883 0.9337299915993823 0.2032834555875391

0.2652575996470288 0.8504632462073094 0.2071151551868857

0.1629577981607453 0.9337033336641726 0.1927338026297857

0.1294603388218770 0.0168107007227041 0.1864143569876771

0.0308938892012914 0.1004407620561603 0.1639562985302147

0.0640441043675820 0.0169656885694280 0.1705127140934051

0.9666649192215206 0.1006758050894811 0.1457521482790453

0.1275169647204341 0.1832470895406742 0.1908042795883209

0.0281846583799441 0.2669340127493588 0.1700615546863835

0.0614607488563514 0.1833679635129483 0.1759146351471574

0.9642733542893689 0.2671392375727260 0.1513285919055433

0.1257589581965746 0.3502535345313122 0.1954369635280918

0.0271215093394796 0.4337653643896708 0.1729296741887980

0.0593719808899587 0.3501313906580877 0.1810701084414730

0.9632763670860365 0.4337376277984132 0.1538258773210289

0.1259783533155147 0.5175573900225914 0.1956482202887624

0.0287966143153108 0.6005820470414117 0.1695351150071792

0.0597975077092023 0.5173560481160525 0.1808310677220760

0.9648844822873679 0.6003697270760880 0.1505849503858818

0.1280034438426179 0.6842154040819557 0.1910314407569240

0.0317021344442947 0.7668463093558948 0.1630938796980317

0.0622616450608420 0.6840430632834880 0.1753480789856230

0.9674794461449121 0.7668133671748999 0.1447973422124543

0.1297953072674048 0.8504742997973236 0.1864764418257656

0.0326349953854322 0.9336046567537466 0.1605507920545279

0.0644738325024717 0.8503322816697979 0.1701301038824365

0.9681968063627339 0.9337864471464695 0.1425661894049003

Atomic coordinates of the In_2_O_3_ substrate model

1.00000000000000

20.4730999999999987 0.0000000000000000 0.0000000000000000

0.0000000000000000 14.4766999999999992 0.0000000000000000

0.0000000000000000 0.0000000000000000 30.0000000000000000

In O

96 144

Direct

0.3330199999999977 0.2749999999999986 0.4833839255999948

0.2259393847834517 0.8988610817532745 0.3061585145902299

0.2254624155889786 0.3996838829109919 0.5448662009193654

0.3675138818471524 0.5250427339290695 0.3616411360412357

0.3668096470774387 0.0225790223668696 0.5997811007559264

0.4763492223884072 0.3988025320404134 0.3059981094155617

0.4754709007905831 0.8996594213062679 0.5448684708268914

0.2250000000000014 0.1499999999999986 0.4230677617333285

0.4750000000000014 0.6499999999999986 0.4230677617333285

0.1164401349170987 0.5249691342384750 0.3605469531840981

0.1202298500770382 0.0254273437132255 0.6002747968236335

0.2250000000000014 0.6499999999999986 0.4230677617333285

0.0830199999999977 0.2749999999999986 0.4833839255999948

0.4750000000000014 0.1499999999999986 0.4230677617333285

0.2238941702585606 0.3994654898211962 0.3058956866275508

0.2263017456031520 0.8982320774408876 0.5459426072528046

0.4750588842599797 0.8995112565722323 0.3059738302184343

0.4763067859547689 0.3982253364324961 0.5459413661486749

0.2283406776065888 0.1280617031606894 0.3011165678192356

0.2257048616469746 0.6325616576701449 0.5360385409847268

0.4741329182604233 0.1710970088891983 0.3119945762044338

0.4767491570765969 0.6658143127887470 0.5546594272788639

0.4750000000000014 0.4169800000000023 0.4312603666666632

0.2250000000000014 0.3830200000000019 0.4148751568000009

0.0990850245998800 0.7898014262569362 0.3510225227563737

0.1036715413360625 0.2967069214383073 0.5942906671789885

0.1000000000000014 0.0080200000000019 0.4915821303999977

0.0975699976218820 0.2584180881558780 0.3701193884577449

0.1107158590195851 0.7508312165876276 0.6030360956252991

0.1000000000000014 0.5419800000000023 0.4751913206666671

0.0830199999999977 0.7749999999999986 0.4833839255999948

0.1174187776547129 0.0249315594028729 0.3616515991039932

0.1168117809458664 0.5226287899126021 0.5998113210170573

0.3666723398336771 0.0248837905176131 0.3606320959722566

0.3702380549613409 0.5253364365715711 0.6002781329884641

0.3330199999999977 0.7749999999999986 0.4833839255999948

0.4788116565398681 0.6278568687659671 0.3010451244735250

0.4757340167096160 0.1325277867702823 0.5360487462027024

0.2234254481583022 0.6713068923987464 0.3121486402629472

0.2267739956275179 0.1658343117919614 0.5546472117797094

0.2250000000000014 0.9169800000000023 0.4312603666666632

0.4750000000000014 0.8830200000000019 0.4148751568000009

0.3494957228608655 0.2897798381253693 0.3509549369654650

0.3536578468104281 0.7966188687325513 0.5942876694364827

0.3500000000000014 0.5080200000000019 0.4915821303999977

0.3479233403538515 0.7586166338451363 0.3700763607177393

0.3607110812610799 0.2507770460148961 0.6030313877638278

0.3500000000000014 0.0419800000000023 0.4751913206666671

0.8330199999999977 0.2749999999999986 0.4833839255999948

0.7262523919156934 0.8987897731742009 0.3057586077594294

0.7254524167719083 0.3996399107125370 0.5448748995255244

0.8675047463180547 0.5249073959655775 0.3617424711735708

0.8667904048857480 0.0225716414347730 0.5998087691898562

0.9760090415238807 0.3987460944761594 0.3058804036164844

0.9754530903207268 0.8996807384077385 0.5448656958801550

0.7250000000000014 0.1499999999999986 0.4230677617333285

0.9750000000000014 0.6499999999999986 0.4230677617333285

0.6167119490851860 0.5245734194935920 0.3607144999202490

0.6202429038762531 0.0253710071122484 0.6002821192773808

0.7250000000000014 0.6499999999999986 0.4230677617333285

0.5830199999999977 0.2749999999999986 0.4833839255999948

0.9750000000000014 0.1499999999999986 0.4230677617333285

0.7245967033728817 0.3992733993403164 0.3055888858479818

0.7263033942093173 0.8982512315114590 0.5459525065631752

0.9738235444946497 0.8996120545485979 0.3056097274894327

0.9763408865600240 0.3982719695279542 0.5459529027217087

0.7280311478035628 0.1279203422435131 0.3007524771366050

0.7257246237740986 0.6325317641291974 0.5360565267483750

0.9734996300422054 0.1710426495431037 0.3117168562933941

0.9767637715895958 0.6658261435543125 0.5546573263655361

0.9750000000000014 0.4169800000000023 0.4312603666666632

0.7250000000000014 0.3830200000000019 0.4148751568000009

0.5998078030132703 0.7894431008600051 0.3510522270337546

0.6036240941453315 0.2966329440263848 0.5942726085300407

0.6000000000000014 0.0080200000000019 0.4915821303999977

0.5979669074285852 0.2585307529617751 0.3700918610783930

0.6107268016993856 0.7507767085083117 0.6030390910926826

0.6000000000000014 0.5419800000000023 0.4751913206666671

0.5830199999999977 0.7749999999999986 0.4833839255999948

0.6177385278780250 0.0248820802311798 0.3617497884170220

0.6167717203354002 0.5225520218962458 0.5998002430488825

0.8663443673164366 0.0248851828821088 0.3604992335326216

0.8702120763917449 0.5253453686009593 0.6002930050237936

0.8330199999999977 0.7749999999999986 0.4833839255999948

0.9779853757506487 0.6280678779053872 0.3007515737430424

0.9757438964129364 0.1325815623142006 0.5360620735339268

0.7241671756935801 0.6710326127692525 0.3116110261111658

0.7267817535277672 0.1657575770959738 0.5546705156471319

0.7250000000000014 0.9169800000000023 0.4312603666666632

0.9750000000000014 0.8830200000000019 0.4148751568000009

0.8495045952744888 0.2897260504310779 0.3510409577905591

0.8536044936647684 0.7965982081049106 0.5942894515494856

0.8500000000000014 0.5080200000000019 0.4915821303999977

0.8477996849619345 0.7584413700691002 0.3700986722043638

0.8607269743800856 0.2507989761655196 0.6030400069852533

0.8500000000000014 0.0419800000000023 0.4751913206666671

0.0222451691794987 0.2626138134695495 0.5460231864481884

0.1925348286530735 0.5337791348773493 0.2983315527199508

0.1805914839329148 0.0341437393450050 0.5416546227845771

0.1602926831833642 0.7838356606304018 0.2918564869153997

0.1611862969169948 0.2782296656972960 0.5328347973810423

0.2726400000000027 0.7861499999999992 0.4211302078666677

0.4273600000000002 0.0138499999999979 0.4250053155999964

0.1754432066610647 0.8954683059659629 0.3697701188767439

0.1784939397849570 0.4011977910464282 0.6098799498244958

0.0226400000000027 0.9040099999999995 0.4780080536000000

0.4027512892631506 0.1653552227044557 0.3674160606707488

0.3920119587360915 0.6638958762942551 0.6105292142289329

0.1761315705161621 0.1569722087384164 0.3593842350441960

0.1812005271327308 0.6468370219659363 0.6012456536216746

0.2950799999999987 0.6315699999999964 0.4779352553333283

0.2950799999999987 0.2637000000000000 0.4141751734666670

0.4049199999999971 0.5362999999999971 0.4319603499999971

0.4033042711679045 0.8826865134411150 0.3569590662558184

0.3995340398438856 0.3855755369859537 0.6046872860487156

0.2950799999999987 0.9184300000000007 0.4888381957333294

0.2934046914716788 0.7835182570491170 0.3100925030717363

0.2962083883821904 0.2878836521810868 0.5514990419763564

0.4106159198015007 0.5186013257587447 0.2927444627622720

0.4076530491759485 0.0134437122711404 0.5323682366506759

0.0226400000000027 0.6459899999999976 0.4887653974666648

0.2630527975957619 0.2654928431479959 0.2981110315003477

0.2722452826975612 0.7626360729043782 0.5460340639183414

0.4431905428369731 0.0335294845049958 0.2982947662826732

0.4306180900389762 0.5341500681429139 0.5416498256363198

0.2911231856720065 0.6262022174086468 0.3669711245391269

0.2962870766358918 0.1333450970644918 0.6068764994035405

0.0226400000000027 0.2861499999999992 0.4211302078666677

0.4089299999999980 0.3775499999999994 0.4869006418666615

0.1773600000000002 0.5138499999999979 0.4250053155999964

0.4256312106996134 0.3956081552482331 0.3695754299005500

0.4284767401530374 0.9011553228819196 0.6098904905488283

0.2726400000000027 0.4040099999999995 0.4780080536000000

0.1523834688000960 0.6654863572522629 0.3674718935960968

0.1419786647101304 0.1638864676578322 0.6104913434289543

0.0410699999999977 0.5322800000000001 0.4122376195999991

0.0450799999999987 0.1315700000000035 0.4779352553333283

0.0450799999999987 0.7637000000000000 0.4141751734666670

0.1549199999999971 0.0362999999999971 0.4319603499999971

0.2925811211984950 0.4186096963280903 0.3582508401289246

0.2941723668651974 0.9173744889328361 0.5989643066318351

0.1527709081370929 0.3826755289901058 0.3571491431260867

0.1495461374598506 0.8856360010942907 0.6046784148225299

0.0450799999999987 0.4184300000000007 0.4888381957333294

0.0436494037110222 0.2833565935786098 0.3098106450091507

0.0461951998155286 0.7878766460539168 0.5515034606582603

0.1601822644492046 0.0184407163366913 0.2928619846359979

0.1576071531712557 0.5134949186170914 0.5323936279807100

0.1589299999999980 0.2677199999999971 0.4338979038666650

0.1589299999999980 0.8775499999999994 0.4869006418666615

0.0423462901673375 0.9185526254567975 0.3581037600136554

0.0441505380975542 0.4174698591850614 0.5990333972798823

0.1589299999999980 0.6724499999999978 0.4798728091999962

0.0409126611967565 0.1260853690983197 0.3668370551788342

0.0462773865600568 0.6333754582460003 0.6068772061355432

0.2910699999999977 0.0322800000000001 0.4122376195999991

0.0433499238609869 0.5176291183083706 0.3103158669089368

0.0417848869204391 0.0175602062094526 0.5557543719120375

0.4089299999999980 0.7677199999999971 0.4338979038666650

0.2935160056252215 0.0176491558220491 0.3105190289568398

0.2917982436361868 0.5176237142392850 0.5557754899680631

0.4110831127892354 0.2837622933819119 0.2918165049957224

0.4111809567971747 0.7782174036539899 0.5328372546750941

0.4263050859124959 0.6569146482359827 0.3592728420658915

0.4311863605705355 0.1467951661482658 0.6012378815166812

0.4089299999999980 0.1724499999999978 0.4798728091999962

0.2726400000000027 0.1459899999999976 0.4887653974666648

0.0126412435667902 0.7652547886251568 0.2977162501422015

0.5226400000000027 0.6459899999999976 0.4887653974666648

0.7629664845293078 0.2649887408655474 0.2976493907771456

0.7722278900261799 0.7626162971122170 0.5460341023048585

0.9421502201947405 0.0339070775589150 0.2976802358742461

0.9305855322554919 0.5341686724455172 0.5416542905806807

0.7911652825336262 0.6260745283257023 0.3669781751421723

0.7962992018089992 0.1333268591951082 0.6069049292004252

0.5226400000000027 0.2861499999999992 0.4211302078666677

0.9089299999999980 0.3775499999999994 0.4869006418666615

0.6773600000000002 0.5138499999999979 0.4250053155999964

0.9256473351062837 0.3954620579787687 0.3696013932170246

0.9284455530966298 0.9011691363485497 0.6098836696789860

0.7726400000000027 0.4040099999999995 0.4780080536000000

0.6528353221808629 0.6648822788820183 0.3671284422729428

0.6419454780835423 0.1638548780465072 0.6105309788072546

0.5410699999999977 0.5322800000000001 0.4122376195999991

0.5450799999999987 0.1315700000000035 0.4779352553333283

0.5450799999999987 0.7637000000000000 0.4141751734666670

0.6549199999999971 0.0362999999999971 0.4319603499999971

0.7927132401836872 0.4184338708955551 0.3582655532818606

0.7941302246938307 0.9174312772598157 0.5990104094766409

0.6533264091199200 0.3826171847203739 0.3569224398668425

0.6495705965557832 0.8855627443093610 0.6046774567179511

0.5450799999999987 0.4184300000000007 0.4888381957333294

0.5441258383644083 0.2835788164339732 0.3097727522538634

0.5462114128412310 0.7878274847392532 0.5514927499523736

0.6599291749007534 0.0184733260540639 0.2927029291003365

0.6576320119607502 0.5134887133310286 0.5323744346577186

0.6589299999999980 0.2677199999999971 0.4338979038666650

0.6589299999999980 0.8775499999999994 0.4869006418666615

0.5430390035442514 0.9185026805914518 0.3584584753856390

0.5441017157933459 0.4174086464949127 0.5989787941931084

0.6589299999999980 0.6724499999999978 0.4798728091999962

0.5414087781822374 0.1261609706301527 0.3671096638938565

0.5462941201715239 0.6333199630488693 0.6068978232195619

0.7910699999999977 0.0322800000000001 0.4122376195999991

0.5439822470285591 0.5174549928377559 0.3103695067225871

0.5418546417756502 0.0174557276590690 0.5557645805540687

0.9089299999999980 0.7677199999999971 0.4338979038666650

0.7934676783388568 0.0176617048685799 0.3100807074431629

0.7917980977039960 0.5174693607288106 0.5557703407377446

0.9105537461170954 0.2839933882687475 0.2916319312272790

0.9111732127850658 0.7781952269852823 0.5328273261763101

0.9263665499334124 0.6568769407622739 0.3593474251991751

0.9311830917185873 0.1467734087337078 0.6012578066342442

0.9089299999999980 0.1724499999999978 0.4798728091999962

0.7726400000000027 0.1459899999999976 0.4887653974666648

0.5136479094767665 0.7651866908230716 0.2982936994160639

0.5222558452836168 0.2626038677724196 0.5460353446376516

0.6932936728758605 0.5334735132605957 0.2977595437272740

0.6806207462324636 0.0341604682756653 0.5416328956110874

0.6609347040784854 0.7837457619065376 0.2916551614739973

0.6611599237793655 0.2781608535798057 0.5328437782582967

0.7726400000000027 0.7861499999999992 0.4211302078666677

0.9273600000000002 0.0138499999999979 0.4250053155999964

0.6759243859271251 0.8953811717330353 0.3695061140971205

0.6783959281257381 0.4011908401237463 0.6098938624826360

0.5226400000000027 0.9040099999999995 0.4780080536000000

0.9025462616610582 0.1652094199198189 0.3672929946022023

0.8919300085090924 0.6638519082496686 0.6105253011480514

0.6764198295829189 0.1568687764218026 0.3593162007432369

0.6811940583632321 0.6467921512827530 0.6012409933075986

0.7950799999999987 0.6315699999999964 0.4779352553333283

0.7950799999999987 0.2637000000000000 0.4141751734666670

0.9049199999999971 0.5362999999999971 0.4319603499999971

0.9028033953640460 0.8827040732875560 0.3570138794743727

0.8995691843687368 0.3855657024229870 0.6046930280778398

0.7950799999999987 0.9184300000000007 0.4888381957333294

0.7941177341331169 0.7835026952669963 0.3097330810701422

0.7962080369811915 0.2878539526025818 0.5514937157418061

0.9098957291593237 0.5185147932554699 0.2927556580796988

0.9076242632618161 0.0135296581445701 0.5323860698302312

Atomic coordinates of the In_2_O_3_ substrate model with an oxygen vacancy

1.00000000000000

20.4730999999999987 0.0000000000000000 0.0000000000000000

0.0000000000000000 14.4766999999999992 0.0000000000000000

0.0000000000000000 0.0000000000000000 30.0000000000000000

In O

96 143

Direct

0.3330199999999977 0.2749999999999986 0.4833839255999948

0.2254212605715551 0.8985216028655643 0.3053743754428592

0.2247729402777863 0.4009296824294132 0.5447327077741582

0.3674505142955338 0.5247376052755212 0.3615672775949439

0.3559149857074456 0.0361703943360255 0.5977511498528446

0.4762431007284786 0.3984221056365153 0.3060130853912961

0.4743657013149510 0.8991050697934641 0.5394465954156169

0.2250000000000014 0.1499999999999986 0.4230677617333285

0.4750000000000014 0.6499999999999986 0.4230677617333285

0.1164833094984759 0.5248921513630259 0.3603930116561586

0.1201611763883865 0.0259608379658479 0.6005756279014774

0.2250000000000014 0.6499999999999986 0.4230677617333285

0.0830199999999977 0.2749999999999986 0.4833839255999948

0.4750000000000014 0.1499999999999986 0.4230677617333285

0.2240936845138790 0.3991980392820169 0.3057410200740633

0.2256811965957935 0.8994789377092345 0.5454293311508811

0.4745880002391732 0.8992360912063907 0.3056614130086587

0.4769208952826092 0.3990932700167220 0.5455938957391749

0.2280127790981817 0.1278280645058051 0.3006780695100525

0.2234898266229342 0.6302085936844596 0.5349697781873436

0.4737462944671897 0.1712753659653820 0.3120492617254757

0.4787763869305226 0.6640407914085068 0.5550168336698399

0.4750000000000014 0.4169800000000023 0.4312603666666632

0.2250000000000014 0.3830200000000019 0.4148751568000009

0.0991898412817365 0.7895044220565496 0.3508975603361003

0.1023297328166862 0.2982576589839318 0.5949702266955809

0.1000000000000014 0.0080200000000019 0.4915821303999977

0.0977170576032605 0.2582453195633295 0.3699504627152570

0.1123162477194530 0.7514779037530982 0.6027426249893189

0.1000000000000014 0.5419800000000023 0.4751913206666671

0.0830199999999977 0.7749999999999986 0.4833839255999948

0.1173508006580038 0.0246773713544895 0.3615283431639292

0.1170172455883046 0.5229770739456915 0.6009784500387805

0.3664120754446240 0.0248992236950257 0.3596590557704327

0.3689590919344717 0.5218200264885308 0.6013630936697072

0.3330199999999977 0.7749999999999986 0.4833839255999948

0.4785084653781493 0.6276001663966573 0.3009895591010958

0.4756655695334626 0.1338518215583073 0.5364965081066011

0.2234975409466175 0.6710428510736461 0.3119023869862497

0.2232310387930213 0.1701944288849374 0.5535725412187256

0.2250000000000014 0.9169800000000023 0.4312603666666632

0.4750000000000014 0.8830200000000019 0.4148751568000009

0.3493161760679567 0.2896096806418333 0.3508697185410344

0.3409120646698395 0.7736173766463327 0.5950882474354721

0.3500000000000014 0.5080200000000019 0.4915821303999977

0.3474073640438645 0.7585360720537608 0.3694023074227268

0.3568939435463486 0.2560686146299299 0.6046439201459066

0.3500000000000014 0.0419800000000023 0.4751913206666671

0.8330199999999977 0.2749999999999986 0.4833839255999948

0.7262171106958529 0.8986258874765821 0.3058321541473961

0.7256204600063997 0.3994902840631467 0.5446874065193550

0.8675343000163611 0.5248180927298837 0.3616703110784095

0.8670229722036775 0.0226441819010034 0.5999552881949923

0.9761117944010917 0.3985808235591909 0.3058656013285839

0.9751241223345535 0.8996444054329811 0.5447148279934895

0.7250000000000014 0.1499999999999986 0.4230677617333285

0.9750000000000014 0.6499999999999986 0.4230677617333285

0.6170133396205831 0.5247537680884244 0.3601469724763799

0.6198129322500838 0.0250620750984114 0.6007067481807116

0.7250000000000014 0.6499999999999986 0.4230677617333285

0.5830199999999977 0.2749999999999986 0.4833839255999948

0.9750000000000014 0.1499999999999986 0.4230677617333285

0.7246550553930340 0.3992411163636541 0.3055779472035802

0.7268074220701916 0.8986118987519492 0.5458473035469722

0.9740489219258534 0.8991685306476775 0.3055860082351710

0.9761787571446886 0.3983646364835268 0.5458742378984346

0.7283907710801419 0.1275315419136920 0.3007662610884321

0.7262672284207942 0.6322241979089870 0.5359105494834054

0.9736785200125854 0.1710327768385399 0.3117462398425458

0.9769731985706105 0.6658721739182336 0.5548575419754609

0.9750000000000014 0.4169800000000023 0.4312603666666632

0.7250000000000014 0.3830200000000019 0.4148751568000009

0.5997098112843167 0.7895305354198999 0.3507034204250654

0.6039765444063876 0.2971203928647931 0.5947073859125903

0.6000000000000014 0.0080200000000019 0.4915821303999977

0.5981492549476295 0.2582718220196147 0.3698927735529144

0.6118397349113583 0.7507509848928131 0.6032588465432068

0.6000000000000014 0.5419800000000023 0.4751913206666671

0.5830199999999977 0.7749999999999986 0.4833839255999948

0.6175622685146755 0.0247934016846194 0.3615032711376757

0.6178785929023153 0.5220429981178913 0.6000681794516963

0.8666887641068716 0.0248528784196783 0.3603628912492473

0.8702724955409024 0.5252794757791244 0.6003876674717386

0.8330199999999977 0.7749999999999986 0.4833839255999948

0.9781625451449596 0.6274982449717154 0.3006934066301496

0.9756561184262630 0.1328244354403991 0.5360540280831201

0.7241947338357917 0.6712423219292851 0.3116878878714336

0.7267688376065248 0.1657022432265401 0.5545694914488095

0.7250000000000014 0.9169800000000023 0.4312603666666632

0.9750000000000014 0.8830200000000019 0.4148751568000009

0.8495144769251719 0.2896771286983734 0.3509653589517973

0.8537244548362208 0.7965834040161384 0.5944039083884513

0.8500000000000014 0.5080200000000019 0.4915821303999977

0.8480235868491274 0.7582349216134215 0.3699329880216370

0.8606488599213260 0.2508699998114352 0.6030682075332420

0.8500000000000014 0.0419800000000023 0.4751913206666671

0.0222470392847452 0.2628684577871143 0.5461403867946285

0.1925660786469479 0.5337434135285701 0.2985847147453746

0.1824311292345915 0.0348527260338028 0.5423416795203927

0.1604163653906830 0.7835614370358259 0.2917211355649563

0.1599968089730951 0.2813112084893726 0.5329012289698341

0.2726400000000027 0.7861499999999992 0.4211302078666677

0.4273600000000002 0.0138499999999979 0.4250053155999964

0.1757987667673003 0.8954107533554631 0.3695820924409173

0.1786612250819076 0.4015241719434936 0.6100972192200159

0.0226400000000027 0.9040099999999995 0.4780080536000000

0.4025140348552356 0.1651348101695324 0.3677985242171071

0.3910254536787821 0.6590313897891003 0.6161482906159137

0.1764635613512642 0.1564973429696897 0.3594347940307814

0.1836231175752001 0.6479963387082398 0.6013411708169071

0.2950799999999987 0.6315699999999964 0.4779352553333283

0.2950799999999987 0.2637000000000000 0.4141751734666670

0.4049199999999971 0.5362999999999971 0.4319603499999971

0.4032154265676053 0.8825448275697981 0.3573351231384678

0.4009438270361420 0.3850342832534816 0.6060088078683319

0.2950799999999987 0.9184300000000007 0.4888381957333294

0.2935018220954014 0.7832332172661352 0.3094448959845185

0.2957083894883362 0.2894192812548707 0.5512727976521791

0.4102314139720358 0.5182718168800857 0.2931439354950868

0.4053956231135123 0.0096726625219481 0.5354504511217295

0.0226400000000027 0.6459899999999976 0.4887653974666648

0.2632304047339745 0.2649635581302777 0.2981912157721654

0.2685065455502150 0.7633793180766585 0.5448739631576325

0.4426306060378362 0.0336429592483035 0.2986117641989329

0.4293536377034085 0.5360486060621907 0.5426721184397968

0.2912609498798092 0.6263837961121074 0.3672205269960441

0.2886071948750107 0.1409899807060982 0.6091195081701265

0.0226400000000027 0.2861499999999992 0.4211302078666677

0.4089299999999980 0.3775499999999994 0.4869006418666615

0.1773600000000002 0.5138499999999979 0.4250053155999964

0.4256783593732006 0.3953333042910863 0.3697622870590693

0.2726400000000027 0.4040099999999995 0.4780080536000000

0.1526742503346935 0.6656456342873804 0.3677897084956686

0.1409297048304618 0.1650554744324593 0.6098450439989671

0.0410699999999977 0.5322800000000001 0.4122376195999991

0.0450799999999987 0.1315700000000035 0.4779352553333283

0.0450799999999987 0.7637000000000000 0.4141751734666670

0.1549199999999971 0.0362999999999971 0.4319603499999971

0.2925884135703929 0.4185681475040939 0.3583705609131934

0.3090937023651754 0.9098055687272760 0.6022467952319412

0.1529646917671793 0.3825035941723328 0.3571546521056406

0.1550441100243909 0.8863470300795756 0.6043165170579822

0.0450799999999987 0.4184300000000007 0.4888381957333294

0.0438742818031064 0.2831523438208482 0.3098156647587803

0.0471842323400874 0.7891333618011463 0.5518881225860445

0.1598174482264175 0.0185722142784599 0.2926696374586633

0.1562291877128658 0.5134371857962869 0.5329283887228337

0.1589299999999980 0.2677199999999971 0.4338979038666650

0.1589299999999980 0.8775499999999994 0.4869006418666615

0.0425473499908743 0.9182445919560714 0.3582132044411331

0.0435383971404632 0.4187659240401519 0.5995715773729474

0.1589299999999980 0.6724499999999978 0.4798728091999962

0.0411686465402067 0.1260139031091453 0.3670937427435007

0.0478655693062645 0.6348633039265680 0.6070866161416737

0.2910699999999977 0.0322800000000001 0.4122376195999991

0.0434988567116097 0.5172926915809939 0.3101508938837590

0.0424847391668995 0.0170562920140999 0.5558758143571816

0.4089299999999980 0.7677199999999971 0.4338979038666650

0.2931127808496350 0.0170000257453445 0.3099330204556024

0.2917459541014886 0.5186322002816480 0.5557774398579168

0.4107516190156016 0.2837197389758286 0.2918870976660196

0.4088724843665080 0.7813168085534048 0.5389294052894007

0.4263678335310703 0.6574422347345910 0.3595040944017954

0.4253855626069463 0.1467786542214067 0.6010140428244940

0.4089299999999980 0.1724499999999978 0.4798728091999962

0.2726400000000027 0.1459899999999976 0.4887653974666648

0.0128682172461132 0.7649100126553010 0.2978073629587543

0.5226400000000027 0.6459899999999976 0.4887653974666648

0.7631422047319262 0.2648929950975691 0.2980655259335778

0.7720026294492791 0.7628212457828160 0.5461297236743839

0.9423748933516317 0.0335748903722904 0.2982642431163089

0.9307490520743540 0.5343341470525195 0.5417341898139465

0.7911369672402732 0.6261553115933708 0.3671416805733967

0.7961150937653612 0.1332341549340174 0.6067410238809192

0.5226400000000027 0.2861499999999992 0.4211302078666677

0.9089299999999980 0.3775499999999994 0.4869006418666615

0.6773600000000002 0.5138499999999979 0.4250053155999964

0.9257819739340306 0.3953824600182296 0.3696119786942521

0.9284242420188710 0.9011624520528250 0.6098708571759551

0.7726400000000027 0.4040099999999995 0.4780080536000000

0.6526795387914556 0.6653041289770256 0.3673484296536269

0.6409060584732603 0.1632686966984457 0.6105618509247928

0.5410699999999977 0.5322800000000001 0.4122376195999991

0.5450799999999987 0.1315700000000035 0.4779352553333283

0.5450799999999987 0.7637000000000000 0.4141751734666670

0.6549199999999971 0.0362999999999971 0.4319603499999971

0.7926248814378528 0.4185251360058948 0.3583538110708590

0.7938955758725978 0.9176199225088679 0.5991515940878238

0.6532214013616193 0.3824991882741877 0.3569215288373561

0.6487163413015767 0.8854784671960920 0.6052148983617218

0.5450799999999987 0.4184300000000007 0.4888381957333294

0.5438462845356805 0.2830140688378411 0.3099006980614559

0.5434725160622863 0.7905191579344565 0.5526741885127372

0.6599701009276624 0.0183102303449342 0.2927999747529770

0.6574981568847837 0.5135718520332009 0.5324240451013083

0.6589299999999980 0.2677199999999971 0.4338979038666650

0.6589299999999980 0.8775499999999994 0.4869006418666615

0.5425582255644938 0.9183028872901122 0.3585747582048929

0.5442345802121253 0.4183060846224977 0.5988723204848577

0.6589299999999980 0.6724499999999978 0.4798728091999962

0.5410907437608604 0.1260645147648934 0.3672767545910128

0.5468530825198864 0.6343107554078762 0.6071456887691458

0.7910699999999977 0.0322800000000001 0.4122376195999991

0.5436133803643952 0.5171600142530800 0.3102682913521301

0.5371055382468228 0.0147369890518212 0.5580309694354949

0.9089299999999980 0.7677199999999971 0.4338979038666650

0.7934979528863710 0.0171579792478775 0.3102098398139141

0.7917744955498568 0.5172418956637456 0.5558380133423607

0.9107719700225605 0.2838293290224266 0.2917251477657218

0.9113414645461495 0.7780879427632406 0.5329068023559670

0.9264661313320131 0.6567771461762580 0.3592704378345182

0.9311610496395838 0.1467125841388703 0.6012489248875947

0.9089299999999980 0.1724499999999978 0.4798728091999962

0.7726400000000027 0.1459899999999976 0.4887653974666648

0.5134002167189832 0.7650147583099652 0.2983281929250444

0.5215639109806443 0.2625732051642147 0.5466129958884337

0.6927994959406332 0.5336676834226225 0.2985199627487896

0.6783823844743966 0.0339975676663767 0.5416058028731616

0.6609466412577768 0.7837339362219619 0.2916586700516366

0.6604576846586947 0.2778584903855337 0.5330527253391056

0.7726400000000027 0.7861499999999992 0.4211302078666677

0.9273600000000002 0.0138499999999979 0.4250053155999964

0.6756058960837890 0.8954243859389188 0.3696221699701837

0.6788502150749497 0.4006946214092953 0.6100052202698947

0.5226400000000027 0.9040099999999995 0.4780080536000000

0.9026837371145964 0.1653156119729109 0.3673507092111924

0.8923442731139851 0.6638658339313536 0.6103682681541373

0.6763699520202486 0.1565153102702096 0.3593379989257922

0.6813394918246750 0.6462076076015376 0.6011974267758923

0.7950799999999987 0.6315699999999964 0.4779352553333283

0.7950799999999987 0.2637000000000000 0.4141751734666670

0.9049199999999971 0.5362999999999971 0.4319603499999971

0.9030306640645336 0.8825262537963749 0.3570683664916467

0.8996038173314118 0.3854044721925409 0.6045904142909360

0.7950799999999987 0.9184300000000007 0.4888381957333294

0.7939673437945732 0.7833053297109523 0.3097714138421085

0.7959207743978843 0.2877444119308364 0.5515175880032213

0.9099418436569031 0.5182936253869695 0.2927908568238998

0.9077264168120571 0.0135903415998281 0.5324566892699863

Atomic coordinates of the In (111) surface model

1.00000000000000

13.6434999999999995 0.0000000000000000 0.0000000000000000

-4.5478420000000002 11.0855879999999996 0.0000000000000000

0.0000000000000000 0.0000000000000000 27.2580999999999989

In

48

Direct

0.0825200000000024 0.2475499999999968 0.4048999999999978

0.2772300000000030 0.3316999999999979 0.4809800000000024

0.1562025305895407 0.4574476397538308 0.5562141105326464

0.0316333481430325 0.0736637221575334 0.6295213146022833

0.3184899999999971 0.4554699999999983 0.3668600000000026

0.1798700000000011 0.0396199999999993 0.4429400000000001

0.0517470814808432 0.1518507486052690 0.5185260179006785

0.2617693271452796 0.2781594947159045 0.6032103829544989

0.4158499999999989 0.2475499999999968 0.4048999999999978

0.6105700000000027 0.3316999999999979 0.4809800000000024

0.4866880791298898 0.4562175248968293 0.5564394885255178

0.3635970606757253 0.0799047645772129 0.6300161159337364

0.6518200000000007 0.4554699999999983 0.3668600000000026

0.5132100000000008 0.0396199999999993 0.4429400000000001

0.3844831083701011 0.1514057114431780 0.5188360479260403

0.5952349193437951 0.2741493361309966 0.6032192897292111

0.7491800000000026 0.2475499999999968 0.4048999999999978

0.9438999999999993 0.3316999999999979 0.4809800000000024

0.8235809231076009 0.4509677606465363 0.5565911719570518

0.6954202789537435 0.0737402657497290 0.6284455247194664

0.9851600000000005 0.4554699999999983 0.3668600000000026

0.8465399999999974 0.0396199999999993 0.4429400000000001

0.7184049610143524 0.1505053002307730 0.5185473686877815

0.9311526087927806 0.2716718249085741 0.6036507453258404

0.0825200000000024 0.7475499999999968 0.4048999999999978

0.2772300000000030 0.8316999999999979 0.4809800000000024

0.1562025305895407 0.9574476397538310 0.5562141105326464

0.0316333481430325 0.5736637221575333 0.6295213146022833

0.3184899999999971 0.9554699999999983 0.3668600000000026

0.1798700000000011 0.5396199999999993 0.4429400000000001

0.0517470814808432 0.6518507486052693 0.5185260179006785

0.2617693271452796 0.7781594947159048 0.6032103829544989

0.4158499999999989 0.7475499999999968 0.4048999999999978

0.6105700000000027 0.8316999999999979 0.4809800000000024

0.4866880791298898 0.9562175248968295 0.5564394885255178

0.3635970606757253 0.5799047645772129 0.6300161159337364

0.6518200000000007 0.9554699999999983 0.3668600000000026

0.5132100000000008 0.5396199999999993 0.4429400000000001

0.3844831083701012 0.6514057114431778 0.5188360479260404

0.5952349193437950 0.7741493361309967 0.6032192897292111

0.7491800000000026 0.7475499999999968 0.4048999999999978

0.9438999999999993 0.8316999999999979 0.4809800000000024

0.8235809231076009 0.9509677606465365 0.5565911719570518

0.6954202789537435 0.5737402657497289 0.6284455247194664

0.9851600000000005 0.9554699999999983 0.3668600000000026

0.8465399999999974 0.5396199999999993 0.4429400000000001

0.7184049610143524 0.6505053002307729 0.5185473686877815

0.9311526087927806 0.7716718249085746 0.6036507453258404

Atomic coordinates of H* adsorption on the In (111) surface model

1.00000000000000

13.6434999999999995 0.0000000000000000 0.0000000000000000

-4.5478420000000002 11.0855879999999996 0.0000000000000000

0.0000000000000000 0.0000000000000000 27.2580999999999989

In H

48 1

Direct

0.0825200000000024 0.2475499999999968 0.4048999999999978

0.2772300000000030 0.3316999999999979 0.4809800000000024

0.1562000466022511 0.4561868390633137 0.5560915238247475

0.0365363650458264 0.0785414098564274 0.6306705827483086

0.3184899999999971 0.4554699999999983 0.3668600000000026

0.1798700000000011 0.0396199999999993 0.4429400000000001

0.0528536208051236 0.1514377167243381 0.5189159235589997

0.2640717846673581 0.2758281113953274 0.6033151040694094

0.4158499999999989 0.2475499999999968 0.4048999999999978

0.6105700000000027 0.3316999999999979 0.4809800000000024

0.4810644613202584 0.4549237638857733 0.5541169304756650

0.3562485457752089 0.0719219820643886 0.6297242277755686

0.6518200000000007 0.4554699999999983 0.3668600000000026

0.5132100000000008 0.0396199999999993 0.4429400000000001

0.3812860828104192 0.1484475009722672 0.5183732213801662

0.5848108847961697 0.2629173397641296 0.5991586881026478

0.7491800000000026 0.2475499999999968 0.4048999999999978

0.9438999999999993 0.3316999999999979 0.4809800000000024

0.8258917348881241 0.4507490162861671 0.5560971424310779

0.6990195698798374 0.0843365437785906 0.6301795884661558

0.9851600000000005 0.4554699999999983 0.3668600000000026

0.8465399999999974 0.0396199999999993 0.4429400000000001

0.7192622142074256 0.1493388599199221 0.5184565194497139

0.9326951431809647 0.2735026165538343 0.6033125696514574

0.0825200000000024 0.7475499999999968 0.4048999999999978

0.2772300000000030 0.8316999999999979 0.4809800000000024

0.1518913753789235 0.9522546498836704 0.5556439955567996

0.0361397010123454 0.5785494478687880 0.6296571011205288

0.3184899999999971 0.9554699999999983 0.3668600000000026

0.1798700000000011 0.5396199999999993 0.4429400000000001

0.0524766096406779 0.6511266130786311 0.5182941652560658

0.2637370639375050 0.7791112490545373 0.6037568160677864

0.4158499999999989 0.7475499999999968 0.4048999999999978

0.6105700000000027 0.8316999999999979 0.4809800000000024

0.4887115926098389 0.9582391852389804 0.5563723022531519

0.3630840385578367 0.5790382736576305 0.6294053427680950

0.6518200000000007 0.9554699999999983 0.3668600000000026

0.5132100000000008 0.5396199999999993 0.4429400000000001

0.3831592869457552 0.6555129997594022 0.5184665196141864

0.5925754539411372 0.7791437115471496 0.6006649789698812

0.7491800000000026 0.7475499999999968 0.4048999999999978

0.9438999999999993 0.8316999999999979 0.4809800000000024

0.8241483129097844 0.9570005719989041 0.5577088040357978

0.6810495597629611 0.5375659414849636 0.6189963545954384

0.9851600000000005 0.9554699999999983 0.3668600000000026

0.8465399999999974 0.5396199999999993 0.4429400000000001

0.7197444559765671 0.6543429818796511 0.5191935165522210

0.9312179776515350 0.7717065896292965 0.6024777082218833

0.7034781630086967 0.6069255415334281 0.6767996956780192

Atomic coordinates of CO_2_* adsorption on the In (111) surface model

1.00000000000000

13.6434999999999995 0.0000000000000000 0.0000000000000000

-4.5478420000000002 11.0855879999999996 0.0000000000000000

0.0000000000000000 0.0000000000000000 27.2580999999999989

In O C

48 2 1

Direct

0.0825200000000024 0.2475499999999968 0.4048999999999978

0.2772300000000030 0.3316999999999979 0.4809800000000024

0.1510726331576109 0.4508418312064109 0.5561312133827414

0.0270663000866449 0.0748630005669284 0.6294707737829981

0.3184899999999971 0.4554699999999983 0.3668600000000026

0.1798700000000011 0.0396199999999993 0.4429400000000001

0.0506528521669480 0.1504404470893590 0.5182032930857403

0.2616137421423161 0.2711630319777925 0.6034566574023827

0.4158499999999989 0.2475499999999968 0.4048999999999978

0.6105700000000027 0.3316999999999979 0.4809800000000024

0.4867262125563414 0.4559549868039241 0.5569258255256742

0.3619375816773456 0.0715367062028136 0.6300107129312167

0.6518200000000007 0.4554699999999983 0.3668600000000026

0.5132100000000008 0.0396199999999993 0.4429400000000001

0.3851135203522205 0.1506595631294871 0.5190322460453274

0.5936565783781589 0.2722157373096081 0.6036797688715794

0.7491800000000026 0.2475499999999968 0.4048999999999978

0.9438999999999993 0.3316999999999979 0.4809800000000024

0.8197807205383114 0.4530624159177208 0.5550581904369025

0.6936959941767000 0.0725919864254365 0.6297117592623493

0.9851600000000005 0.4554699999999983 0.3668600000000026

0.8465399999999974 0.0396199999999993 0.4429400000000001

0.7165275693155735 0.1502778143664614 0.5188543959489422

0.9243633825625395 0.2713262513408807 0.6034591284303730

0.0825200000000024 0.7475499999999968 0.4048999999999978

0.2772300000000030 0.8316999999999979 0.4809800000000024

0.1549600321086615 0.9569921674518653 0.5563682173311051

0.0260875819535116 0.5732199417131767 0.6296315024337602

0.3184899999999971 0.9554699999999983 0.3668600000000026

0.1798700000000011 0.5396199999999993 0.4429400000000001

0.0515432160082909 0.6513803905852527 0.5186314801750953

0.2576135670277679 0.7739113332390275 0.6032632952747008

0.4158499999999989 0.7475499999999968 0.4048999999999978

0.6105700000000027 0.8316999999999979 0.4809800000000024

0.4876149108124679 0.9507341995029108 0.5565988897454304

0.3571211686568390 0.5780908453133335 0.6263546387260988

0.6518200000000007 0.9554699999999983 0.3668600000000026

0.5132100000000008 0.5396199999999993 0.4429400000000001

0.3852758540777259 0.6501817166115345 0.5171838261667716

0.5943095830676839 0.7686929907819433 0.6056788183355266

0.7491800000000026 0.7475499999999968 0.4048999999999978

0.9438999999999993 0.8316999999999979 0.4809800000000024

0.8189122295054432 0.9554676812278657 0.5562584858657473

0.6992483464365264 0.5722628145643327 0.6289300880464097

0.9851600000000005 0.9554699999999983 0.3668600000000026

0.8465399999999974 0.5396199999999993 0.4429400000000001

0.7183117393403293 0.6524658597412838 0.5190318794274447

0.9272155174112982 0.7746324201408967 0.6032952471162787

0.5518064304570061 0.5879268378519527 0.7408514045464898

0.4008609111073085 0.4104110152433620 0.7292589124424111

0.4761725551675724 0.4994031981189976 0.7346220741222480

Atomic coordinates of COOH* adsorption on the In (111) surface model

1.00000000000000

13.6434999999999995 0.0000000000000000 0.0000000000000000

-4.5478420000000002 11.0855879999999996 0.0000000000000000

0.0000000000000000 0.0000000000000000 27.2580999999999989

In C O H

48 1 2 1

Direct

0.0825200000000024 0.2475499999999968 0.4048999999999978

0.2772300000000030 0.3316999999999979 0.4809800000000024

0.1518576548772401 0.4461837644370874 0.5560889019993809

0.0318792589161561 0.0738023044514193 0.6305690405484583

0.3184899999999971 0.4554699999999983 0.3668600000000026

0.1798700000000011 0.0396199999999993 0.4429400000000001

0.0493639793577145 0.1480677684534797 0.5183161312267615

0.2618628298890613 0.2709866137300381 0.6032712662969146

0.4158499999999989 0.2475499999999968 0.4048999999999978

0.6105700000000027 0.3316999999999979 0.4809800000000024

0.4789513536767984 0.4522043628794847 0.5542660937938209

0.3509190474538713 0.0651193494735622 0.6294488733816692

0.6518200000000007 0.4554699999999983 0.3668600000000026

0.5132100000000008 0.0396199999999993 0.4429400000000001

0.3805802804681022 0.1452289741545840 0.5173306927203782

0.5811753168711178 0.2469934757653220 0.5990570735205480

0.7491800000000026 0.2475499999999968 0.4048999999999978

0.9438999999999993 0.3316999999999979 0.4809800000000024

0.8314770297371794 0.4527956051089603 0.5543855074272983

0.6940160904558610 0.0707858424692276 0.6305281113146748

0.9851600000000005 0.4554699999999983 0.3668600000000026

0.8465399999999974 0.0396199999999993 0.4429400000000001

0.7189053442282543 0.1475391803967838 0.5187948698578779

0.9259881214238561 0.2652329527630783 0.6038215313389941

0.0825200000000024 0.7475499999999968 0.4048999999999978

0.2772300000000030 0.8316999999999979 0.4809800000000024

0.1506066657284606 0.9512379542219378 0.5554822876438621

0.0364183512318590 0.5711851288357366 0.6301214499034913

0.3184899999999971 0.9554699999999983 0.3668600000000026

0.1798700000000011 0.5396199999999993 0.4429400000000001

0.0563274279435007 0.6530555705729171 0.5191537196981386

0.2628742319587403 0.7756798353972556 0.6035296249555770

0.4158499999999989 0.7475499999999968 0.4048999999999978

0.6105700000000027 0.8316999999999979 0.4809800000000024

0.4843135914647547 0.9505578292578321 0.5544433988549642

0.3568132837587399 0.5734327746644468 0.6293826951995314

0.6518200000000007 0.9554699999999983 0.3668600000000026

0.5132100000000008 0.5396199999999993 0.4429400000000001

0.3808863522930679 0.6499912020667864 0.5181571599847530

0.5874794482928252 0.7730390419794446 0.6044326552852013

0.7491800000000026 0.7475499999999968 0.4048999999999978

0.9438999999999993 0.8316999999999979 0.4809800000000024

0.8214324345758257 0.9508896806494889 0.5588828947271054

0.6791734203301516 0.5164756162678827 0.6164557998827291

0.9851600000000005 0.9554699999999983 0.3668600000000026

0.8465399999999974 0.5396199999999993 0.4429400000000001

0.7194767854894835 0.6596860827827129 0.5210357482075594

0.9295132454051824 0.7674567055175388 0.6030540742029373

0.6804310422629669 0.6219659550952059 0.6864815152946733

0.6620200294653245 0.7150607435625602 0.6886009565942004

0.7012859121296771 0.5725897680818193 0.7280607266044683

0.6980205512677456 0.6242036438627990 0.7554310581060389

Atomic coordinates of HCOO* adsorption on the In (111) surface model

1.00000000000000

13.6434999999999995 0.0000000000000000 0.0000000000000000

-4.5478420000000002 11.0855879999999996 0.0000000000000000

0.0000000000000000 0.0000000000000000 27.2580999999999989

In O C H

48 2 1 1

Direct

0.0825200000000024 0.2475499999999968 0.4048999999999978

0.2772300000000030 0.3316999999999979 0.4809800000000024

0.1506659851933136 0.4507384262951675 0.5554914243448508

0.0258283678164304 0.0587398077763197 0.6298058449353121

0.3184899999999971 0.4554699999999983 0.3668600000000026

0.1798700000000011 0.0396199999999993 0.4429400000000001

0.0487857406694374 0.1433386671557315 0.5178728440832572

0.2509321561270070 0.2652291482471014 0.6026051997846054

0.4158499999999989 0.2475499999999968 0.4048999999999978

0.6105700000000027 0.3316999999999979 0.4809800000000024

0.4742157295867315 0.4426037415560273 0.5566783562965938

0.3488653375849964 0.0648757014875159 0.6271507178712952

0.6518200000000007 0.4554699999999983 0.3668600000000026

0.5132100000000008 0.0396199999999993 0.4429400000000001

0.3808618723206507 0.1426341199845211 0.5166483307661451

0.5827282591186673 0.2491479984112033 0.6006447504210470

0.7491800000000026 0.2475499999999968 0.4048999999999978

0.9438999999999993 0.3316999999999979 0.4809800000000024

0.8254896105081642 0.4369928507565604 0.5561663242535531

0.7010609428896757 0.0730644593280658 0.6298063408364172

0.9851600000000005 0.4554699999999983 0.3668600000000026

0.8465399999999974 0.0396199999999993 0.4429400000000001

0.7182256017491824 0.1429738321294067 0.5172701150031642

0.9357396308227450 0.2640377095888482 0.6038053626598412

0.0825200000000024 0.7475499999999968 0.4048999999999978

0.2772300000000030 0.8316999999999979 0.4809800000000024

0.1480671704644649 0.9440560373443603 0.5548816272291148

0.0312686928955159 0.5727755252596296 0.6295113211380681

0.3184899999999971 0.9554699999999983 0.3668600000000026

0.1798700000000011 0.5396199999999993 0.4429400000000001

0.0506156986932425 0.6500934304618369 0.5181250625897960

0.2591535412542356 0.7717951268102468 0.6033133364862522

0.4158499999999989 0.7475499999999968 0.4048999999999978

0.6105700000000027 0.8316999999999979 0.4809800000000024

0.4881241722211627 0.9438983958477607 0.5572923166946353

0.3513460676595770 0.5714059981920769 0.6291348296599182

0.6518200000000007 0.9554699999999983 0.3668600000000026

0.5132100000000008 0.5396199999999993 0.4429400000000001

0.3782222796806920 0.6450189613119193 0.5167758492880239

0.5871866315271177 0.7561433780003313 0.6034136312729856

0.7491800000000026 0.7475499999999968 0.4048999999999978

0.9438999999999993 0.8316999999999979 0.4809800000000024

0.8140856482600540 0.9438229383315075 0.5569955940549391

0.6811925100496694 0.5307753743143451 0.6202503044909733

0.9851600000000005 0.9554699999999983 0.3668600000000026

0.8465399999999974 0.5396199999999993 0.4429400000000001

0.7152385842222415 0.6457442603335082 0.5167417553108891

0.9204000716615184 0.7599210929478986 0.6028284654761862

0.6399329665802753 0.7590750123896512 0.6873891445600364

0.6657914736822335 0.5831621717203532 0.6991556659891458

0.6531382178329892 0.6784742758617028 0.7131685335856019

0.6540222952513375 0.6908580150998275 0.7532526797294337

Atomic coordinates of CO* adsorption on the In (111) surface model

1.00000000000000

13.6434999999999995 0.0000000000000000 0.0000000000000000

-4.5478420000000002 11.0855879999999996 0.0000000000000000

0.0000000000000000 0.0000000000000000 27.2580999999999989

In C O

48 1 1

Direct

0.0825200000000024 0.2475499999999968 0.4048999999999978

0.2772300000000030 0.3316999999999979 0.4809800000000024

0.1510248331428912 0.4503973463717728 0.5559866780309406

0.0271369463012618 0.0771094661486202 0.6294072549257095

0.3184899999999971 0.4554699999999983 0.3668600000000026

0.1798700000000011 0.0396199999999993 0.4429400000000001

0.0489851820009619 0.1486747813893103 0.5181307575256895

0.2608823436706729 0.2706975307788682 0.6032809044537331

0.4158499999999989 0.2475499999999968 0.4048999999999978

0.6105700000000027 0.3316999999999979 0.4809800000000024

0.4941143275957813 0.4509386722829376 0.5604569704948901

0.3516273110335675 0.0616101681849202 0.6299115677766096

0.6518200000000007 0.4554699999999983 0.3668600000000026

0.5132100000000008 0.0396199999999993 0.4429400000000001

0.3836532201209621 0.1501984405660934 0.5189688814243567

0.5866350470144681 0.2509037945818804 0.6001763566614144

0.7491800000000026 0.2475499999999968 0.4048999999999978

0.9438999999999993 0.3316999999999979 0.4809800000000024

0.8117114665031480 0.4503772435905447 0.5570732377666445

0.6994753461043217 0.0651532252760063 0.6296470466066708

0.9851600000000005 0.4554699999999983 0.3668600000000026

0.8465399999999974 0.0396199999999993 0.4429400000000001

0.7188499617925999 0.1430063490272246 0.5175999279102612

0.9243041897120960 0.2710229250654876 0.6032689848708941

0.0825200000000024 0.7475499999999968 0.4048999999999978

0.2772300000000030 0.8316999999999979 0.4809800000000024

0.1544887297969169 0.9556647845832771 0.5540813082005371

0.0217480351873576 0.5654539818066979 0.6277227048053575

0.3184899999999971 0.9554699999999983 0.3668600000000026

0.1798700000000011 0.5396199999999993 0.4429400000000001

0.0491615264479961 0.6491909169675978 0.5176387076496454

0.2550393835609341 0.7680550300840423 0.6010597035180467

0.4158499999999989 0.7475499999999968 0.4048999999999978

0.6105700000000027 0.8316999999999979 0.4809800000000024

0.4862073792384223 0.9497253882059553 0.5566410805226525

0.3563419735386617 0.5710272383925101 0.6295953062599935

0.6518200000000007 0.9554699999999983 0.3668600000000026

0.5132100000000008 0.5396199999999993 0.4429400000000001

0.3828841162011827 0.6444390819258459 0.5178000265463325

0.5864540776030438 0.7701553026206867 0.6031173616334229

0.7491800000000026 0.7475499999999968 0.4048999999999978

0.9438999999999993 0.8316999999999979 0.4809800000000024

0.8186870656056158 0.9410518220361865 0.5562252348089629

0.7115161280294018 0.5998236297116080 0.6301388876362495

0.9851600000000005 0.9554699999999983 0.3668600000000026

0.8465399999999974 0.5396199999999993 0.4429400000000001

0.7148941297926115 0.6448581488464384 0.5167146668391851

0.9438892824059835 0.7820373064303681 0.6055331017474614

0.6385045340130802 0.3970775519407184 0.6808508605456041

0.6127985056020862 0.3722121609161774 0.7210249225676704

Atomic coordinates of HCOOH* adsorption on the In (111) surface model

1.00000000000000

13.6434999999999995 0.0000000000000000 0.0000000000000000

-4.5478420000000002 11.0855879999999996 0.0000000000000000

0.0000000000000000 0.0000000000000000 27.2580999999999989

In O C H

48 2 1 2

Direct

0.0825200000000024 0.2475499999999968 0.4048999999999978

0.2772300000000030 0.3316999999999979 0.4809800000000024

0.1526734449746385 0.4533068986073303 0.5564806454676443

0.0239621791420674 0.0639244942816291 0.6299634396828964

0.3184899999999971 0.4554699999999983 0.3668600000000026

0.1798700000000011 0.0396199999999993 0.4429400000000001

0.0498846222121614 0.1486033989071984 0.5181816834104680

0.2528388188139488 0.2676520200317114 0.6033926769668838

0.4158499999999989 0.2475499999999968 0.4048999999999978

0.6105700000000027 0.3316999999999979 0.4809800000000024

0.4764176895423446 0.4438105441227194 0.5562792877616403

0.3566824711304051 0.0730999087567668 0.6293337428224461

0.6518200000000007 0.4554699999999983 0.3668600000000026

0.5132100000000008 0.0396199999999993 0.4429400000000001

0.3820528400705143 0.1444079071722371 0.5178240412929327

0.5916640902848656 0.2583720215204508 0.6029770024579548

0.7491800000000026 0.2475499999999968 0.4048999999999978

0.9438999999999993 0.3316999999999979 0.4809800000000024

0.8236433014628091 0.4426282575970006 0.5561280391261079

0.6961158580261753 0.0621657656898904 0.6297879674913531

0.9851600000000005 0.4554699999999983 0.3668600000000026

0.8465399999999974 0.0396199999999993 0.4429400000000001

0.7173333760074314 0.1439370259541946 0.5179458501124543

0.9265966674531896 0.2618468179701594 0.6033049384087334

0.0825200000000024 0.7475499999999968 0.4048999999999978

0.2772300000000030 0.8316999999999979 0.4809800000000024

0.1503018538247574 0.9504113810083723 0.5563455035932563

0.0256822837262618 0.5680918190461952 0.6299534636716828

0.3184899999999971 0.9554699999999983 0.3668600000000026

0.1798700000000011 0.5396199999999993 0.4429400000000001

0.0517968923074221 0.6511741744417634 0.5189948740363377

0.2567497779267356 0.7721546748948853 0.6039638658310409

0.4158499999999989 0.7475499999999968 0.4048999999999978

0.6105700000000027 0.8316999999999979 0.4809800000000024

0.4860083233485426 0.9501047745955873 0.5570571367078188

0.3578198178266945 0.5741068699885332 0.6301269249806722

0.6518200000000007 0.9554699999999983 0.3668600000000026

0.5132100000000008 0.5396199999999993 0.4429400000000001

0.3836585285546761 0.6503615870067421 0.5189162198186210

0.5920211202757528 0.7629862458743943 0.6000240059177955

0.7491800000000026 0.7475499999999968 0.4048999999999978

0.9438999999999993 0.8316999999999979 0.4809800000000024

0.8204548224757744 0.9460334361461520 0.5559385381271440

0.6843914701912368 0.5504251014678019 0.6211894552234725

0.9851600000000005 0.9554699999999983 0.3668600000000026

0.8465399999999974 0.5396199999999993 0.4429400000000001

0.7175366413787927 0.6470977750649496 0.5147124530118887

0.9193384909013815 0.7598611875016488 0.6037999502242428

0.6781996011397808 0.5725191785059138 0.7256477679926263

0.7127137930676852 0.7748299329296060 0.7255996931174522

0.7032764849214388 0.6699202102506092 0.7463293358550089

0.7220348463414865 0.6833081577263371 0.7858946402844680

0.6954559749493184 0.7619830531466006 0.6896744526616186

Atomic coordinates of the In_2_O_3_ (110) surface model

1.00000000000000

10.2365999999999993 0.0000000000000000 0.0000000000000000

0.0000000000000000 14.4766999999999992 0.0000000000000000

0.0000000000000000 0.0000000000000000 25.9995000000000012

In O

32 48

Direct

0.7013999999999996 0.2500000000000000 0.4667200000000022

0.4851786790483601 0.3747325131947221 0.5380477615212058

0.7672286601518366 0.9975218644367345 0.6012529104621223

0.9851786790483601 0.8747325131947221 0.5380477615212058

0.4853600000000000 0.1250000000000000 0.3971200000000010

0.9853600000000000 0.6250000000000000 0.3971200000000010

0.2750110431585426 0.0006158882281255 0.6020453341422467

0.4853600000000000 0.6250000000000000 0.3971200000000010

0.2013999999999996 0.2500000000000000 0.4667200000000022

0.9853600000000000 0.1250000000000000 0.3971200000000010

0.4883019307143570 0.8737012494063237 0.5389357411123612

0.9883019307143570 0.3737012494063237 0.5389357411123612

0.4870262679045467 0.6074120496229369 0.5284828380656990

0.9890332158436124 0.6409661458262974 0.5484498918679037

0.9853600000000000 0.3919799999999967 0.4065799999999982

0.4853600000000000 0.3580200000000033 0.3876700000000000

0.2415269054492626 0.2712402964709568 0.5955008313912700

0.2353600000000000 0.9830200000000033 0.4761799999999994

0.2554896170065106 0.7264731263198314 0.6053079327944317

0.2353600000000000 0.5169799999999967 0.4572700000000012

0.2013999999999996 0.7500000000000000 0.4667200000000022

0.2672286601518366 0.4975218644367345 0.6012529104621223

0.7750110431585426 0.5006158882281255 0.6020453341422467

0.7013999999999996 0.7500000000000000 0.4667200000000022

0.9870262679045467 0.1074120496229369 0.5284828380656990

0.4890332158436124 0.1409661458262974 0.5484498918679037

0.4853600000000000 0.8919799999999967 0.4065799999999982

0.9853600000000000 0.8580200000000033 0.3876700000000000

0.7415269054492626 0.7712402964709568 0.5955008313912700

0.7353600000000000 0.4830200000000033 0.4761799999999994

0.7554896170065106 0.2264731263198243 0.6053079327944317

0.7353600000000000 0.0169799999999967 0.4572700000000012

0.0806299999999993 0.6209899999999990 0.4729299999999981

0.5786349147505163 0.7375296232151385 0.5389706910472469

0.8958928745166546 0.5090462824602042 0.5347704868981253

0.6267505887396183 0.1088709440551128 0.6091752942715445

0.0806299999999993 0.2611500000000007 0.3948899999999966

0.8532300000000035 0.3525500000000008 0.4707799999999978

0.3900900000000007 0.4888499999999993 0.3993600000000015

0.8906151814975232 0.8767171035695540 0.6131573513729620

0.5806299999999993 0.3790100000000010 0.4605099999999993

0.3178236192047166 0.1388535263123600 0.6146224209510365

0.1174899999999965 0.5072800000000015 0.3846199999999982

0.1255200000000016 0.1065699999999978 0.4604300000000023

0.1255200000000016 0.7387000000000015 0.3868599999999986

0.3451999999999984 0.0112999999999985 0.4073800000000034

0.6222447970016347 0.8918884089263557 0.6007623890254195

0.3333690579588477 0.8609908164198927 0.6076988132005638

0.1255200000000016 0.3934300000000022 0.4730100000000022

0.1274462988633829 0.7641828799616093 0.5456035082398358

0.3503058783656243 0.4890356221096610 0.5232824796015692

0.3532300000000035 0.2427199999999985 0.4096199999999968

0.3532300000000035 0.8525500000000008 0.4707799999999978

0.1222447970016347 0.3918884089263557 0.6007623890254195

0.3532300000000035 0.6474499999999992 0.4626700000000028

0.1267505887396183 0.6088709440551128 0.6091752942715445

0.6174899999999965 0.0072800000000015 0.3846199999999982

0.1185134472146103 0.9928453501263732 0.5502448237273256

0.8532300000000035 0.7427199999999985 0.4096199999999968

0.6185134472146103 0.4928453501263732 0.5502448237273256

0.8561570793920765 0.7531859549720750 0.5247490278794942

0.8960052448606106 0.1216167880361780 0.6031411380800549

0.8532300000000035 0.1474499999999992 0.4626700000000028

0.5806299999999993 0.1209899999999990 0.4729299999999981

0.0786349147505163 0.2375296232151385 0.5389706910472469

0.3958928745166546 0.0090462824602042 0.5347704868981253

0.3561570793920765 0.2531859549720750 0.5247490278794942

0.5806299999999993 0.7611500000000007 0.3948899999999966

0.8900900000000007 0.9888499999999993 0.3993600000000015

0.3906151814975232 0.3767171035695540 0.6131573513729620

0.0806299999999993 0.8790100000000010 0.4605099999999993

0.8178236192047166 0.6388535263123600 0.6146224209510365

0.3960052448606106 0.6216167880361780 0.6031411380800549

0.6255200000000016 0.6065699999999978 0.4604300000000023

0.6255200000000016 0.2387000000000015 0.3868599999999986

0.8451999999999984 0.5112999999999985 0.4073800000000034

0.8333690579588477 0.3609908164198927 0.6076988132005638

0.6255200000000016 0.8934300000000022 0.4730100000000022

0.6274462988633829 0.2641828799616093 0.5456035082398358

0.8503058783656243 0.9890356221096610 0.5232824796015692

Atomic coordinates of H* adsorption on the In_2_O_3_ (110) surface model

1.00000000000000

10.2365999999999993 0.0000000000000000 0.0000000000000000

0.0000000000000000 14.4766999999999992 0.0000000000000000

0.0000000000000000 0.0000000000000000 25.9995000000000012

In O H

32 48 1

Direct

0.7013999999999996 0.2500000000000000 0.4667200000000022

0.4869675881558777 0.3736712997969391 0.5372847595377550

0.7690509633769551 0.9976330655784693 0.6015924701104538

0.9857864368526563 0.8756104095808936 0.5377254381534229

0.4853600000000000 0.1250000000000000 0.3971200000000010

0.9853600000000000 0.6250000000000000 0.3971200000000010

0.2743333253107423 0.0036218931074146 0.6024436360149963

0.4853600000000000 0.6250000000000000 0.3971200000000010

0.2013999999999996 0.2500000000000000 0.4667200000000022

0.9853600000000000 0.1250000000000000 0.3971200000000010

0.4908678329029996 0.8750220725070506 0.5386013642345588

0.9890340059508915 0.3732368393547105 0.5386388473364789

0.4858047508561469 0.6077176321894910 0.5276057892156558

0.9814514895927502 0.6425616634988884 0.5447990748561580

0.9853600000000000 0.3919799999999967 0.4065799999999982

0.4853600000000000 0.3580200000000033 0.3876700000000000

0.2425123555621980 0.2708832839175912 0.5955068927465206

0.2353600000000000 0.9830200000000033 0.4761799999999994

0.2568642851749559 0.7339777209730798 0.6061901647633761

0.2353600000000000 0.5169799999999967 0.4572700000000012

0.2013999999999996 0.7500000000000000 0.4667200000000022

0.2752582021971790 0.4926086147254978 0.6022633403311559

0.7746916677465236 0.4990106578443232 0.6021212593819456

0.7013999999999996 0.7500000000000000 0.4667200000000022

0.9869758088369736 0.1075779089587741 0.5279338847099169

0.4907382653242891 0.1411852841511134 0.5484702930025449

0.4853600000000000 0.8919799999999967 0.4065799999999982

0.9853600000000000 0.8580200000000033 0.3876700000000000

0.7437010658861212 0.7716994994338648 0.5964421992076296

0.7353600000000000 0.4830200000000033 0.4761799999999994

0.7568583683820691 0.2255308846603299 0.6055338000520436

0.7353600000000000 0.0169799999999967 0.4572700000000012

0.0806299999999993 0.6209899999999990 0.4729299999999981

0.5783095775908293 0.7362480257382416 0.5413626066508925

0.8964653865338974 0.5090917101477004 0.5350948244370883

0.6278234573623962 0.1085159021909305 0.6097417317551361

0.0806299999999993 0.2611500000000007 0.3948899999999966

0.8532300000000035 0.3525500000000008 0.4707799999999978

0.3900900000000007 0.4888499999999993 0.3993600000000015

0.8940019846764358 0.8754892555126617 0.6133533041319978

0.5806299999999993 0.3790100000000010 0.4605099999999993

0.3198340973025680 0.1402802156099554 0.6154961348881258

0.1174899999999965 0.5072800000000015 0.3846199999999982

0.1255200000000016 0.1065699999999978 0.4604300000000023

0.1255200000000016 0.7387000000000015 0.3868599999999986

0.3451999999999984 0.0112999999999985 0.4073800000000034

0.6240665789015267 0.8919600794397056 0.6012642443153950

0.3341337497357344 0.8648573119629219 0.6096091121793705

0.1255200000000016 0.3934300000000022 0.4730100000000022

0.1253129813829688 0.7586580644459104 0.5468349936405659

0.3502423815927784 0.4889039669535507 0.5241037248122922

0.3532300000000035 0.2427199999999985 0.4096199999999968

0.3532300000000035 0.8525500000000008 0.4707799999999978

0.1244909004463253 0.3953706942339608 0.6013816584845060

0.3532300000000035 0.6474499999999992 0.4626700000000028

0.1147827964573835 0.6127929686637472 0.6167735603358935

0.6174899999999965 0.0072800000000015 0.3846199999999982

0.1187479267657991 0.9924062325403185 0.5505443522989424

0.8532300000000035 0.7427199999999985 0.4096199999999968

0.6169325554196021 0.4943895396756801 0.5512627246367288

0.8543551669582001 0.7554347607330172 0.5246376549996725

0.8979824702432069 0.1213134527622728 0.6030359476635709

0.8532300000000035 0.1474499999999992 0.4626700000000028

0.5806299999999993 0.1209899999999990 0.4729299999999981

0.0801152687629170 0.2380378792135147 0.5402819763403599

0.3960713204086659 0.0092823985008437 0.5353260714086403

0.3568677948246943 0.2536009495494227 0.5248957719223952

0.5806299999999993 0.7611500000000007 0.3948899999999966

0.8900900000000007 0.9888499999999993 0.3993600000000015

0.3957246501733493 0.3734289747928941 0.6137024530267894

0.0806299999999993 0.8790100000000010 0.4605099999999993

0.8269865451418781 0.6378422723457504 0.6152297292974325

0.3800494112773833 0.6212923336365961 0.6023557020047221

0.6255200000000016 0.6065699999999978 0.4604300000000023

0.6255200000000016 0.2387000000000015 0.3868599999999986

0.8451999999999984 0.5112999999999985 0.4073800000000034

0.8366497281593510 0.3602331015250329 0.6076410252623248

0.6255200000000016 0.8934300000000022 0.4730100000000022

0.6289966993713492 0.2638635272290202 0.5459742157160576

0.8499486987825193 0.9887109626709005 0.5236495209558427

0.0452976113997607 0.6092243782296762 0.6422950302117459

Atomic coordinates of CO_2_* adsorption on the In_2_O_3_ (110) surface model

1.00000000000000

10.2365999999999993 0.0000000000000000 0.0000000000000000

0.0000000000000000 14.4766999999999992 0.0000000000000000

0.0000000000000000 0.0000000000000000 25.9995000000000012

In O C

32 50 1

Direct

0.7013999999999996 0.2500000000000000 0.4667200000000022

0.4834310104829811 0.3741367650833283 0.5369387404918911

0.7678707916762662 0.0010944559118755 0.6008771250474467

0.9831656286985080 0.8734391844435976 0.5382788934550646

0.4853600000000000 0.1250000000000000 0.3971200000000010

0.9853600000000000 0.6250000000000000 0.3971200000000010

0.2743851985323928 0.0007777421622848 0.6021979468496852

0.4853600000000000 0.6250000000000000 0.3971200000000010

0.2013999999999996 0.2500000000000000 0.4667200000000022

0.9853600000000000 0.1250000000000000 0.3971200000000010

0.4859052021986301 0.8752385842932497 0.5380862859568794

0.9889115986692758 0.3730744241167088 0.5390931013540623

0.4884044169411439 0.6077934073189795 0.5288659943752165

0.9918871756368759 0.6389353639093258 0.5461393542686608

0.9853600000000000 0.3919799999999967 0.4065799999999982

0.4853600000000000 0.3580200000000033 0.3876700000000000

0.2421143582860807 0.2711166959783355 0.5955354318471748

0.2353600000000000 0.9830200000000033 0.4761799999999994

0.2547019877499039 0.7269506839402737 0.6053207402757792

0.2353600000000000 0.5169799999999967 0.4572700000000012

0.2013999999999996 0.7500000000000000 0.4667200000000022

0.2676891917784374 0.4974760499106168 0.6011866165304127

0.7643470909737431 0.4811125096815303 0.6057803700685938

0.7013999999999996 0.7500000000000000 0.4667200000000022

0.9884029263513909 0.1067631524329471 0.5282706235385106

0.4887567060929072 0.1413971173462514 0.5487469571175865

0.4853600000000000 0.8919799999999967 0.4065799999999982

0.9853600000000000 0.8580200000000033 0.3876700000000000

0.7316867974207426 0.7822644475312828 0.5990620678918503

0.7353600000000000 0.4830200000000033 0.4761799999999994

0.7544995620818327 0.2231210648902930 0.6053044929718041

0.7353600000000000 0.0169799999999967 0.4572700000000012

0.0806299999999993 0.6209899999999990 0.4729299999999981

0.5783026432689198 0.7393721270522420 0.5401186503811886

0.8905831451293125 0.5088878757800970 0.5393416970045095

0.6245994013074707 0.1107870023476067 0.6107317032415835

0.0806299999999993 0.2611500000000007 0.3948899999999966

0.8532300000000035 0.3525500000000008 0.4707799999999978

0.3900900000000007 0.4888499999999993 0.3993600000000015

0.8881774001961276 0.8792036175172271 0.6130676289495227

0.5806299999999993 0.3790100000000010 0.4605099999999993

0.3176406304010797 0.1388568495746654 0.6148125122184638

0.1174899999999965 0.5072800000000015 0.3846199999999982

0.1255200000000016 0.1065699999999978 0.4604300000000023

0.1255200000000016 0.7387000000000015 0.3868599999999986

0.3451999999999984 0.0112999999999985 0.4073800000000034

0.6163678172628551 0.9030847171003984 0.5986229438070438

0.3322246171658136 0.8610684169091698 0.6073987477526970

0.1255200000000016 0.3934300000000022 0.4730100000000022

0.1269464201372728 0.7640498899292041 0.5451153883577859

0.3502456036358907 0.4896895322722585 0.5229824346216390

0.3532300000000035 0.2427199999999985 0.4096199999999968

0.3532300000000035 0.8525500000000008 0.4707799999999978

0.1236152153298917 0.3924785787980127 0.6005890066610533

0.3532300000000035 0.6474499999999992 0.4626700000000028

0.1228768005238763 0.6099044875190032 0.6086547641283033

0.6174899999999965 0.0072800000000015 0.3846199999999982

0.1177801558599754 0.9912909965297558 0.5503450333714284

0.8532300000000035 0.7427199999999985 0.4096199999999968

0.6180671064128873 0.4907809736239628 0.5499817115362546

0.8527209677932248 0.7487492865838590 0.5285875383114700

0.8974764901797911 0.1202925688920118 0.6027059192480237

0.8532300000000035 0.1474499999999992 0.4626700000000028

0.5806299999999993 0.1209899999999990 0.4729299999999981

0.0801567243864980 0.2371429931349809 0.5389329901587203

0.3931347322427783 0.0109197053189973 0.5343681648273062

0.3559497537176171 0.2526578789804290 0.5243094671642439

0.5806299999999993 0.7611500000000007 0.3948899999999966

0.8900900000000007 0.9888499999999993 0.3993600000000015

0.3918248220229543 0.3757560028966225 0.6121099500939309

0.0806299999999993 0.8790100000000010 0.4605099999999993

0.7943535082148685 0.6351956539860097 0.6165231424247182

0.3927113647372167 0.6212826322196250 0.6021755154769792

0.6255200000000016 0.6065699999999978 0.4604300000000023

0.6255200000000016 0.2387000000000015 0.3868599999999986

0.8451999999999984 0.5112999999999985 0.4073800000000034

0.8443254503416213 0.3501810680716275 0.6073414865115865

0.6255200000000016 0.8934300000000022 0.4730100000000022

0.6276813884925119 0.2643023243316449 0.5454678494826055

0.8506811611180254 0.9884527352234969 0.5227415577169339

0.6667538284785266 0.7077979035815218 0.6731577656631842

0.6901379171999764 0.5492802032418425 0.6748646360441626

0.7142704278560502 0.6317404169985609 0.6574411059801452

Atomic coordinates of COOH* adsorption on the In_2_O_3_ (110) surface model

1.00000000000000

10.2365999999999993 0.0000000000000000 0.0000000000000000

0.0000000000000000 14.4766999999999992 0.0000000000000000

0.0000000000000000 0.0000000000000000 25.9995000000000012

In O C H

32 50 1 1

Direct

0.7013999999999996 0.2500000000000000 0.4667200000000022

0.4806670888534654 0.3730609519428114 0.5359568761360123

0.7681564554369444 0.9983503995824208 0.6006988824745605

0.9865507118488850 0.8727026905229565 0.5380563890296628

0.4853600000000000 0.1250000000000000 0.3971200000000010

0.9853600000000000 0.6250000000000000 0.3971200000000010

0.2742111930321087 0.0012854511711140 0.6026482330399361

0.4853600000000000 0.6250000000000000 0.3971200000000010

0.2013999999999996 0.2500000000000000 0.4667200000000022

0.9853600000000000 0.1250000000000000 0.3971200000000010

0.4842250792628207 0.8758049053199315 0.5377326309907815

0.9911872590655832 0.3713460627661220 0.5389677504782426

0.4864935108913784 0.6078337763517752 0.5281642077830000

0.9996822843883280 0.6369762552621552 0.5465500083281398

0.9853600000000000 0.3919799999999967 0.4065799999999982

0.4853600000000000 0.3580200000000033 0.3876700000000000

0.2419749600652068 0.2702507517571391 0.5962961843205150

0.2353600000000000 0.9830200000000033 0.4761799999999994

0.2572489765721429 0.7273447055054021 0.6061340393636740

0.2353600000000000 0.5169799999999967 0.4572700000000012

0.2013999999999996 0.7500000000000000 0.4667200000000022

0.2693459073038795 0.4971174785746442 0.6019593059571449

0.7600356064425497 0.4691334759892669 0.6040007589916243

0.7013999999999996 0.7500000000000000 0.4667200000000022

0.9883807194523229 0.1057814535423844 0.5277559478415697

0.4884150633773245 0.1410362602538271 0.5480312456910852

0.4853600000000000 0.8919799999999967 0.4065799999999982

0.9853600000000000 0.8580200000000033 0.3876700000000000

0.7320733647192270 0.7834478009832821 0.5938093303374288

0.7353600000000000 0.4830200000000033 0.4761799999999994

0.7550472101386134 0.2182360258331357 0.6045467584997795

0.7353600000000000 0.0169799999999967 0.4572700000000012

0.0806299999999993 0.6209899999999990 0.4729299999999981

0.5774884769312862 0.7392486291454716 0.5398753985461440

0.8897309950610151 0.5101167969861180 0.5439578380937107

0.6239792891383331 0.1069604392821404 0.6095648647955940

0.0806299999999993 0.2611500000000007 0.3948899999999966

0.8532300000000035 0.3525500000000008 0.4707799999999978

0.3900900000000007 0.4888499999999993 0.3993600000000015

0.8882775032350452 0.8752494855850799 0.6131982062437942

0.5806299999999993 0.3790100000000010 0.4605099999999993

0.3185756756259153 0.1389384877276072 0.6154368622599691

0.1174899999999965 0.5072800000000015 0.3846199999999982

0.1255200000000016 0.1065699999999978 0.4604300000000023

0.1255200000000016 0.7387000000000015 0.3868599999999986

0.3451999999999984 0.0112999999999985 0.4073800000000034

0.6141613678090465 0.9003226433000648 0.6006111054240719

0.3334584952377426 0.8612854367038096 0.6082967463853706

0.1255200000000016 0.3934300000000022 0.4730100000000022

0.1293188903177338 0.7628299607945124 0.5456851690899569

0.3490668268845667 0.4901659653205144 0.5238201394620319

0.3532300000000035 0.2427199999999985 0.4096199999999968

0.3532300000000035 0.8525500000000008 0.4707799999999978

0.1244614653782889 0.3929331616374228 0.6008136418515022

0.3532300000000035 0.6474499999999992 0.4626700000000028

0.1249316900408530 0.6107266780532186 0.6111812876523146

0.6174899999999965 0.0072800000000015 0.3846199999999982

0.1179443665641671 0.9904732552996265 0.5508884557373577

0.8532300000000035 0.7427199999999985 0.4096199999999968

0.6164444941187881 0.4903387174196041 0.5500268369339594

0.8547047515855439 0.7426029561689873 0.5300423587813441

0.8987471019242008 0.1163325322384026 0.6029378453892420

0.8532300000000035 0.1474499999999992 0.4626700000000028

0.5806299999999993 0.1209899999999990 0.4729299999999981

0.0805514136528771 0.2359301037938764 0.5399668312646355

0.3928037639576090 0.0107631142274514 0.5347115258801480

0.3552243207659274 0.2523061340297730 0.5248062004143179

0.5806299999999993 0.7611500000000007 0.3948899999999966

0.8900900000000007 0.9888499999999993 0.3993600000000015

0.3929666681326367 0.3755452976924829 0.6123492021779100

0.0806299999999993 0.8790100000000010 0.4605099999999993

0.7498544406838974 0.6498734146230944 0.6330870062202791

0.3967342846052802 0.6211223731947015 0.6040326818786994

0.6255200000000016 0.6065699999999978 0.4604300000000023

0.6255200000000016 0.2387000000000015 0.3868599999999986

0.8451999999999984 0.5112999999999985 0.4073800000000034

0.8495974843097827 0.3435935610885110 0.6093737403068147

0.6255200000000016 0.8934300000000022 0.4730100000000022

0.6282299782780072 0.2647341312281242 0.5460467883441140

0.8505924293386968 0.9856642865512910 0.5230448862112098

0.6367639998899790 0.6654302663937131 0.7065473284614114

0.6932551530616493 0.5238534790861635 0.6773145556566149

0.6941363043800592 0.6115547409475752 0.6714041463128098

0.5949862115098911 0.6254042536936311 0.7316469118554824

Atomic coordinates of HCOO* adsorption on the In_2_O_3_ (110) surface model

1.00000000000000

10.2365999999999993 0.0000000000000000 0.0000000000000000

0.0000000000000000 14.4766999999999992 0.0000000000000000

0.0000000000000000 0.0000000000000000 25.9995000000000012

In O C H

32 50 1 1

Direct

0.7013999999999996 0.2500000000000000 0.4667200000000022

0.4875558940674125 0.3749093544093967 0.5381707800784881

0.7677093410176212 0.9976801933242143 0.6012227319015864

0.9822047127032221 0.8733888805251269 0.5378645983170287

0.4853600000000000 0.1250000000000000 0.3971200000000010

0.9853600000000000 0.6250000000000000 0.3971200000000010

0.2750746142281102 0.9995986491980275 0.6016808047283888

0.4853600000000000 0.6250000000000000 0.3971200000000010

0.2013999999999996 0.2500000000000000 0.4667200000000022

0.9853600000000000 0.1250000000000000 0.3971200000000010

0.4894899626376059 0.8727014450371442 0.5391350990177273

0.9845878754074207 0.3736619372421899 0.5402733199000878

0.4882920122113106 0.6066517981315300 0.5286456889470728

0.9879947583053195 0.6406162193317968 0.5481187871741255

0.9853600000000000 0.3919799999999967 0.4065799999999982

0.4853600000000000 0.3580200000000033 0.3876700000000000

0.2418677637738682 0.2697383514561338 0.5950146395767533

0.2353600000000000 0.9830200000000033 0.4761799999999994

0.2562306194267236 0.7264320951513525 0.6054065776419506

0.2353600000000000 0.5169799999999967 0.4572700000000012

0.2013999999999996 0.7500000000000000 0.4667200000000022

0.2683962719395367 0.4963959796543520 0.6012217627110559

0.7710976000803669 0.5040890555045436 0.6053082357324726

0.7013999999999996 0.7500000000000000 0.4667200000000022

0.9875309449304766 0.1071624476429918 0.5287802575760381

0.4892104906926349 0.1405597787876829 0.5488043932135298

0.4853600000000000 0.8919799999999967 0.4065799999999982

0.9853600000000000 0.8580200000000033 0.3876700000000000

0.7398911484928234 0.7663549138731796 0.5996461478289135

0.7353600000000000 0.4830200000000033 0.4761799999999994

0.7563127681797610 0.2270510173069127 0.6055460836612738

0.7353600000000000 0.0169799999999967 0.4572700000000012

0.0806299999999993 0.6209899999999990 0.4729299999999981

0.5788471594437752 0.7368139431505938 0.5391923099344211

0.8937072656754168 0.5073500121525001 0.5345506764499817

0.6276406328490367 0.1091635349432210 0.6093683640265439

0.0806299999999993 0.2611500000000007 0.3948899999999966

0.8532300000000035 0.3525500000000008 0.4707799999999978

0.3900900000000007 0.4888499999999993 0.3993600000000015

0.8892522074580000 0.8768706551405288 0.6133327174278875

0.5806299999999993 0.3790100000000010 0.4605099999999993

0.3184379036486220 0.1376938599439725 0.6144098002065945

0.1174899999999965 0.5072800000000015 0.3846199999999982

0.1255200000000016 0.1065699999999978 0.4604300000000023

0.1255200000000016 0.7387000000000015 0.3868599999999986

0.3451999999999984 0.0112999999999985 0.4073800000000034

0.6249581609217003 0.8923353383634876 0.5998255685314788

0.3354177619996719 0.8604482292349118 0.6076329898351034

0.1255200000000016 0.3934300000000022 0.4730100000000022

0.1276973496372946 0.7634784121464975 0.5455739627245961

0.3507251603659540 0.4883201488818543 0.5231284071197777

0.3532300000000035 0.2427199999999985 0.4096199999999968

0.3532300000000035 0.8525500000000008 0.4707799999999978

0.1238636952185743 0.3907527864884770 0.6011707214430757

0.3532300000000035 0.6474499999999992 0.4626700000000028

0.1313885147819676 0.6075498408219389 0.6094733532507774

0.6174899999999965 0.0072800000000015 0.3846199999999982

0.1179690141344807 0.9916227583912303 0.5501925041081108

0.8532300000000035 0.7427199999999985 0.4096199999999968

0.6196073253436225 0.4930515130201627 0.5498271254896636

0.8537514829387547 0.7562492242662060 0.5238344973988305

0.8960221100046084 0.1211386147833906 0.6031982910788400

0.8532300000000035 0.1474499999999992 0.4626700000000028

0.5806299999999993 0.1209899999999990 0.4729299999999981

0.0785092017871349 0.2375986039589009 0.5388880479404676

0.3964793910920861 0.0083943391644254 0.5345619867831530

0.3568002492764819 0.2526756025983090 0.5243337393619782

0.5806299999999993 0.7611500000000007 0.3948899999999966

0.8900900000000007 0.9888499999999993 0.3993600000000015

0.3903584708776648 0.3752105500590091 0.6126657439652092

0.0806299999999993 0.8790100000000010 0.4605099999999993

0.8462288440039742 0.6406255138255474 0.6092013522119402

0.3951260043882172 0.6206794097006849 0.6026790303016298

0.6255200000000016 0.6065699999999978 0.4604300000000023

0.6255200000000016 0.2387000000000015 0.3868599999999986

0.8451999999999984 0.5112999999999985 0.4073800000000034

0.8334922055128473 0.3603661726154854 0.6057748016707620

0.6255200000000016 0.8934300000000022 0.4730100000000022

0.6273013495098638 0.2629057031027102 0.5454299596440748

0.8505654161364689 0.9898810780812468 0.5230822869722971

0.6315976125148737 0.7020650883819144 0.6674337392711962

0.6562720430427973 0.5428907232791076 0.6727823678412861

0.6161944154143129 0.6228950139285629 0.6866422996158121

0.5573479284972294 0.6209554431459097 0.7221958505431775

Atomic coordinates of CO* adsorption on the In_2_O_3_ (110) surface model

1.00000000000000

10.2365999999999993 0.0000000000000000 0.0000000000000000

0.0000000000000000 14.4766999999999992 0.0000000000000000

0.0000000000000000 0.0000000000000000 25.9995000000000012

In O C

32 49 1

Direct

0.7013999999999996 0.2500000000000000 0.4667200000000022

0.4854579209620837 0.3745317400386128 0.5378886599371242

0.7675780430359609 0.9977368630695125 0.6009770553495102

0.9851165518216405 0.8743915525737478 0.5382058932980129

0.4853600000000000 0.1250000000000000 0.3971200000000010

0.9853600000000000 0.6250000000000000 0.3971200000000010

0.2751734854577350 0.0001214434245895 0.6021291194800398

0.4853600000000000 0.6250000000000000 0.3971200000000010

0.2013999999999996 0.2500000000000000 0.4667200000000022

0.9853600000000000 0.1250000000000000 0.3971200000000010

0.4886052446127565 0.8739762625560772 0.5388462308088720

0.9887552262588528 0.3734213265123500 0.5388031933897395

0.4875221181179867 0.6074034113331948 0.5285724115667350

0.9888934173803179 0.6405404420237701 0.5487794496594773

0.9853600000000000 0.3919799999999967 0.4065799999999982

0.4853600000000000 0.3580200000000033 0.3876700000000000

0.2419853559634504 0.2708563722906661 0.5954422676196600

0.2353600000000000 0.9830200000000033 0.4761799999999994

0.2557458803187345 0.7260492743456908 0.6052470416998474

0.2353600000000000 0.5169799999999967 0.4572700000000012

0.2013999999999996 0.7500000000000000 0.4667200000000022

0.2677294324031010 0.4970897193614618 0.6012279381724355

0.7760962851123168 0.4995684825945901 0.6016682522161076

0.7013999999999996 0.7500000000000000 0.4667200000000022

0.9874931391958128 0.1072861966067507 0.5284662545707377

0.4893562808561001 0.1408429154462141 0.5486115438353423

0.4853600000000000 0.8919799999999967 0.4065799999999982

0.9853600000000000 0.8580200000000033 0.3876700000000000

0.7404130567015414 0.7706739382792804 0.5960186893792070

0.7353600000000000 0.4830200000000033 0.4761799999999994

0.7558195970972648 0.2264087107441739 0.6053069537038027

0.7353600000000000 0.0169799999999967 0.4572700000000012

0.0806299999999993 0.6209899999999990 0.4729299999999981

0.5788001019388034 0.7376051144523785 0.5394655518402658

0.8963748513328369 0.5084483019952444 0.5342374427152308

0.6269535051911745 0.1092965430835662 0.6093912687802288

0.0806299999999993 0.2611500000000007 0.3948899999999966

0.8532300000000035 0.3525500000000008 0.4707799999999978

0.3900900000000007 0.4888499999999993 0.3993600000000015

0.8903608697069885 0.8765084460083017 0.6132916375893132

0.5806299999999993 0.3790100000000010 0.4605099999999993

0.3177654136286066 0.1384547020277651 0.6149614269338670

0.1174899999999965 0.5072800000000015 0.3846199999999982

0.1255200000000016 0.1065699999999978 0.4604300000000023

0.1255200000000016 0.7387000000000015 0.3868599999999986

0.3451999999999984 0.0112999999999985 0.4073800000000034

0.6224504551848966 0.8933310118979847 0.6001073499898837

0.3342322070229571 0.8605168426823653 0.6084188705008202

0.1255200000000016 0.3934300000000022 0.4730100000000022

0.1275612958112191 0.7637405480454476 0.5456448970469623

0.3505603058742039 0.4890554850956761 0.5232765561931245

0.3532300000000035 0.2427199999999985 0.4096199999999968

0.3532300000000035 0.8525500000000008 0.4707799999999978

0.1229406047322641 0.3917241788169790 0.6006917800446843

0.3532300000000035 0.6474499999999992 0.4626700000000028

0.1272183752144826 0.6084375002767217 0.6094753817385907

0.6174899999999965 0.0072800000000015 0.3846199999999982

0.1185931097557145 0.9925613614669473 0.5503284005567934

0.8532300000000035 0.7427199999999985 0.4096199999999968

0.6186713624027931 0.4926538738281394 0.5503195472164606

0.8559997471249616 0.7530426909855734 0.5249684611594887

0.8962750291770760 0.1213986988288696 0.6031512799486407

0.8532300000000035 0.1474499999999992 0.4626700000000028

0.5806299999999993 0.1209899999999990 0.4729299999999981

0.0789918596241890 0.2374396983129543 0.5389851574951763

0.3955523242970216 0.0090822687611833 0.5347544314857373

0.3564210809313693 0.2528638789268527 0.5246522023115787

0.5806299999999993 0.7611500000000007 0.3948899999999966

0.8900900000000007 0.9888499999999993 0.3993600000000015

0.3914047100071514 0.3761485861726186 0.6131019130631614

0.0806299999999993 0.8790100000000010 0.4605099999999993

0.8192144574909506 0.6370630802667563 0.6142683329255831

0.3964330471841464 0.6213245899684523 0.6032997004062963

0.6255200000000016 0.6065699999999978 0.4604300000000023

0.6255200000000016 0.2387000000000015 0.3868599999999986

0.8451999999999984 0.5112999999999985 0.4073800000000034

0.8352449351977356 0.3603735516072106 0.6077273978053341

0.6255200000000016 0.8934300000000022 0.4730100000000022

0.6279123800842115 0.2641715154146453 0.5455753305526656

0.8507408487742936 0.9889920223496489 0.5231599874389303

0.7331989415687090 0.7775862389698887 0.7393424123771908

0.7155189835286181 0.7698488960419710 0.6958279680298293

Atomic coordinates of HCOOH* adsorption on the In_2_O_3_ (110) surface model

1.00000000000000

10.2365999999999993 0.0000000000000000 0.0000000000000000

0.0000000000000000 14.4766999999999992 0.0000000000000000

0.0000000000000000 0.0000000000000000 25.9995000000000012

In O C H

32 50 1 2

Direct

0.7013999999999996 0.2500000000000000 0.4667200000000022

0.4842238085043249 0.3742548808901347 0.5376073421004506

0.7680024818388986 0.9979081538209797 0.6010177717727672

0.9844428709814750 0.8729680153170989 0.5382451038414757

0.4853600000000000 0.1250000000000000 0.3971200000000010

0.9853600000000000 0.6250000000000000 0.3971200000000010

0.2744758676351395 0.0010420735157695 0.6021726518328023

0.4853600000000000 0.6250000000000000 0.3971200000000010

0.2013999999999996 0.2500000000000000 0.4667200000000022

0.9853600000000000 0.1250000000000000 0.3971200000000010

0.4865407280742318 0.8747230074173729 0.5384537969580450

0.9847367952035100 0.3736595528639910 0.5402296691541224

0.4877398449992398 0.6070959878840512 0.5285683446162253

0.9961313329658879 0.6383224033866952 0.5466055883176182

0.9853600000000000 0.3919799999999967 0.4065799999999982

0.4853600000000000 0.3580200000000033 0.3876700000000000

0.2409193434009751 0.2715366679504854 0.5952049115153741

0.2353600000000000 0.9830200000000033 0.4761799999999994

0.2551086239928608 0.7271615779353340 0.6058816308244062

0.2353600000000000 0.5169799999999967 0.4572700000000012

0.2013999999999996 0.7500000000000000 0.4667200000000022

0.2670818314470651 0.4974533456964778 0.6013072741410568

0.7590832272643411 0.4907687275869037 0.6075826518882508

0.7013999999999996 0.7500000000000000 0.4667200000000022

0.9881358748776705 0.1068600130001940 0.5285442885763487

0.4887863401337711 0.1414062268376384 0.5484646780818494

0.4853600000000000 0.8919799999999967 0.4065799999999982

0.9853600000000000 0.8580200000000033 0.3876700000000000

0.7351673603456845 0.7747304698906490 0.5957556970206781

0.7353600000000000 0.4830200000000033 0.4761799999999994

0.7557233755385582 0.2238861338171887 0.6047323192405969

0.7353600000000000 0.0169799999999967 0.4572700000000012

0.0806299999999993 0.6209899999999990 0.4729299999999981

0.5769156983859745 0.7386573829608594 0.5385835485380355

0.8874408782890697 0.5132440679707670 0.5414088966848283

0.6265526540288491 0.1082795574715192 0.6092149549930070

0.0806299999999993 0.2611500000000007 0.3948899999999966

0.8532300000000035 0.3525500000000008 0.4707799999999978

0.3900900000000007 0.4888499999999993 0.3993600000000015

0.8882670335669403 0.8751657931009404 0.6127583809948405

0.5806299999999993 0.3790100000000010 0.4605099999999993

0.3174759712443134 0.1390290401591869 0.6145885338646409

0.1174899999999965 0.5072800000000015 0.3846199999999982

0.1255200000000016 0.1065699999999978 0.4604300000000023

0.1255200000000016 0.7387000000000015 0.3868599999999986

0.3451999999999984 0.0112999999999985 0.4073800000000034

0.6197342368678136 0.8957394829228278 0.5999455289854083

0.3330200960947565 0.8611760329660854 0.6076719827621986

0.1255200000000016 0.3934300000000022 0.4730100000000022

0.1284623069173989 0.7637607547029148 0.5452515112601120

0.3493964771064810 0.4894681164528976 0.5231913233910888

0.3532300000000035 0.2427199999999985 0.4096199999999968

0.3532300000000035 0.8525500000000008 0.4707799999999978

0.1224300314856208 0.3931747779361316 0.6006852930321145

0.3532300000000035 0.6474499999999992 0.4626700000000028

0.1231343968158995 0.6106447420329459 0.6104020451721937

0.6174899999999965 0.0072800000000015 0.3846199999999982

0.1179663995203910 0.9910480456032502 0.5503815946371375

0.8532300000000035 0.7427199999999985 0.4096199999999968

0.6169965297923099 0.4918986351136141 0.5493313975384666

0.8548319654850758 0.7454728595976547 0.5279310053199566

0.8968703101936768 0.1188896573880598 0.6030058555487372

0.8532300000000035 0.1474499999999992 0.4626700000000028

0.5806299999999993 0.1209899999999990 0.4729299999999981

0.0788294919666797 0.2374917647639450 0.5389184732085610

0.3941575500115277 0.0099841644790999 0.5345319998181353

0.3555902794541694 0.2528470810337424 0.5243218011232145

0.5806299999999993 0.7611500000000007 0.3948899999999966

0.8900900000000007 0.9888499999999993 0.3993600000000015

0.3900579208589861 0.3762491810844182 0.6124588267579654

0.0806299999999993 0.8790100000000010 0.4605099999999993

0.7681170481108737 0.6414099749489850 0.6270755913571406

0.3928705294855419 0.6214207094991195 0.6023921243111303

0.6255200000000016 0.6065699999999978 0.4604300000000023

0.6255200000000016 0.2387000000000015 0.3868599999999986

0.8451999999999984 0.5112999999999985 0.4073800000000034

0.8378213109038910 0.3540269278343189 0.6027056427340511

0.6255200000000016 0.8934300000000022 0.4730100000000022

0.6258289656419933 0.2631274730510214 0.5453026597599191

0.8510788895061268 0.9874617610949059 0.5228448804617827

0.6458129012263427 0.6103920010240813 0.7124076624915929

0.6726542347806372 0.4639018635071253 0.6811761321330110

0.6296785322418970 0.5250575751871835 0.7133365004561796

0.5721422595234671 0.4942456376363680 0.7452274659017064

0.7264747230436086 0.6374374485177583 0.6623342805628997

Atomic coordinates of a CO_2_ molecule

1.00000000000000

20.0000000000000000 0.0000000000000000 0.0000000000000000

0.0000000000000000 20.0000000000000000 0.0000000000000000

0.0000000000000000 0.0000000000000000 20.0000000000000000

C O

1 2

Direct

0.5013244507187830 0.5000000000000000 0.5000135481165583

0.5004651923078204 0.5000000000000000 0.5588442076230138

0.5021803569733919 0.5000000000000000 0.4411922442604295

Atomic coordinates of a CO molecule

1.00000000000000

20.0000000000000000 0.0000000000000000 0.0000000000000000

0.0000000000000000 20.0000000000000000 0.0000000000000000

0.0000000000000000 0.0000000000000000 20.0000000000000000

C O

1 1

Direct

0.5001068235254951 0.5000000000000000 0.5091615983483031

0.5007731764745071 0.5000000000000000 0.5663284016516990

Atomic coordinates of a HCOOH molecule

1.00000000000000

20.0000000000000000 0.0000000000000000 0.0000000000000000

0.0000000000000000 20.0000000000000000 0.0000000000000000

0.0000000000000000 0.0000000000000000 20.0000000000000000

C O H

1 2 2

Direct

0.4941546272256137 0.5000000000000000 0.5054046253947119

0.5074953235719312 0.5000000000000000 0.5645571702955210

0.4314768169588340 0.5000000000000000 0.4794890587891185

0.5310676611852176 0.5000000000000000 0.4642384764464524

0.4003355710584058 0.5000000000000000 0.5174806690741990

Atomic coordinates of a H_2_O molecule

1.00000000000000

20.0000000000000000 0.0000000000000000 0.0000000000000000

0.0000000000000000 20.0000000000000000 0.0000000000000000

0.0000000000000000 0.0000000000000000 20.0000000000000000

H O

2 1

Direct

0.5000000000000000 0.5121549295311512 0.4556300898179873

0.5000000000000000 0.5022767388989007 0.5318769084154198

0.5000000000000000 0.5367183315699483 0.4975830017665925

Atomic coordinates of a H_2_ molecule

1.00000000000000

20.0000000000000000 0.0000000000000000 0.0000000000000000

0.0000000000000000 20.0000000000000000 0.0000000000000000

0.0000000000000000 0.0000000000000000 20.0000000000000000

H

2

Direct

0.5000000000000000 0.5000000000000000 0.4997399407526373

0.5000000000000000 0.5000000000000000 0.5372600592473616
